# Chemical control of spin–lattice relaxation to discover a room temperature molecular qubit†

**DOI:** 10.1039/d1sc06130e

**Published:** 2022-05-17

**Authors:** M. Jeremy Amdur, Kathleen R. Mullin, Michael J. Waters, Danilo Puggioni, Michael K. Wojnar, Mingqiang Gu, Lei Sun, Paul H. Oyala, James M. Rondinelli, Danna E. Freedman

**Affiliations:** Department of Chemistry, Massachusetts Institute of Technology Cambridge Massachusetts 02139 USA danna@mit.edu; Department of Materials Science and Engineering, Northwestern University Evanston Illinois 60208 USA jrondinelli@northwestern.edu; Center for Nanoscale Materials, Argonne National Laboratory Argonne Illinois 60439 USA; Division of Chemistry and Chemical Engineering, California Institute of Technology Pasadena California 91125 USA; Department of Chemistry, Northwestern University Evanston Illinois 60208 USA

## Abstract

The second quantum revolution harnesses exquisite quantum control for a slate of diverse applications including sensing, communication, and computation. Of the many candidates for building quantum systems, molecules offer both tunability and specificity, but the principles to enable high temperature operation are not well established. Spin–lattice relaxation, represented by the time constant *T*_1_, is the primary factor dictating the high temperature performance of quantum bits (qubits), and serves as the upper limit on qubit coherence times (*T*_2_). For molecular qubits at elevated temperatures (>100 K), molecular vibrations facilitate rapid spin–lattice relaxation which limits *T*_2_ to well below operational minimums for certain quantum technologies. Here we identify the effects of controlling orbital angular momentum through metal coordination geometry and ligand rigidity *via* π-conjugation on *T*_1_ relaxation in three four-coordinate Cu^2+^*S* = ½ qubit candidates: bis(*N*,*N*′-dimethyl-4-amino-3-penten-2-imine) copper(ii) (Me_2_Nac)_2_ (1), bis(acetylacetone)ethylenediamine copper(ii) Cu(acacen) (2), and tetramethyltetraazaannulene copper(ii) Cu(tmtaa) (3). We obtain significant *T*_1_ improvement upon changing from tetrahedral to square planar geometries through changes in orbital angular momentum. *T*_1_ is further improved with greater π-conjugation in the ligand framework. Our electronic structure calculations reveal that the reduced motion of low energy vibrations in the primary coordination sphere slows relaxation and increases *T*_1_. These principles enable us to report a new molecular qubit candidate with room temperature *T*_2_ = 0.43 μs, and establishes guidelines for designing novel qubit candidates operating above 100 K.

## Abstract 2

Elucidating the role of specific vibrational modes in spin lattice relaxation is a key step to designing room temperature qubits. We executed an experimental and theoretical study on a series of Cu^2+^ qubits to increase their operating temperature.

## Graphical abstract

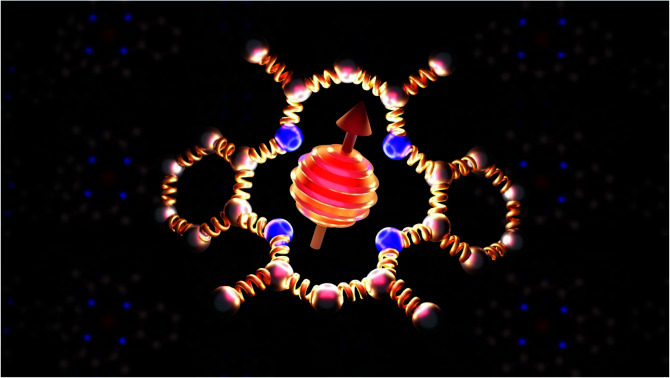

## Introduction

The second quantum revolution is transforming our world with technological advances in many fields ranging from quantum computation, quantum communication, and quantum sensing.^1–5^ Progress in quantum information science (QIS) is driven by improvements in its foundational unit of information – the quantum bit (qubit). A qubit is a two-level system that exists in either of its two states or in an arbitrary superposition of them.^2^ Potential qubit candidates cover a wide range of materials, and take advantage of a myriad of quantum particles^1^ including Cooper pairs in superconductors,^6–8^ the nuclear states of trapped ions,^9,10^ and the polarization of photons.^11,12^ Electronic spins have demonstrated great promise as qubits for quantum sensing applications,^13–16^ because they combine strong coupling to the local environment with spatial precision to enable high resolution and high precision microscopy and metrology.^17–21^ Solid-state defect systems such as the anionic nitrogen-vacancy center are widely studied, owing in part to their long coherence times (described by the parameter *T*_2_) that persist to room temperature.^22^ In recent years, molecular electronic spins have emerged as a novel platform with atom-by-atom, bottom-up control over qubit structure and the local spin environment, allowing for direct control over qubit properties. The power of designer qubits has advanced molecular QIS on many fronts including: direct synthetic control over coherence times,^23–26^ scaling into multiqubit arrays,^27–30^ and incorporation of a designable optical interface.^31^ These studies paved the way for creating new molecular qubit candidates with millisecond coherence times, and approaches to integrate molecular qubits with device architectures for sensing.^14,15^ Historically, these results are primarily limited to low temperatures (<5 K). The coherence of few qubit candidates have maintained operationally useful coherence times (>0.2 μs) at elevated temperatures, with *T*_2_ falling off as temperature increases.^32,33^

This low temperature limitation prevents the investigation of molecular qubits in technologies they are otherwise well suited for, such as *in vivo* biological quantum sensors. In these devices, the high sensitivity of the electron coupled with atom-by-atom design of spin arrays would enable high precision detection of dangerous toxic agents, and nanoscale mapping of 3D protein structure.^34–39^ Unfortunately, few transition metal qubits remain measurable above 200 K, with substantially fewer remaining operable out to physiological temperatures.^13,25,40–42^ While *T*_2_ is largely considered temperature insensitive, it begins to decrease at higher temperatures due to a second parameter – the spin–lattice relaxation time *T*_1_. *T*_1_ represents the relaxation of a spin population from an excited state, such as a superposition state, back to thermal equilibrium.^43,44^ Since coherence cannot exist out of the superposition state, this places a fundamental limit on *T*_2_ where 2*T*_1_ ≥ *T*_2_.^23^ Unlike *T*_2_, *T*_1_ is strongly temperature dependent – decreasing as high coupling vibrational modes become more occupied at higher temperature.^45–47^ All quantum systems eventually reach the limit where 2*T*_1_ = *T*_2_ and *T*_2_ decreases with decreasing *T*_1_. Maximizing *T*_2_ at high temperatures requires maximizing *T*_1_.

**Fig. 1:**
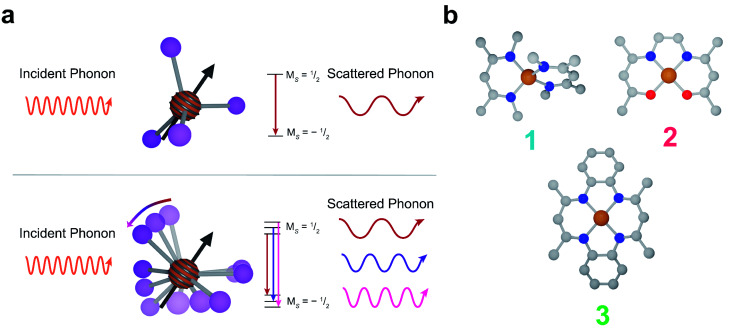
(a) (Top) A representation of the phonon interactions in the Raman process. (bottom) A representation of phonon interactions in the local modes process. (b) Molecular structures of 1, 2, and 3. Grey, blue, red, and orange represent carbon, nitrogen, oxygen and copper, respectively. Hydrogen atoms are omitted for clarity. 1 is best described as pseudo-tetrahedral, whereas 2 and 3 are square planar.

Spin–lattice relaxation arises from vibrational modes in the system facilitating the release of energy from a non-equilibrium spin population to return the system to equilibrium.^48^ Under standard experimental conditions, the energy gap between spin sublevels is less than 10 GHz (0.3 cm^−1^). The only pathway available for molecular systems to release such small energy quanta are low energy lattice modes called phonons. At low temperatures, phonons relax molecular spins through a scattering process where an incident phonon scatters off the spin, facilitating the release of energy. At higher temperatures, local molecular vibrations become thermally populated and distort the molecular geometry. This distortion increases electron spin relaxation by modulating Zeeman splitting (Fig. 1a).^43,49–51^

Above the temperatures where molecular vibrations are thermally occupied, they are the predominant pathway for spin–lattice relaxation. The *T*_1_-limited regime of coherence for *S* = ^1^/_2_ systems typically occurs under local mode dominated relaxation. The impact of modifying vibrational modes, therefore, is felt most strongly at high temperatures. Restricting or modifying vibrations which cause relaxation allows us to control relaxation rates at these temperatures. In molecular qubits, our enhanced control over metal-coordination geometry and ligand structure gives us a direct method for controlling these local vibrational modes. Previous work has investigated the role of metal coordination geometry in V^4+^ electronic spin qubits.^52,53^ These studies found strong correlations between coordination complex geometry and spin–lattice relaxation rates. However, due to the localization of the unpaired electron in V^+4^ to a nominally nonbonding orbital (minimizing the spin delocalization onto the ligand),^40,54^ these studies were unable to address the role of ligand structure in relaxation rates.

In this report, we demonstrate deliberate control of the molecular vibrations in three molecular systems: bis(*N*,*N*′-dimethyl-4-amino-3-penten-2-imine) copper(ii) (Cu(Me_2_Nac)_2_ (1)),^55,56^ bis(acetylacetone)ethylenediamine copper(ii) (Cu(acacen) (2)),^57^ and tetramethyltetraazaannulene copper(ii) (Cu(tmtaa) (3))^58–60^ (Fig. 1b). Both 1 and 3 can be viewed as chemical modifications to 2. In 1, the breaking of an ethylene linker, as well as additional steric interaction through the presence of *N*-methyl groups enforces a distorted tetrahedral geometry. In contrast, 2 and 3 are locally square planar complexes. 3 maintains the same general structure of 2, but with additional rigidity imparted by increased π-conjugation and complete cyclization of the ligand. Additionally, by changing the spin active metal to Cu^2+^, we significantly increase the ligand delocalization of our electronic spin, allowing us to directly interrogate the role of ligand structure on relaxation.

## Results and discussion

We first quantify the geometry of each complex through the *τ*_4_′ parameter, which represents the distortion of a four coordinate metal complex on a scale from 0 (square planar) to 1 (tetrahedral).^61^ The *τ*_4_′ value of 1 shows that it is pseudo-tetrahedral (*τ*_4_′ = 0.62). 2 and 3 are nearly perfectly square planar (*τ*_4_′ = 0.066 and 0.052 respectively). The relative rigidity of the molecules from weakest to strongest is 1 < 2 < 3 (Fig. S1†). The different geometries result in a weaker (less covalent) metal–ligand interaction and a lower energy singly occupied molecular orbital (SOMO) in 1 relative to 2 and 3 (Tables S8–S10†).^62^ The lower covalency in 1 compared to 2 and 3 is also reflected in their Cu–N bond lengths. 1 has an average Cu–N bond length of 1.955 Å, compared to the Cu–N bond length of 1.924 Å in 2 and 1.930 Å in 3. The decreased π rigidity, as well as the lack of a tethering functional group makes 1 the least rigid qubit candidate. 2 is more flexible than 1 due to a combination of stronger bonding and its tethering ethylene group. 3 is the most rigid of the three molecules due to rigidity from its π-conjugated ligand (Fig. S1†).

Next, we probed the magnetic structures of the three complexes using continuous-wave electron paramagnetic resonance (CW-EPR) spectroscopy. EPR spectroscopy tells us not only the energy of the Zeeman interaction, but also gives us important information about the strength of spin–orbit coupling (SOC) and orbital angular momentum (OAM) – two critical factors in determining how both lattice and molecular vibrations interact with a spin. SOC allows orbital perturbations (such as lattice phonons and molecular vibrations) to impact the spin moment. In the limit of zero SOC, orbital perturbations do not influence spins, and therefore vibrations cannot cause relaxation. The spin–orbit interaction is described by the term *λ*(*Ŝ*·*L̂*). In a crystal field, the d orbitals split in energy, and in the absence of OAM, *L̂* is zero. In real molecular systems, second order OAM is recovered through the interaction between the molecular ground state with low energy excited states, allowing for a non-zero spin–orbit interaction. An in-depth understanding of how these systems should relax, therefore, necessitates an understanding of these parameters.

**Fig. 2:**
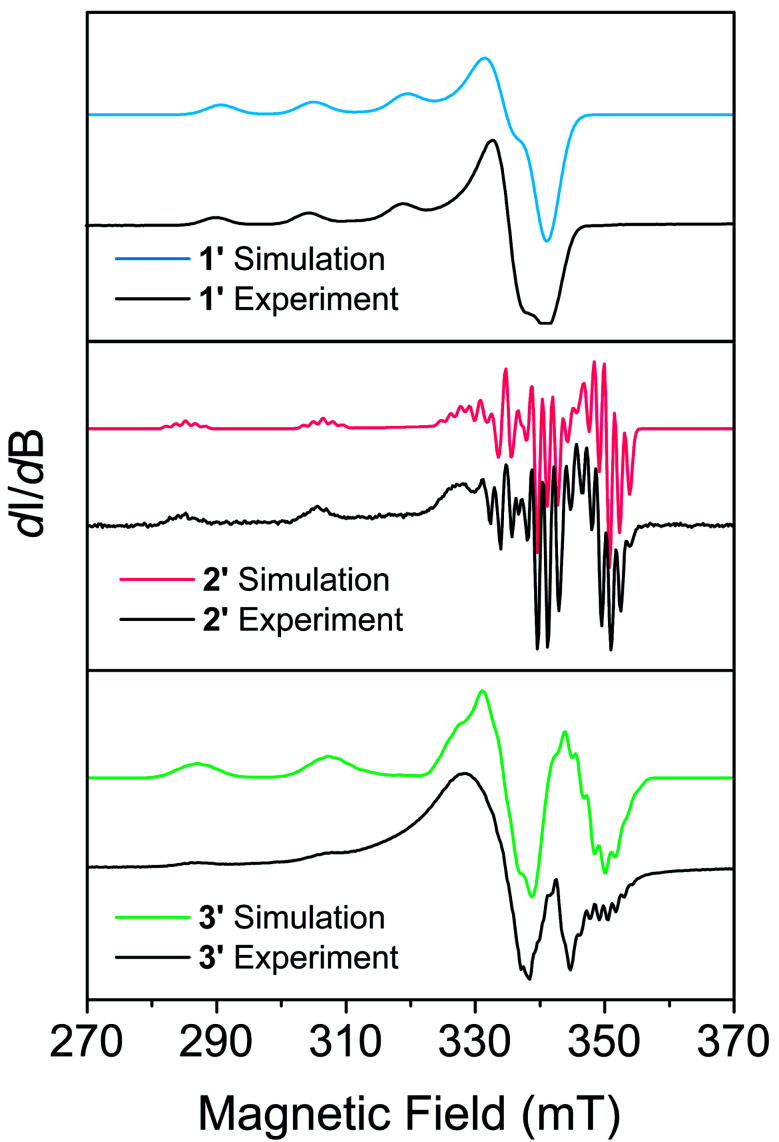
CW-EPR spectra of 1′–3′ taken at X-band (∼9.5 GHz), 10 K.

EPR measurements were performed on powder samples of 1–3 diluted to 1% by weight in a diamagnetic analogue (Zn(Me_2_Nac)_2_ (4) for 1, Ni(acacen) (5) for 2, and Ni(tmtaa) (6) for 3), denoted 1′–3′ respectively. Fig. 2 shows the CW-EPR spectra of 1′–3′ at 10 K. Simulations of all spectra were performed using a spin Hamiltonian *Ĥ* = *gμ*_B_*BS* + *IAS*, where *g* is the *g*-tensor for the spin, *μ*_B_ is the Bohr magneton, *B* is the magnetic field, *S* is the electronic spin, *I* is a matrix with the nuclear spin of the metal and the atoms directly bound to the metal, and *A* is the nuclear hyperfine tensor, respectively, using the program Easyspin.^63^ Table S11† provides the parameters which best simulate the spectra for 1′–3′. Additional details on simulating CW-EPR spectra, and comments on the effects of broadening in our spectra, can be found in the ESI.† All three complexes are best simulated as axial copper systems such that *g*_*x*_ = *g*_*y*_ ≠ *g*_*z*_ (for axial systems the equivalent *g*_*x*_ = *g*_*y*_ pair is termed g_⊥_ and the *g*_*z*_ component is termed *g*_∥_) with hyperfine interactions from the copper and the nitrogen atoms. The parameters we obtain are comparable to other four-coordinate copper complexes in similar geometries.^27,41,43^ We see a clear dependence of *g*_∥_ on the geometry of our complexes: the square planar 2′ and 3′ are within simulation error (*g*_∥_ = 2.17(1) and *g*_∥_ = 2.175(1) respectively) and pseudo-tetrahedral 1′ is greater (*g*_∥_ = 2.205(5)). We attribute this to OAM contributions arising from a spin–orbit coupling effect that allows mixing with low-lying excited states.^43^ The aforementioned lower-lying excited states in 1′ mix more strongly, recovering more OAM and causing a greater deviation from the free electron *g* value. Coupled with the decreased rigidity around the metal center, we would expect 1′ to have more rapid spin–lattice relaxation (*i.e*. a shorter *T*_1_) than 2′ or 3′. 2′ and 3′ have similar OAM contributions, but the slightly increased rigidity of 3 makes it likely relax slower.^25^

**Fig. 3:**
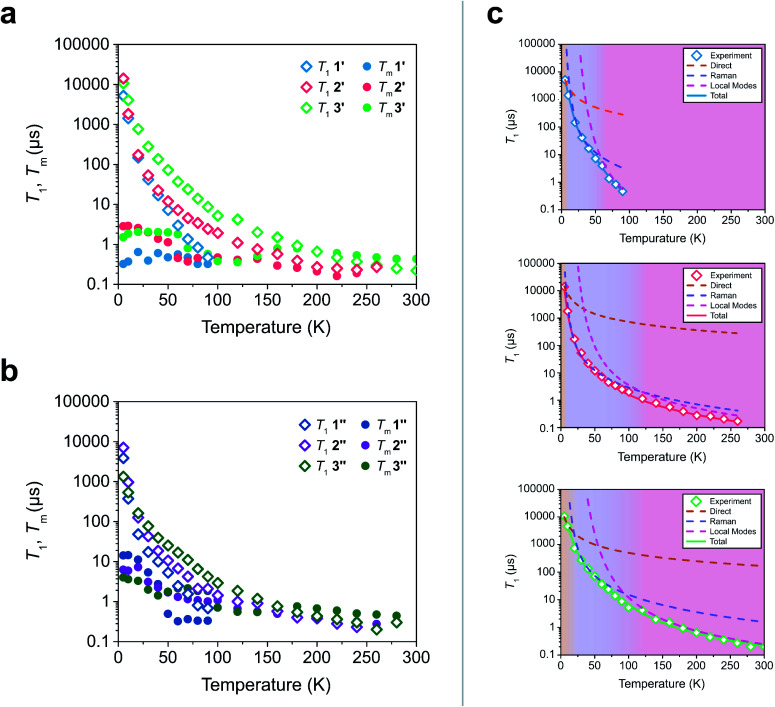
(a and b) Relaxation time constants extracted from pulse-EPR spectroscopy for complexes diluted in a diamagnetic analogue (1′–3′) and in OTP (1′′–3′′). *T*_1_ was obtained through saturation recovery, wherein a train of short microwave pulses saturates the transition and the return to equilibrium is monitored. *T*_m_ was obtained through a two-pulse Hahn echo experiment, wherein coherence loss in the superposition state is monitored. (c) Fits of the *T*_1_ relaxation data for 1′–3′ to eqn (1). The shaded regions correspond to the process which is dominant in that temperature regime.

To test these hypotheses, we directly measured the relaxation dynamics of the three systems with pulse-EPR spectroscopy. We pulsed each system at its peak of maximum intensity in their echo-detected field swept EPR spectra (Fig. S10–S12†). As these measurements were performed on ensembles where each electron spin is not isolated from additional magnetic interactions (such as nearby electronic spins), measurement of an intrinsic *T*_2_ is not possible. We instead measure the phase memory time *T*_m_, which is the decay constant for all sources of dephasing, not just spin–spin interactions.^48^ We wish to highlight that the previously described relationships between *T*_1_ and *T*_2_ are approximately true for *T*_1_ and *T*_m_ (namely *T*_m_ < 2*T*_1_). The *T*_1_ and *T*_m_ relaxation times across temperature for 1′–3′ are given in Fig. 3. As previously discussed, local vibrations and lattice vibrations both contribute to *T*_1_ relaxation. In order to deconvolute the impact of local vibrations and lattice vibrations, we compared all measurements performed on 1′–3′ to analogous systems made through dissolution in the room temperature glass *ortho*-terphenyl (OTP) denoted 1′′–3′′. A further discussion on the role the matrix plays in deconvoluting matrix effects can be found in the ESI.†

At low temperatures, trends in *T*_1_ depend most strongly on the matrix. The *T*_1_ of the crystalline solids (1′–3′) are all larger than the low temperature *T*_1_ of their amorphous glass analogues (1′′–3′′). Glasses have a higher density of low energy phonons, which have a better energy match to the Zeeman transition of the spin center and therefore promote faster relaxation in the phonon-dominated temperature regime.^68–70^ As temperature increases, the discrepancy between the matrices decreases, with the behaviour of each complex in both matrices hitting a near coalescence point (around 50 K for 1 and 2, and around 200 K for 3). The eventual near identical relaxation behaviour highlights the importance of the different types of vibrations in different temperature regimes: at low temperatures, the phonon modes of the matrix dominate relaxation behaviour. At high temperature, relaxation becomes dominated by molecular vibrations. Above 60 K, the *T*_1_ relaxation of 1′ and 1′′ becomes much faster than the relaxation of 2′–3′ or 2′′–3′′. By 80 K the *T*_1_ of 1′ and 1′′ are less than 1 μs whereas the *T*_1_ of 2′ and 2′′ are approximately 3.5 μs and the *T*_1_ of 3′ and 3′′ are both greater than 6 μs. By 100 K, the *T*_1_ of 1′ is undetectably fast (<0.15 μs). The *T*_1_ of 2′ and 2′′ remain measurable out to 260 K (*T*_1_ = 0.27 μs and *T*_1_ = 0.20 μs respectively). Similar to what was noted for *T*_m_, the *T*_1_ of 3′′ remains measurable out to 280 K (*T*_1_ = 0.3 μs) but was undetectable at higher temperatures and the *T*_1_ of 3′ was measurable at room temperature (*T*_1_ = 0.22 μs). We note that in all six measured systems, the highest temperature at which coherence is measurable is limited by the *T*_1_ time of the system, as we would expect from the fundamental *T*_2_ < 2*T*_1_ limit, now applied to *T*_m_.

Although 1 was the fastest relaxing qubit, as predicted from the recovered OAM, the large difference between 2 and 3 could not be understood from orbital momentum alone. As discussed previously, molecular vibrations provide additional mechanisms to relax the spin, but are only operative at high temperatures. The difference in the high temperature relaxation of 2 and 3 must then originate from these molecular vibrations. To gain insight into these high temperature dynamics, we modelled the temperature dependence of *T*_1_ to account for contributions from three relaxation processes: (1) the direct process, corresponding to single phonon emission, (2) the Raman process where an incident phonon scatters off of a spin center to facilitate relaxation, and (3) a local mode mediated process that occurs *via* modulation of magnetic parameters.^71^ In the literature, the terms “phonon” and “molecular vibration” are frequently used interchangeably. In order to better contextualize our discussion, we use the terms “phonon” and “lattice mode” to exclusively refer to lattice vibrations, whereas “molecular vibration” and “local mode” will exclusively refer to local molecular distortions. Understanding the different temperature regimes and energy scales of these two processes is imperative to understanding their role in relaxation. Molecular vibrations involved in the local modes process are specifically modulations of these phonon interactions. Therefore, we fit the relaxation data with the standard Debye model derived equation as follows:1
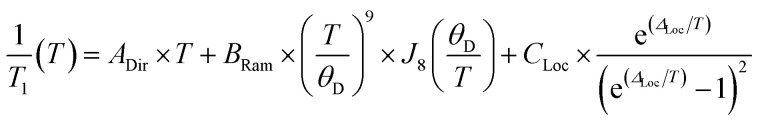
where *A*_Dir_, *B*_Ram_, and *C*_Loc_ are the coefficients of the direct, Raman, and local modes processes, and are interpreted as weighting coefficients to represent the number of relaxation events caused by the process per unit time. Additional details of *T*_1_ fitting are provided in the ESI.†

Although *A*_Dir_, *B*_Ram_, and *C*_Loc_ are best fit parameters, molecular correlations have been attributed to changes in these parameters as follows: *A*_Dir_ is associated with the low energy phonon density-of-states for a compound.^48^ Both *B*_ram_ and *C*_Loc_ arise from SOC interactions and second-order orbital momentum.^48,72^*C*_Loc_ is additionally weighted by the spin–phonon coupling (SPC) of the various local vibrational modes.^49^ Since each local mode uniquely distorts the molecular geometry, each mode has a unique SPC coefficient representing its impact on spin relaxation. To prevent overparameterization of the system, we used a generalized local modes term, which is an average of all modes in the system weighted by their SPC (*Δ*_Loc_), and a generalized *C*_Loc_ coefficient representing the average impact of all modes in *Δ*_Loc_.^48,49^*Δ*_Loc_ is highly correlated with the rigidity of the metal–ligand bond, as well as ligand rigidity from π-conjugation.^40,73^*Θ*_D_ is the Debye temperature of the matrix for the spin center, and scales the Raman process by the phonon energy of the matrix.^44^ In molecular systems, *Θ*_D_ is not a true Debye temperature; it is better understood as a proxy for lattice phonon energy of the molecular crystal. Fits to the relaxation data for 1′–3′ are shown in Fig. 3c, and the fit parameters for these fits are given in Table 1 (fits and parameters for 1′′–3′′ can be found in the ESI† as well as a complete discussion on the differences in fit parameters between matrices). We limit the following discussion to the crystalline solids 1′–3′, but note that all discussed trends are also observed in 1′′–3′′.

**Table 1:** Debye Model Fit Parameters for 1′–3′

	1′	2′	3′
*A*_Dir_	39(4)	13.8(4)	18(1)
*B*_Ram_	15(5)	7(2)	2.7(2)
*Θ*_D_	75(10)	63(8)	81(4)
*C*_Loc_	18.5(9)	0.6(1)	1.1(1)
*Δ*_Loc_	290(40)	213(25)	328(15)

Eqn (1) provides an excellent fit for the temperature-dependent relaxation of 1′, 2′, and 3′, as seen in Fig. 3. The *B*_ram_ coefficients also reflect the information extracted from their CW-EPR spectra. 1′ experiences more second-order OAM from low lying excited states relative to 2′ and 3′, so it has a higher *B*_Ram_. The *B*_Ram_ of 2′ and 3′ are similar, suggesting each exhibit similar OAM. This follows the expected trend based on ligand field strengths in the two complexes (Table S8–S10†). We find a similar trend in *C*_Loc_ where *C*_Loc_ of 1′ is significantly greater than 2′ and 3′ by one to two orders of magnitude (18.5 × 10^7^ s^−1^ in 1′*versus* 0.6 × 10^7^ s^−1^ in 2′ and 1.1 × 10^7^ s^−1^ in 3′), also suggesting increased OAM.

Surprisingly, the *Δ*_Loc_ parameter of 1′ (*Δ*_Loc_ = 290 cm^−1^) is higher than that of 2′ (*Δ*_Loc_ = 213 cm^−1^), despite 1 being the less rigid molecule. This implies that the rapid relaxation of 1′ is not driven by an abundance of low energy vibrational modes, but instead must be driven by the large OAM of 1′ driving inherently faster relaxation. The significant difference in high temperature relaxation between 1′ and 2′–3′ is then an effect of OAM by similar logic. The difference in relaxation between 2′ and 3′ cannot be explained through OAM, but it can be attributed to differences in molecular rigidity. The *Δ*_Loc_ parameter of 3′ is the largest of the three complexes (328 cm^−1^), whereas the *Δ*_Loc_ of 2 is the smallest (213 cm^−1^). This suggests that because 2 is more flexible than 3, the vibrations which cause relaxation are lower in energy. Therefore, they are thermally occupied at lower temperatures, and drive faster local mode mediated relaxation. Because the low energy of vibrational modes in 2 does not drive its relaxation to be faster than 1, but does drive its relaxation to be faster than 3, we conclude that OAM effects are the primary factor dictating molecular relaxation – a molecule with less OAM will tend to relax slower, independent of its vibrational mode energy. Vibrational mode energy, then, is a secondary factor in determining relaxation rates. Between two systems with competitive OAM values, the one with the larger vibrational mode energy will tend to have longer *T*_1_ times out to higher temperatures.

Though the preceding analysis gives us the ability to qualitatively describe the effects of the changes to molecular structure on relaxation, it is unable to give us any information about the specific vibrations which relax electronic spins. In order to gain insight into the individual and collective impact of the vibrational modes in each complex, we performed density-functional theory calculations from which we quantify SPC through changes in the *g* and *A* tensor from excitation of each vibrational mode.^33,49,74^ Although full *ab initio* calculation of *T*_1_ been demonstrated,^49,75–78^ we use a proxy that does not include the computationally expensive single phonon correlation function that is included in these more complex models to understand the mode dependencies. Our proxy is defined as a sum of the derivatives of each component of the *g*-tensor squared with respect to each normal mode weighted by their Bose–Einstein occupation integrated over the relevant temperature range from experiment as:2
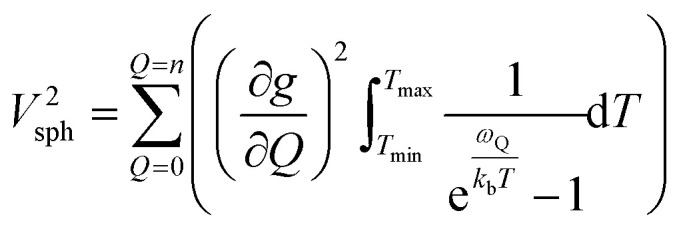


*V* ^2^_sph_ accounts for the impact of SPC by each mode using the thermodynamic occupancy of the mode at a given temperature. We emphasize that these calculations are performed on isolated single molecules. Therefore, the results of these calculations exclusively give us information about the modulation of relaxation rate from local mode distortions (*i.e.*, relaxation from the local modes process).

**Fig. 4:**
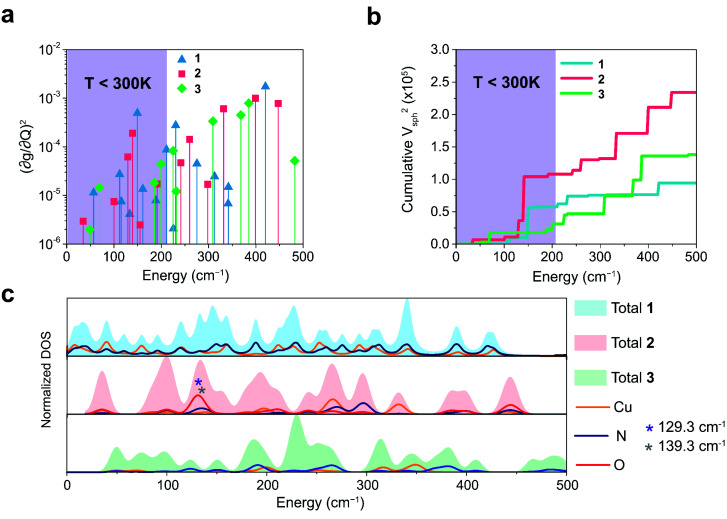
(a) Spin–phonon coupling decomposed by vibrational mode into spin–phonon-coupling coefficient 
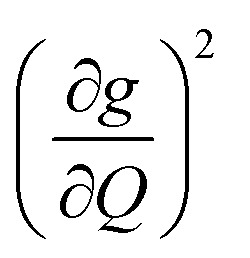
 between 0 and 500 cm^−1^ (modes with 
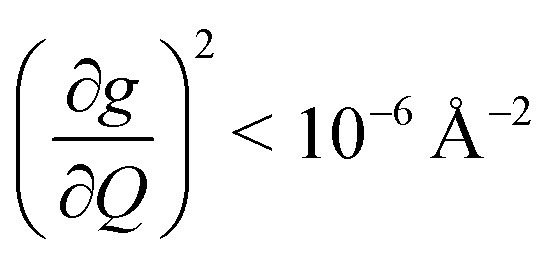
 are omitted for clarity). Values are expressed in units of Å^−2^ (b) total SPC (cumulative) of all modes with increasing energy. Large increases in SPC generally occur stepwise, with single highly coupled modes contributing heavily to SPC. Values are expressed in units of Å^−2^. In both (a) and (b), the energy range below 300 K (208.5 cm^−1^) corresponding to modes thermally occupied at room temperature, is highlighted in purple (c) atom-resolved vibrational density-of-states (DOS) for 1–3.

We plot both the SPC coefficient 
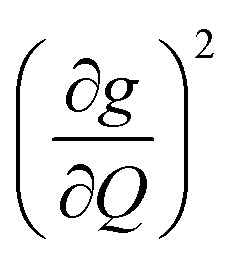
 of each mode and the cumulative, thermally weighted SPC (*V*^2^_sph_) as a function of energy in Fig. 4a and b. We first note that these calculations support previous conclusions that local mode relaxation is driven primarily by a small number of very highly coupled vibrational modes (Fig. S25†).^25,73,79^ The vast majority of vibrational modes have little to no impact on relaxation (a near zero 
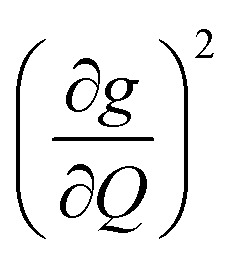
. In the energy range considered, each molecule has between three and four vibrational modes with 
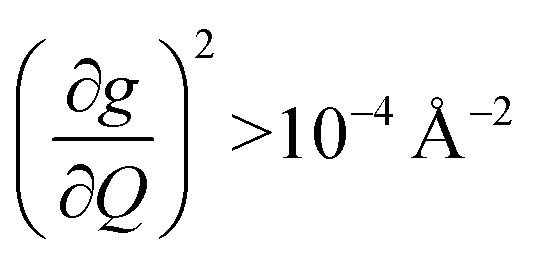
, and significantly more vibrations with orders of magnitude weaker coupling. Importantly, 3 has no thermally occupied vibrations above this cutoff by 300 K, whereas 1 and 2 both have a highly coupled mode in this low energy regime (in 1, 149.5 cm^−1^, 
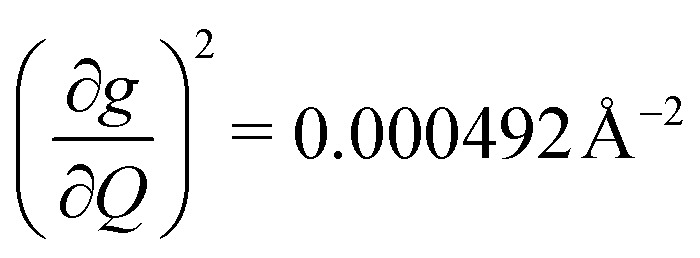
 and in 2, 139.3 cm^−1^
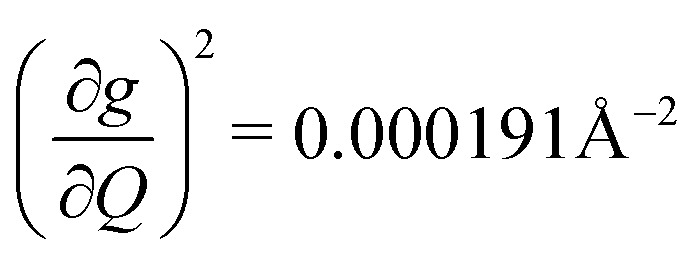
). We find that 2 has the largest *V*^2^_sph_ of all three complexes above 120 K, despite having measurable coherence out to significantly higher temperatures than 1. Additionally, two of the four most strongly coupled modes in 2 (399.1 cm^−1^, 
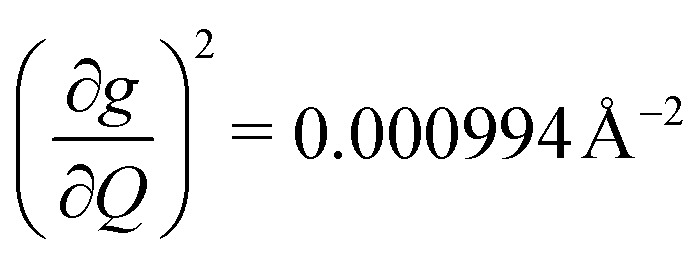
 and 447.2 cm^−1^, 
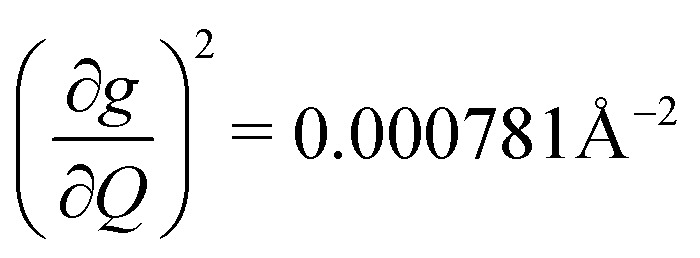
) are higher in energy than the most coupled mode in 3 (385.0 cm^−1^, 
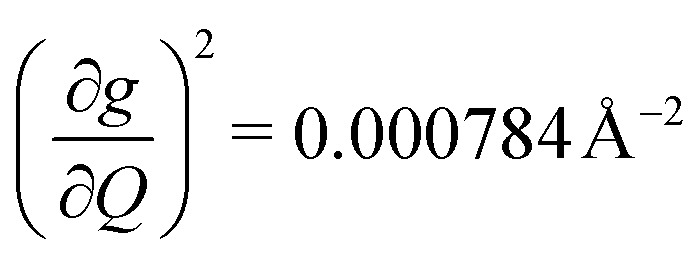
). Rapid relaxation in 2, then, is likely driven by the lower energy modes with large SPC constants: 129.3 cm^−1^, and 139.3 cm^−1^, 
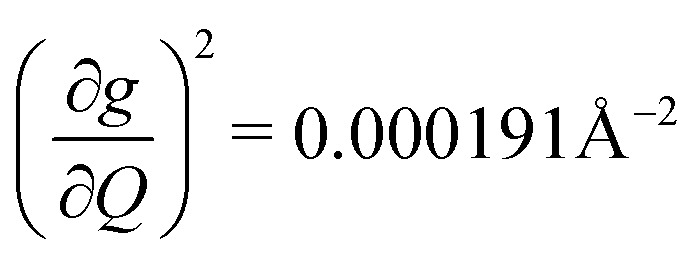
. These highly coupled low-energy modes are thermally occupied at much lower temperatures than the highly coupled modes in 3. These modes may also be responsible for the low *Δ*_Loc_ of 2 – low energy vibrations with significant SPC contribute to relaxation at a lower temperature, and are therefore more impactful to relaxation than low thermal occupancy modes with slightly higher SPC coefficients. By extension, the observed slow relaxation in 3 is result of having no thermally occupied modes with significant coupling at 300 K.

These results raise the question of why are there low energy modes in 2 to drive relaxation, but not in 3. To more deeply investigate this, we examined the atom-resolved partial vibrational density-of-states (PDOS) of 1–3 (Fig. 4c, more information can be found in the ESI†). These calculations decompose vibrational modes into the sum of atomic motion that comprises them and plots the amplitude of motion for each element in the molecule as a function of energy. The amplitude of each element's contribution to a mode then represents how much those atoms move during that particular vibration. For both 2 and 3, modes with large SPC coefficients also contain noticeably greater displacement of the primary coordination sphere (Cu, N, and O – represented by larger percentages of the DOS comprising these atomic motions) than other atoms in these complexes, with motions of the donor atoms (N and O) more strongly affected than the spin-bearing atom. Notably, the strongly coupled low-energy modes in 2 at 129.3 cm^−1^ and 139.3 cm^−1^ (
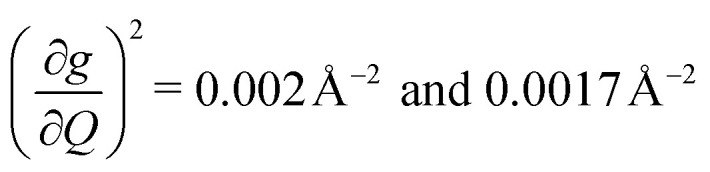
, respectively) mainly consist of motion in the untethered oxygen donor. This lack of tethering clearly allows a greater range of motion in the oxygen atoms (Fig. S29†), perturbing the local spin environment more, and resulting in faster relaxation. These two modes also appear to be responsible for the significant increase in *V*^2^_sph_, which also occurs at approximately 130 cm^−1^ (Fig. 4b).

The increased π rigidity of 3 appears to be reflected in both shifting modes involving the primary coordination sphere to higher energy, as well as reducing the involvement of those atoms in the mode. In other words, the chemical rigidity “stiffens” the atoms involved in the mode, increasing the energy required to activate them and reducing the motion of the atoms when they do vibrate. The importance we place on modes involving the primary coordination sphere is well captured by viewing the modes in each complex with the highest 
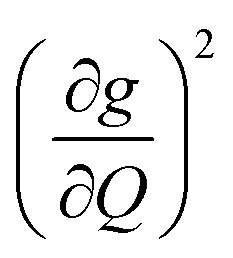
 term (Fig. S30†). In each mode, we see distortions which heavily involve moving donor atoms and the copper spin center.

We continued these studies by probing *T*_m_ to gain insight into the coherence properties of these systems. *T*_m_ was measured with a Hahn echo decay sequence. In all systems, *T*_m_ relaxation is impacted by additional decohering effects from the nuclear spins in the local environment. We note that for all three complexes, *T*_m_ is longer in OTP solution than in the crystalline solid. In 2 and 3, these differences are relatively small, with the 5 K *T*_m_ of 2 increasing from 2.82 μs in 2′ to 6.20 μs in 2′′, and the 5 K *T*_m_ increasing from 1.49 μs in 3′ to 3.98 μs in 3′′. These slight improvements are in line with the low nuclear spin density of OTP.^64^ The *T*_m_ improvement in 1 between the two matrices is significantly larger, with the 5 K *T*_m_ of 1′ (0.32 μs) being nearly 2 orders of magnitude smaller than the 5 K *T*_m_ of 1′′ (14.1 μs). We attribute this to the large number of protons on the Me_2_Nac ligand, leading to a nuclear spin rich spin environment (Fig. S8†). In OTP solution, the space between Me_2_Nac molecules becomes significantly larger, and the nearest protons are rather found on the solvent phenyl groups as opposed to the methyl groups of nearby Me_2_Nac units, dramatically increasing *T*_m_ at low temperatures.^65^ Beginning at 40 K, the *T*_m_ of 1′′ approaches the relaxation rate of 1′, with the two time constants being effectively equal by 50 K, likely as a result of the thermal activation of methyl rotation.^66^ The *T*_m_ of 1′ has already been significantly restrained by the high nuclear spin density of its local environment, so the additional decohering effect of methyl rotation is only weakly felt. In 1′′ however, methyl rotation becomes the strongest decohering effect, drastically shortening *T*_m_ until it approaches the *T*_m_ of 1′. The effects of methyl rotation are also observed in 2′–3′ and 2′′–3′′.

**Fig. 5:**
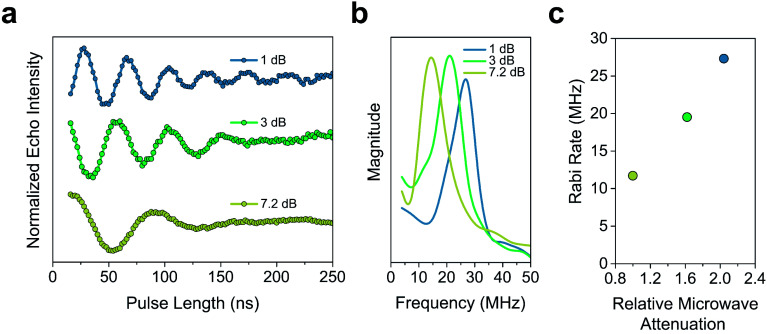
(a) Rabi oscillations of 3′ observed at 300 K at 3 different microwave attenuations. This hallmark of qubit behaviour demonstrates the continued operability of 3′, even at these high temperatures (b) Fourier transform of the 300 K Rabi oscillation in 3′. (c) Dependence of the Rabi rate of 3′ on the relative microwave attenuation at 300 K. The relative microwave attenuation is calculated relative to the weakest microwave power examined (7.2 dB).

The *T*_m_ of 2′ and 2′′ begins to decrease around 40 K and continues to decrease until it becomes too short to measure at 240 K (*T*_m_ = 0.16 μs) and 260 K (*T*_m_ = 0.27 μs), respectively. In 3′ and 3′′ we observe the onset of methyl rotation beginning at 80 K. We believe the higher temperature onset of rotation in 3 is a result of the steric interaction between the methyl group and the *ortho*-proton on the phenyl ring.^59^ Interestingly, we observe a slight recovery of *T*_m_ beginning at 120 K (*T*_m_ = 0.36 μs and 0.55 μs respectively), until 160 K (*T*_m_ = 0.79 μs and 0.75 μs respectively), where relaxation becomes *T*_1_ limited and *T*_m_ decreases with decreasing *T*_1_. We attribute the recovery of *T*_m_ to methyl rotation becoming so rapid, that on the time scale of *T*_m_ for the electron spin, it begins to average out into background nuclear spin noise.^48,53,67^ Though the *T*_m_ of 3′′ is unmeasurable above 280 K (*T*_m_ = 0.44 μs), we were able to measure the *T*_m_ of 3′ up to 300 K (*T*_m_ = 0.43 μs). We also observed power dependent Rabi oscillations at 300 K (Fig. 5) – a hallmark of qubit operation demonstrating the superposition state of 3 can be manipulated at room temperature. Room temperature measurement of *T*_m_ in 3′ makes it one of a paucity of known transition metal coordination complexes with detectable coherence at these temperatures.^13,25,29,40–42,67^

## Conclusions

These results demonstrate a framework to design and modify molecular qubit candidates for high temperature operation. Contrary to the discussion of ligand rigidity in the field previously, we find that π-rigidity provides only small improvements in *T*_1_. Instead, the largest improvements in *T*_1_ come from reduction of OAM through tuning geometry. The molecular geometry determines the energy of the molecular orbitals in the complex, with lower energy excited states reintroducing more OAM *via* the spin–orbit interaction. The extent of recovered OAM controls the strength of the interaction between the spin and the lattice and therefore the relaxation rate. Constraining the primary coordination sphere of the complex reduces the relaxation rate, but the net effect on relaxation is much smaller. While some of this is provided through increased π-rigidity, larger improvements are found from cyclizing the ligand. This implies there may be a wholly unexplored field of macrocyclic qubits that have previously been ignored by the field due to their lack of π-bonding. Design of future qubits for high temperature operation should focus on limiting OAM and SOC as their primary goal. Although 3 is a room temperature qubit candidate its *T*_1_ is still an order of magnitude below other known candidates, some of which display *T*_1_ > 1 μs out to room temperature.^40,80^ We hypothesize the comparatively shorter *T*_1_ of 3 may be the result of small differences in OAM at the metal center, offering a potential handle for future improvements. Synthetic chemistry provides us with the tools to manipulate the ligand field with the incredible precision to create the next generation of qubits based on this theoretical insight.

Notably the systems described herein are primed for integration with future qubit technologies. All three complexes are neutral, allowing for surface assembly *via* monolayer sublimation and potential investigation *via* STM-ESR.^81,82^ The long high temperature coherence times of 3 make it incredibly attractive to interface with a variety of different substrates to investigate the effects surface phonon modes have on relaxing molecular systems.^83^ This study lays the groundwork for these important future directions in the field.

## Data availability

Crystallographic information can be found in the ICSD. The structures of optimized molecules used in density-functional theory calculations can be found at: https://github.com/MTD-group/Molecular_Qubit_Structures

## Author contributions

Conceptualization: M. J. A., J. M. R., and D. E. F.; investigation and formal analysis: M. J. A., K. R. M., M. J. W., D. P. and M. K.W.; resources: L. S. and P. H. O.; visualization: M. J. A. and M. J. W.; supervision: D. E. F. and J. R. M.; all authors contributed to writing the manuscript.

## Conflicts of interest

There are no conflicts to declare.

## Supplementary Material

SC-013-D1SC06130E-s001

SC-013-D1SC06130E-s002
