# Supplementary material for: Chemical control of spin–lattice relaxation to discover a room temperature molecular qubit
Source: Chem Sci. 2022 May 17;13(23):7034–45. doi: 10.1039/d1sc06130e (PMC9200133; doi:10.1039/d1sc06130e)
Supplement: SC-013-D1SC06130E-s001 [file SC-013-D1SC06130E-s001.pdf]

Electronic Supplementary Material (ESI) for Chemical Science.
This journal is © The Royal Society of Chemistry 2022

Supplementary Information:

Chemical Control of Spin-Lattice Relaxation to Create Room Temperature Molecular Qubit Candidates

M. Jeremy Amdur,[1] Kathleen R. Mullin,[2] Michael J. Waters,[2] Danilo Puggioni,[2] Michael K. Wojnar,[1] Mingqiang Gu,[2] Lei Sun,[3] Paul H. Oyala,[4] James M. Rondinelli,[2]* Danna E. Freedman[1,5]*

[1]Department of Chemistry, Massachusetts Institute of Technology, Cambridge, Massachusetts, 02139, United States

[2]Department of Materials Science and Engineering, Northwestern University, Evanston, Illinois, 60208, United States

[3]Center for Nanoscale Materials, Argonne National Laboratory, Argonne, Illinois, 60439, United States

[4]Division of Chemistry and Chemical Engineering, California Institute of Technology, Pasadena, California, 91125, United States

[5]Department of Chemistry, Northwestern University, Evanston, Illinois, 60208, United States

Table of Contents

**Full Experimental Details**	**S4**
**Table S1** | Summary of crystallographic data for **1**	**S14**
**Table S2** | Summary of crystallographic data for **4**	**S15**
**Table S3** | Summary of crystallographic data for **2**	**S16**
**Table S4** | Summary of crystallographic data for **5**	**S17**
**Table S5** | Select structural parameters for **1–3**	**S18**
**Table S6-S7** | Select structural parameters for calculated structures of **1–3** using the B3LYP and PBE functionals	**S19**
**Figure S1** | Compliance ellipsoids for **1–3**	**S20**
**Table S8-S10** | Calculated d-orbital splitting for **1–3**	**S21–S23**
**Figure S2-S4** | Calculated d-orbital splitting for **1–3**	**S21–S23**
**Table S11** | cw-EPR parameters for **1′–3′** and **1″–3″**	**S24**
**Table S12** | Computed cw-EPR parameters for **1–3**	**S24**
**Table S13** | Spin density on the copper center and the ligand in **1–3**	**S24**
**Table S14–S19** | T_1 saturation recovery fit parameters for **1′–3′** and **1″–3″**	**S25−S29**
**Table S20–S25** | T_m 2 pulse Hahn echo decay fit parameters for **1′–3′** and **1″–3″**	**S30−S32**
**Table S26** | Parameters for Debye model fit of T_1 data for **1′–3′** and **1″–3″**	**S33**
**Table S27** | Room Temperature Rabi rate at variable microwave power attenuation for **3′**	**S33**
**Table S28–S33** | Linear and quadratic fits for dg/dq coupling across all vibrational modes	**S34−S39**
**Table S34–S39** | Linear and quadratic fits for dA_{Cu}/dq coupling across all vibrational modes	**S40−S45**
**Table S40–S45** | Linear and quadratic fits for dA_N/dq coupling across all vibrational modes	**S46−S51**
**Figure S5-7** | PXRD for **1–3**	**S52−S54**
**Figure S8** | Visualization of the spin density in **1**	**S55**
**Figure S9** | cw-EPR and simulations for OTP glasses of for **1–3**	**S56**
**Figure S10–S12** | EDFS spectra for **1′–3′** and **1″–3″**	**S57−S59**
**Figure S13–S15** | Saturation recovery and Hahn Echo decay data for **1′–3′** and **1″–3″**	**S60−S62**
**Figure S16–S18** | T_1 and T_m data vs temperature for **1′–3′** and **1″–3″**	**S63−S65**
**Figure S19–S21** | Fits of T_1 data to the Debye model vs temperature for **1′–3′** and **1″–3″**	**S66−S68**
**Figure S22–S24** | Variation in g_x, g_y, and g_z during vibrational motion for highly coupled modes in **1–3**	**S69−S71**
**Figure S25** | Histograms representing the vibrational modes within certain spin-phonon coupling regimes	**S72**
**Figure S26–S27** | Comparison of T_1 obtained from experiment with calculated values	**S73-S74**
**Figure S28** | Cu-E bond length changes during vibrational displacement for **1–3**	**S75**
**Figure S29** | Visualization of the highly coupled low energy vibrational modes in **2**	**S76**
**Figure S30** | Visualization of the modes with the highest SPC coefficient in **1–3**	**S77**
**Figure S31** | UV-Vis spectra of **1–3** in toluene	**S78**
**Figure S32–S34** | IR and Raman Spectra of **1–3**, shown with their calculated vibrational spectra.	**S79−S81**
**Figure S35–S37** | Nutation experiments for **1′–3′** at 5 K.	**S82−S83**
**Figure S38** | Visualization of the 70% spin density in **1–3**.	**S84**
**References**	**S85**

Full Experimental Details

General considerations. Manipulations and syntheses of air sensitive complexes were performed under a N_2 atmosphere with either an MBraun Unilab Pro glovebox, Vacuum Atmosphere Nexus II glovebox, or using Schlenk techniques. Glassware was either oven-dried at 150 °C for at least four hours and/or flame-dried prior to use. Tetrahydrofuran (THF), *n*-hexanes, diethylether (Et_2O), and dichloromethane (DCM) were dried using a commercial solvent purification system from Pure Process Technology and stored over 3 or 4 Å sieves prior to use. Et_2O was subjected to a test with a standard purple solution of sodium benzophenone ketyl in THF to confirm low O_2 and H_2O content prior to use. Bis(acetylacetone) ethylene diamine (acacen), N,N′-dimethyl-4-amino-3-penten-2-imine (Me_2Nac)[1], copper tetramethyltetraazaannulene (Cu(tmtaa), **3**)[2] and nickel tetramethyltetraazaannulene (Ni(tmtaa), **6**)[2] were synthesized by literature methods. All other reagents were used as received.

Cu[$(CH_3)_2C_5H_7N_2$)]$_2$ Cu(Me_2Nac)$_2$ (1). A 25 mL Schlenk flask with charged with a magnetic stirbar, 5 mL of THF, and Me_2Nac (0.130 g, 1.03 mmol). The solution was cooled to 0 °C and ^nBuLi was added with stirring. A separate 25 mL Schlenk flask was charged with $CuCl_2$ (0.067 g, 0.52 mmol) and 10 mL THF and cooled to −78 °C. After 30 minutes of stirring, the lithiated Me_2Nac solution was added to the $CuCl_2$ solution under N_2. The solution was slowly warmed to room temperature overnight. The solvent was then evaporated and replaced with *n*-hexanes. The deep purple solution was filtered through alumina, concentrated, and left at −35 °C overnight. Decanting off the solvent and drying afforded pure, dark crystals of **1** (120 mg, 38% yield) that were suitable for single crystal X-ray diffraction. FTIR (cm^{-1}): 444.7, 714.3, 998.4, 1023.1, 1060.2, 1134.3, 1231, 1231, 1249.6, 1290.7, 1354.6, 1371, 1424.6, 1424.6, 1443.1, 1463.7, 1523.4, 1552.2. UV-Vis (toluene) (λ (nm), ε (mM^{-1} cm^{-1})): (315, 8.3) (353, 8.0) (415, 8.6) (510, 0.8). ESI-MS (*m/z*): $[M]^+$ calcd. for $C_{14}H_{27}N_4Cu$, 313.14; found 314.15)

Zn[$(CH_3)_2C_5H_7N_2$)]$_2$ Zn(Me_2Nac)$_2$ (4). A 25 mL Schlenk flask was charged with a magnetic stirbar, 5 mL of THF, and Me_2Nac (0.130 g, 1.03 mmol). The solution was cooled to 0 °C and ^nBuLi was added with stirring. A separate 25 mL Schlenk flask was charged with $ZnCl_2$ (0.068 g, 0.52 mmol) and 10mL THF and cooled to −78 °C. After 30 minutes of stirring, the lithiated Me_2Nac solution was added to the $CuCl_2$ solution under N_2. The solution was slowly warmed to room temperature overnight. The solvent was then evaporated and replaced with *n*-hexanes. The clear solution was filtered through alumina, concentrated, and left at −35 °C overnight. The solvent was then removed to afford translucent crystals. To remove unreacted Me_2Nac, these crystals were redissolved in *n*-hexanes and the crystallization procedure was repeated. Decanting off the solvent and drying afforded pure, pale crystals of **4** (110 mg, 35%) that were suitable for single crystal X-ray diffraction. ^{1}HNMR (400 MHz, $CDCl_3$): δ 4.30 (s, 2H), 2.95 (s, 6H), 1.93 (s 6H). FTIR: UV-Vis (toluene) (λ (nm), ε (mM^{-1} cm^{-1})): (418, 1.30). IR: 631.7, 667.4, 679.9, 713.7, 842.9, 997.2, 1012.6, 1022.3, 1066.6, 1138.0, 1357.9, 1372.4, 1409.0, 1430.2, 1481.3, 2336.8, 2361.8, 2911.5, 2973.3, 2982.0 ESI-MS (*m/z*): $[M]^+$ calcd. for $C_{14}H_{27}N_4Zn$, 314.15; found 314.20).

Cu[$C_{12}H_{18}N_2O_2$] Cu(acacen) (2). The acacen ligand (1.0 g, 4.4 mmol) and excess potassium carbonate (2.0 g, 14.8 mmol) were dissolved in 50 mL of H_2O in a 250 mL round bottom flask and heated to reflux with stirring. To this solution was added a solution of $CuCl_2$ (0.78 g, 4.4 mmol) in 15 mL of H_2O which immediately precipitated a purple solid. The solution was stirred at reflux for 1 hour, allowed to cool, and then filtered. The solid was redissolved in DCM and filtered through a silica plug. The solvent was then

evaporated to afford **2** (1.0 g, 81%). Crystals suitable for single crystal X-ray diffraction were obtained by layering water over an acetone solution of **2**. FTIR (cm^{-1}):455, 648.5, 737, 751.3, 944.9, 1012.8, 1039.6,1274.3, 1282.5, 1352.5, 1416.3, 1516.3, 1476, 1502.8, 1513, 1589.2. UV-Vis (toluene) (λ (nm), ε (mM^{-1} cm^{-1})): (538, 0.6). ESI-MS (*m/z*): $[M\text{-}H]^+$ calcd. for $C_{12}H_{19}N_2O_2Cu$, 286.07; found 286.06)

Ni[$C_{12}H_{18}N_2O_2$] Ni(acacen) (5). The acacen ligand (1.0 g, 4.4 mmol) and excess potassium carbonate (2.0 g, 14.8 mmol) were dissolved in 50 mL of H_2O in a 250 mL round bottom flask and heated to reflux with stirring. To this solution was added a solution of $NiCl_2$ (0.95 g, 4.4 mmol) in 15 mL of H_2O which immediately precipitated an orange solid. The solution was stirred at reflux for 1 hour, allowed to cool, and then filtered. The solid was redissolved in 25 mL of DCM and filtered through a silica plug. The solvent was then evaporated to afford **5** (1.0 g, 81%). Crystals suitable for single crystal X-ray diffraction were obtained by layering water over an acetone solution of **5**. ^{1}HNMR (400 MHz, $CDCl_3$): δ 4.91 (s 2H), 2.03 (s, 6H), 1.88 (s, 6H). FTIR (cm^{-1}): 614.3, 691.4, 766.7, 815.9, 950.0, 1016.5, 1048.3, 1073.3, 1106.2, 1126.4, 1220.0, 1238.6, 1402.3, 1506.4, 1587.4. UV-Vis (toluene) (λ (nm), ε (mM^{-1} cm^{-1})): (538, 0.6). ESI-MS (m/z): $[M\text{-}H]^+$ calcd. for $C_{12}H_{19}N_2O_2Ni$, 281.08; found 281.02)

Preparation of diluted powder EPR Samples: EPR samples **2′** and **3′** were prepared through dissolution of the paramagnetic center and its diamagnetic analogue in a 1:100 mass ratio (**2**:**5**, **3**:**6**) in acetone. Then, with aggressive stirring, this solution was added to water to precipitate a mixed powder approximately 1% paramagnetic center by mass. For **1′**, this approach did not succeed, so diluted powders were instead prepared by taking a concentrated solution of 1:100 **1** to **4** in *n*-hexanes, and leaving it at −35 °C overnight. This yielded pale pink mixed single crystals. These crystals were then recovered, thoroughly crushed, and then dried to yield the dilute mixed powder of **1′**.

Preparation of glass phase measurements with o-terphenyl. This study focused on manipulation of the local molecular phonons (vibrations). However, lattice phonons are important in determining the relaxation dynamics of spin systems. To ensure that subtle differences in lattice modes did not impact our results, we needed to compare relaxation dynamics in an identical glassing solvent matrix as well. When all three complexes are dissolved in the same solution, they couple to the same lattice modes, ensuring any differences in observed dynamics are due to differences in the molecules themselves. Many conventional glassing solvents either melt well below room temperature (toluene, 2-methyltetrahydrofuran), or are too polar to solubilize the complexes investigated in this report. Ortho-terphenyl (OTP) overcomes these challenges. OTP is a solid at room temperature with a melting temperature of 60 °C and a glass transition temperature (T_g) of −30 °C.[3] We prepared 1% by weight solutions of each of the complexes in OTP (hereafter referred to as **1″**–**3″**) and 0.1% by dilutions (hereafter referred to as **1‴**–**3‴**), and show values for the dynamics of these solutions in the SI as well. OTP glasses are not stable for long periods of time. Between 12 hours and 4 days later, the glasses would eventually form crystallites. For compounds with many aromatic groups (such as **3**), we observed no change in cw-EPR spectra before and after the glass crystalized, likely due to retention of homogenous mixing after crystallization. For compounds with a non-zero dipole moment across the molecule (**2**) although no features within the cw-EPR spectra changed during crystallization of OTP, signal intensity decreased drastically, to the point where no detectable signal remained after 5 days. Upon remelting and refreezing of the sample, signal intensity returned to previous values. To minimize any effects of OTP crystallization on measurements, we glassed all solutions in OTP immediately prior to measurement. Occasionally, we observed orientation effects in glassed systems of **3**,

suppressing certain transitions and increasing the intensity of others. On those occasions, the glasses were remade prior to measurement. This preferred orientation was infrequently observed.

For all three systems, T_1 was shorter in the glassy matrix than in the solid state at 5 K (5 ms in **1′** compared to 3 ms in **1″**, 14 ms in **2′** compared to 7 ms in **2″**, and 11 ms in **3′** compared to 1 ms in **3″**). However, these differences disappeared at higher temperatures. In the regime where relaxation was dominated by local mode relaxation, T_1 was largely independent of the matrix. This highlights an important facet of spin-lattice relaxation in $S = 1/2$ first row transition metal systems: low temperature dynamics are dominated by lattice phonons whereas high temperature dynamics are dominated by intrinsic molecular properties. At low temperatures, therefore, the unique phonon densities of different matrices can have large impacts on relaxation behavior. At high temperatures, local mode mediated relaxation dominates, and dynamics can be largely considered matrix agnostic. T_m increases in OTP solutions due to the low nuclear spin density of OTP.[4] This effect is most strongly observed in **1**, where removing the methyl group rich environment of **4** enhances T_m by nearly 2 orders of magnitude (at 5 K, T_m = 0.32 μs in **1′** compared to T_m = 14 μs in **1″**).

We observe the same trends observed in the relaxation dynamics of **1′**–**3′** in **1″**–**3″** (**Table S7**). However, there are some slight changes in magnitude of the relaxation parameters. A_{Dir} increases in OTP, which is expected from the shorter T_1 in the glass. While we observe a change in B_{Ram}, it is important to note that B_{Ram} can be selected somewhat arbitrarily for a given value of Θ_D.[5] Since the Θ_D of the OTP glass was assigned an arbitrary value of 65 K for all three fits, whereas Θ_D in the solid state systems was a fit parameter unique to each system, changes in B_{Ram} could be attributed to changes in Θ_D that we cannot model. Deviations in C_{Loc} and Δ_{Loc} are small and frequently within the error of the fit, further reinforcing that local mode relaxation is matrix agnostic.

Electron Paramagnetic Resonance Measurements. Samples were prepared for analysis by a solid-state dilution in their respective diamagnetic analogues. Solid-state dilutions were prepared in a 1:100 (1%) ratio to suppress the influence of intermolecular electronic spin interactions on T_m and T_1. All samples were loaded into 4 mm OD quartz tubes (Wilmad 707-SQ-250M), restrained with eicosane, and flame-sealed under high vacuum. Solution phase measurements were prepared in a 1% by weight dilution with OTP. Though this leads to varying spin concentrations of spin/unit volume, the variation in terms is small (between 1 and 4% spin/volume). The effects of these concentration differences are minimal, and our T_m times are more heavily influenced by the nuclear spin concentration of the matrix (as discussed above). Additionally, the effects of cross relaxation are naturally suppressed at the higher temperatures of interest in this study (70 K).[6]

Spectroscopic data were obtained on either a Bruker E580 X/Q-band spectrometer or a Bruker E560 X-band spectrometer, both with a split ring resonator (ER4118X-MS5) and a 1 kW TWT amplifier (Applied Systems Engineering). Prior to all pulsed measurements, the resonator was over-coupled to minimize ringdown following application of the microwave pulses. Temperature was controlled with an Oxford Instruments CF935 helium cryostat and an Oxford Instruments ITC503 temperature controller. All EPR data were processed by a combination of XEpr (data collection),[7] Matlab (phasing and normalization of EPR data),[8] Easyspin (modeling and simulation of cw-EPR spectra),[9] and Origin (fitting of decay curves).[10] Absolute intensities of the cw-EPR spectra were normalized between 0 and 1 then, simulated using the pepper function in EasySpin.

Spin-lattice relaxation times were obtained using saturation recovery sequences at the highest intensity peaks in the echo-detected spectrum. These sequences achieved saturation by applying a picket-fence

saturation sequence of twenty consecutive 12 ns pulses. Following a delay time T beginning at 100 ns, Hahn echo detection was used to monitor the recovery from saturation with $\pi/2$ and π pulses of 16 and 32 ns, respectively. Four-step phase cycling was used on these measurements. All data was phased by maximization of the sum of the square of the data in the real component of the spectrum. For **2′–3′** and **1″–3″**, data was collected with logarithmic spacing, allowing for data to quickly be taken quickly across orders of magnitude change in time. The T_1 of **1′** was collected using a slightly different detection scheme wherein data was collected with linear spacing, allowing us to capture more points in the regions of largest change. The best fit was obtained using a monoexponential fitting function[11] using the following equation, $I = -A(e^{-\left(\frac{t}{T_1}\right)} - I_0 - 1)$. The fit to low temperature data could be improved by including a stretch factor (σ) to account for the influence of multiple processes with slightly different T_1 constants using the equation $I = -A(e^{-\left(\frac{t}{T_1}\right)^{\sigma}} - I_0 - 1)$. This distribution of T_1 relaxation times has been attributed to a handful of factors, most likely in this report are inequivalences between magnetic cites, causing slight deviation in relaxation rates.[12] A Stretch factor was often required to adequately fit low temperature data. At higher temperatures where single phonon relaxation becomes less prominent, these inequivalences matter less and the stretch factor trends towards 1 as shown in **Tables S14–S19**.

The temperature dependence of T_1 was fit to account for the direct process, Raman process, and local modes using the following expression,

$$\frac{1}{T_1} = A_{Dir}T + B_{Ram}\left(\frac{T}{\Theta_D}\right)^9 J_8\left(\frac{\Theta_D}{T}\right) + C_{Loc}\frac{e^{\Delta_{Loc}/T}}{(e^{\Delta_{Loc}/T} - 1)^2}$$

Where T is temperature, A_{Dir} is the coefficient for the direct process, B_{Ram} is the coefficient for the Raman process, J_8 is the transport integral,[13] Θ_D is the Debye temperature, C_{Loc} is the coefficient for local modes, and Δ_{loc} is the energy of the active local modes of vibration. See below for further discussion of the fitting process of the temperature dependence of T_1 across **1′–3′** and **1″–3″**.

Phase memory times (T_m) were obtained using a two-pulse Hahn echo sequence, $\pi/2$-τ-π-τ-*echo*, where τ is the time delay between pulses, and $\pi/2$ or π denote microwave pulses, 16 ns and 32 ns, respectively. Starting delay times were selected to minimize the effects of any observed ringdown. These delays ranged from 100 to 200 ns for all complexes. Four step phase cycling was employed for these experiments as well. Most systems were modeled using a simple monoexponential decay function $I = Ae^{\left(\frac{2\tau}{T_2}\right)} - I_0$. Some systems also exhibited electron spin echo envelope modulation (ESEEM) of the echo intensity, primarily from weakly coupled ^{1}H nuclei. Due to the inherent errors in adding the necessary fit parameters to the relaxation equation to model ESEEM, we chose to ignore the ESEEM in our fits.

Nutation experiments were performed by applying a tipping pulse (τ_p) to generate a superposition state, followed by followed by a Hahn-echo detection sequence (τ_p-T-$\pi/2$-τ-π-τ-*echo*). For constant τ and microwave power, the echo intensity varies with the length of the tipping pulse. As τ_p is incremented, the spin samples all superposition states, resulting in a sinusoidal oscillation of echo intensity. Plotting the oscillation in echo intensity versus the tipping pulse length gives a decaying sinusoidal curve, known as a Rabi oscillation – the hallmark of qubit viability.[14,15] For our experiments, τ_p was incremented in 2 ns steps

with T = 340 ns, and $\pi/2$ and π pulse lengths of 16 ns and 32 ns respectively. For the room temperature measurements of **3′**, a large baseline needed to be subtracted from the data. This baseline was only present for these high temperature measurements, suggesting it results from the weak echo intensity.

The Fourier transform of nutation data yields plots of the frequency of oscillation in the experiment. At low temperatures, we observe a secondary peak at approximately 14 MHz, the Larmor frequency of the nearby proton nuclei, as a result of the Hartman-Hahn effect.[16] To demonstrate room temperature qubit manipulation of **3′**, we measured the rate of Rabi oscillations (known as the Rabi rate) at three microwave powers. The relative microwave power was calculated as:

$$\text{relative power} = \sqrt{10^{-0.1(A)} / 10^{-0.1(Z)}}$$

where A is the microwave power attenuation and Z is the lowest microwave power attenuation in the set of three molecules examined in the experiment (7.2 dB). Although the trend between Rabi rate and relative microwave power attenuation is expected to be linear, we observe a small deviation from linearity (R^2 = 0.988). We attribute this to the weak echo intensity of **3′** at low microwave powers. Because the echo intensity is so weak, we are only able to observe one period of the oscillation, leading to error in our estimation of its Rabi rate.

X-ray Structure Determination. All diffraction data were collected in the X-ray crystallography lab of the Integrated Molecular Structure Education and Research Center (IMSERC) at Northwestern University. Single crystals of **1**, **2**, **4**, and **5** suitable for X-ray diffraction analysis were coated in Paratone N oil and mounted on a MiTeGen MicroLoopTM. Crystallographic data for **1** and **2** were collected on a Rigaku diffractometer equipped with a MoKα sealed tube diffraction source with a graphite monochromator, and a Bruker APEX II detector. Data for **4** and **5** were acquired on a Rigaku XtaLab Synergy, single source at home/near, HyPix diffractometer equipped with a MoKα PhotonJet X-ray source. All datasets were collected at 100 K. Raw data were integrated and corrected for Lorentz and polarization effects with SAINT v8.27B.10 (for **1** and **2**) or CrysAlisPro 1.171.40.53 (for **4** and **5**)[17] Absorption corrections were applied using SADABS.[18] Space group assignments were determined by examination of systematic absences, E-statistics, and successive refinement of the structures. Structures were solved using direct methods in SHELXT and further refined with SHELXL-2013[19] operated with the OLEX2 interface.[20] Hydrogen atoms were placed in ideal positions and refined using a riding model for all structures. Structural parameters for **1**–**2** were obtained from single crystal X-ray diffraction experiments. (**Table S1**–**2**).Structural parameters for **3** were obtained from the literature reported crystal structure.[21] To ensure crystallographic similarity, powder X-ray diffraction samples of **3** were compared to simulated powder X-ray diffraction patterns obtained from Mercury with a full-width half-max of 0.5° 2θ. A well-ground powder sample of **3** was sandwiched between pieces of Kapton tape. Powder X-ray diffraction patterns were collected on a STOE STADI MP diffractometer equipped with CuKα radiation.

The provided crystal structure of **1** shows a positional disorder of the complex in the unit cell with approximately 90% of the complexes of one orientation, and 10% with the antiparallel orientation. Solving the structure without the inclusion of this second orientation leads to a significant amount of unmodeled electron density, but because of the low occupation, there is significant disorder in modeling this orientation, resulting in an A-level and B-level alert. In **2**, there is a difficult to model, disordered, partially occupied water molecule in the crystal structure. The exact orientation of the oxygen atom causes a B-level alert, likely due to occupational disorder. In **2**, additional alerts were caused by the size of the crystal. Due

to the thinness of the crystal, we decided not to cut it in order to maximize our sample exposure to X-rays. This did however lead to a larger than ideal crystal, causing an A-level alert.

The reported structure of **3** was taken at room temperature (298 K) and is being compared to structures at 100 K. Thought this temperature difference does make exact comparison of bond lengths difficult – our assignment of relative τ_4' values are validated by comparison of the computed relaxed geometries (**Table S20**).

To confirm our determined crystal structure, we performed powder X-ray diffraction experiments on **1**–**3** at room temperature with CuKα (1.54065 Å) radiation (**Figure S5**–**S7**). The resulting diffraction patterns were compared to the predicted spectra from their single crystal structures. There is a slight shift in peak 2θ present in **1**–**2,** resulting from comparing powder patterns obtained at 300 K to crystal structures obtained at 100 K.

Other Physical Measurements. Infrared spectra were recorded on a Bruker Alpha FTIR spectrometer equipped with an attenuated total reflectance accessory and diamond anvil. Electrospray ionization mass spectrometry measurements were performed on MeOH/DCM solutions of **1**–**6** with Bruker AmaZon SL ESI-Ion Trap Mass Spectrometers at the IMSERC facility of Northwestern. UV-Vis spectra were collected on toluene solutions of **1**–**6** with a Varian Cary 5000 spectrophotometer.

Discussion of sources of error in fitting variable temperature T_1. In modeling these data, we focused on the direct process, the Raman process, and the local mode process. As all complexes are monometallic and S = ½, an Orbach mechanism, which necessitates accessible low-lying spin states only available in higher spin systems or exchange coupled systems with multiple spin centers, is physically unreasonable and would lead to overparameterization.[22,23] Because the model used to fit the data (The Debye model) was derived for extended solid systems of much higher crystallographic symmetry (the highest symmetry examined in this report is orthorhombic whereas the Debye model was developed for cubic systems) there is a large degree of inherent error associated with the fit parameters for these systems.[23,24] The Debye temperature obtained from these fits is not an analogous parameter to the Debye temperature of ideal solids. In these systems, it is far better understood as metric representing the phonon energy of an individual lattice. Additionally, the Debye temperature and Raman coefficient B_{Ram} are heavily interdependent. It has been demonstrated that a change in B_{Ram} of approximately one order of magnitude can be offset by a change in the Debye temperature by approximately 30%.[5] This is typically corrected for by limiting the range of acceptable Debye temperatures to a range typically associated with molecular solids (between 50 K – and 120 K).[22,24] We use this to our advantage in fitting the OTP data to hold lattice phonon contributions constant. We first fit the data for **3″** with no parameters held constant, and with the aforementioned constraint on Θ_{D}. This led to a fit Θ_{D} of 65 K. We then used this as a representative Θ_{D} for the OTP matrix, refit the data for **3″** with Θ_{D} now held constant at 65 K, as well as fitting the T_1 data for **1″**–**2″** as well.

Additionally, significant errors are associated with the direct process, because this mechanism is operative at the lowest temperatures of measurements and is typically fit to only the few data points below 10 K. The Δ_{Loc} parameter heavily discussed in the main text represents some weighted average of all local modes contributing to spin-lattice relaxation. By the strictest definition, the term represents a single mode in the system. However, as we and others demonstrate, modeling these systems with only a single impactful local

mode is not accurate.[25] Technically, one could add additional $C_{Loc}\frac{e^{\Delta_{Loc}/T}}{\left(e^{\Delta_{Loc}/T}-1)\right)^2}$ terms – one for each mode being modeled.[26] However, adding additional terms to the Debye model leads to problematic overparameterization.[27] Therefore, convention is to fit relaxation to a single Δ_{loc} term, with the caveat that it is likely not representative of a true mode in the system, and instead gives information about the average of all modes in the system. As we demonstrate in the main text, this method does give partial information about vibrational modes responsible for relaxation, but does not paint a complete picture of the local modes process in molecular qubit candidates.

Modeling of cw-EPR Spectra cw-EPR spectra were modeled through the program EasySpin, run through MatLab.[8] In all systems, anisotropy in the *g*-tensors and hyperfine interaction (*A*-tensors) were considered. The hyperfine interaction is strongly distance dependence, and is generally only significant for the primary coordination sphere in transition metal systems. Therefore, the only atoms treated as having measurable hyperfine interactions in our model were the metal center and the nuclear spin bearing atoms of the primary coordination sphere (nitrogen in all systems). In an attempt to further reduce spin-spin broadening, we performed cw-EPR on 0.1 % by weight solutions of **1**–**3** in OTP (**1′′′**–**3′′′**). Linewidths remained largely consistent with the higher electronic spin concentration **1′**–**3′**, suggesting cw-EPR linewidths are not limited by interelectronic interactions, and are instead limited by interactions with nearby nuclear spins.

Both **1** and **3** were modeled with an anisotropic line broadening term. Easyspin supports modeling anisotropic strain with several different methods. To reduce the parameter space, we chose to model the systems with only a single source of line broadening. It should be noted, however, that likely a combination of many sources of anisotropy contribute to line broadening in each system. For **1′** and **1′′′**, the most success was found using an "H-strain" term, an anisotropic smearing of magnetic parameters within the system causing broadening of the observed transitions. The choice to model with H-strain was made as there is no clear chemical origin of the anisotropy.

3′ was best modeled with a "*g*-strain" parameter, which models an anisotropic distribution of *g* tensors existing in the crystal causing an anisotropic broadening of transitions. In the case of **3′**, the use of *g*-strain makes particular sense as, in the crystal structure of both **3** and **6**, the molecule occupies two unique positions in the solid-state crystal. In **3′**, **3** can occupy either of the two sites, and therefore has a cw-EPR spectra composited of 2 different molecules, with similar but distinct magnetic parameters. It is very likely that an even more accurate simulation could be made from specifically modeling two unique, uncoupled copper sites in the system. The existence of this broadening is highlighted by the significant decrease in anisotropic broadening in **3′′′**, wherein the large "smear" of transitions around 340 mT is resolved into several distinguishable peaks. In **3′′′**, the system could now be modeled with an isotropic Lorentzian.

Although the crystal structure of **2** also has two unique sites in the crystal, **2′** is well modeled with an isotropic Lorentzian. This is best understood as an effect of the different matrices the spins are in. Both **3** and **6** crystalize into the $P2_1/c$ space group with two unique molecule sites per unit cell.[21,28] Although **2** and **5** both crystalize into the $C2/c$ space group, the unit cell of **5** has one molecular site in the cell.. When **2** is diluted into **5** to make **2′**, it substitutes into the crystal structure of **5**, and there is only 1 magnetic site the molecule can occupy. Therefore, strain effects are not observed, and we obtained the well resolved cw-EPR spectra reported in the main text (**Figure 2**). **2′′′** was similarly modeled with a simple isotropic Lorentzian.

Notes on Comparing Geometries of Different Complexes The dihedral angle cited in the main text and in figures was defined as follows: A single subunit is defined as a 5-carbon chain with a main group donor atom located on the 2 and 4 carbons. In all three complexes, a plane was defined as the plane created by the two donor atoms on a NacNac or NacAc subunit and the metal center. Since each complex contained two subunits, the dihedral angle discussed in the paper is the angle between these two intersecting planes. As one would expect, it is large for a tetrahedral or pseudo tetrahedral molecule, and small for a square planar molecule.

There exist 2 common parameters which compare the geometry of four coordinate molecules to rank how similar they are to those geometric extremes – τ_4 and τ_4', defined as follows:

$$\tau_4 = -0.00709\alpha - 0.00709\beta + 2.55$$
$$\tau_4' = -0.00399\alpha - 0.01019\beta + 2.55$$

Where α and β are the two largest valence angles in the complex.[29,30] Although the parameters are similar at the extreme (0 being perfectly square planar, 1 being perfectly tetrahedral), τ_4' uniquely identifies the α and β angles of the complex, and therefore better highlights the unique distortions between structures.

Computational Details Vibrational modes, stiffness, and spin density for each of the molecules was calculated using the Γ-point version of VASP[31–33] with the Perdew–Burke-Ernzerhof (PBE) functional. A plane wave cutoff of 600 eV, an augmentation grid plane wave cutoff of 2400 eV, and an energy convergence of 10^{-8} eV was used for all VASP calculations. Molecular structures were extracted from experimental crystal structures, giving a gas phase approximation. The structures were relaxed with a force convergence of 5×10^{-5} meV/Å and a minimum of 10 Å gap between the molecule and its periodic images was used. The vibrational modes were calculated from a dynamical matrix populated using finite displacements of 0.01 Å of each atom. The vibrational density of states was calculated via a gaussian folding method with a width of 4 cm^{-1}.[34] The intensities of each mode were normalized to one. Eigenvalues of each mode were projected onto individual atoms and then summed by atom type to give the full atom projected density of states.

Local stiffness of the Cu atoms was found by fitting the local potential energy U, with the equation:

$$U = U_0 + \frac{1}{2}(\Delta r)^T H(\Delta r) \quad (1)$$

Where U_0 is reference potential energy, Δr is the finite displacement vector from the equilibrium position, and H is the matrix of second order polynomial terms approximating the Hessian matrix. Finite displacements were evaluated on a 5 x 5 x 5 grid around the equilibrium position in increments of 0.01 Å. Spin density is shown with an isosurface containing 70 percent of the density.

The structures were re-relaxed with ORCA[35] version 4.0.1.2 using the B3LYP functional[36–39] and the def2-TZVP[40] basis set with the def2-/JK[41] auxiliary basis set. Hyperfine coupling and g-tensor calculations were performed on these re-relaxed structures using the ORCA program. All *g*-tensor calculations used the same functional and basis set as the relaxations. For hyperfine coupling the functional and basis set remained the same as other ORCA calculations with the exception that the triply polarized basis set CP(PPP)[12] was used for the Cu atom. For all ORCA calculations, tight SCF convergence criteria (giving an energy change of 2.7211×10^{-7} eV) and DFT integration grid 6 (Lebedev 590 points for the angular grid and

the IntAcc parameter set to 5.34 for the radial grid) were used. A tighter DFT integration, grid 7, was used around the Cu atom (Lebedev 770 points for the angular grid and the IntAcc parameter set to 5.67 for the radial grid). These setting allowed for calculation of g-tensor values that were converged to $1x10^{-4}$. The g-tensor and hyperfine couplings to the vibrational modes were calculated for each normal mode with five amplitudes, four between 0.02 and 0.08 Å. One additional amplitude at –0.08 Å was also computed to indicate if the changes in the g-tensor with respect to mode amplitude were even or odd for a given mode. All g-tensor data for distorted structures was fit to a linear model and a quadratic model to determine the best fit for each mode. For the linear model the data was fit to **Equation 2**, where g_{eq} is the equilibrium g-tensor value, $d_{amplitude}$ is the amplitude of the distortion applied to the equilibrium structure, and g_{dis} is the g-tensor value for the distorted structure.

$$g_{dis} = (b * d_{amplitude}) + g_{eq} \qquad (2)$$

For the quadratic model a similar method was used with **Equation 3**.

$$g_{dis} = (a * d_{amplitude}{}^{2}) + g_{eq} \qquad (3)$$

The calculated g-tensors for each distorted structure showed that modes with significant coupling, greater than 10^{-3} Å^{-1}, were well described by a linear approximation with most modes having an r^2 value above 0.95 (**Table 27-S29**). Several of these strongly coupled modes for **1** show a "V-shaped" behavior and are identified in **Table S27**. Modes that were well described by a quadratic approximation all have coupling constants below 10^{-3} Å^{-1}. Occasionally a mode could not be fit to a quadratic approximation due to the value being constant with respect to displacement. Displacements where this occurs are notated with "nan" in **Table S27–S44**. The linear approximation of the first derivative of the g-tensor with respect to the phonon modes, was then used the calculate a weighted spin phonon coupling (**Equation 4**)

$$V_{sph}^{2} = \sum_{Q=0}^{Q=n}\left(\left(\frac{\partial g}{\partial Q}\right)^{2}\int_{Tmin}^{Tmax}\frac{1}{e^{\frac{\omega_Q}{k_bT}}-1}dT\right) \qquad (4)$$

Where ω, is the frequency of the mode and k_b is Boltzmann's constant. Temperature, T, ranges from 0–300 K and Q ranges from 0 to n where n is the number of real modes in the frequency range 0–500 cm^{-1}. This range was chosen as representative of all modes with a realistic thermal occupancy.

To analyze the perturbation of the primary coordination sphere during relaxation, we applied a bond distance that computes the average distance between the copper cation (C) and every nitrogen donor (N) with respect to each axis (**Equation 5**).

$$d_{Cu-N} = \frac{\sum_{k=1}^{4}\left(\sum_{i=1}^{3}(C_i - N_{k_i})^2\right)^{\frac{1}{2}}}{4} \qquad (5)$$

This metric was calculated for every distorted structure and then fit to a linear model with the distortion amplitude to determine the change in the bond length metric with respect to distortion amplitude. Larger $d_{Cu\text{–}N}$ values indicate more distortion in the primary coordination sphere along a vibrational mode. For **2**, this metric was altered slightly to include the perturbation in the donor oxygen atoms as well. The description and calculation are identical, treating oxygen displacement identically to nitrogen displacement. To identify the unique environment, the parameters was termed $d_{Cu\text{–}E}$.

A simple thermal model based off the work of Kazmierczak et al[42] was used to calculate T_1 for all three molecules. The model weights the SPC coefficients by their Bose-Einstein occupancy and then is normalized with experimental T_1 data for Cu(tmtaa) in o-Terphenyl (eq. 6). A constant normalization factor (a) was determined from the data at 160K.

$$T_1 = \frac{a}{\sum_{Q=0}^{N} \left(\frac{\partial g}{\partial Q}\right)^2 \frac{e^{\frac{w_Q}{k_b T}}}{\left(e^{\frac{w_Q}{k_b T}-1}\right)^2}} \quad (\mathbf{6})$$

$$a = \frac{SPC_{weighted}(160\ K)}{T_{1,exp}(160\ K)}$$

Comparison of PDOS with Experimental Raman and IR Spectra: The gas phase approximation was used for phonon calculations. This has been demonstrated to cause discrepancies at very low frequencies due to the lack of lattice modes present in the approximation.[27]

Direct comparison of predicted vibrational behavior to experimental results is difficult. While calculations necessitate ignoring the local matrix environment to prevent the exponential scaling of computational difficulty, these factors necessarily impact vibrational modes, both in energy and in oscillator strength. As such, we wish to stress that our results from DFT calculations cannot be 1:1 mapped onto experimental results in real matrices. For example, the vibrational modes predicted at 129 cm^{-1} for **2** that is heavily discussed in the main text, may be shifted by tens of wavenumbers in energy in a real matrix, and these shifts could also shift the predicted spin-phonon coupling of those vibrational terms. As such, we only use our model to guide our understanding of experimental results, and stress that a direct comparison of experimental and theoretical vibrational energies is non-trivial.

Despite these approximations, most Raman modes match closely with a computed mode, giving an overall low root mean square error of 19.73, 17.13, and 11.74 cm^{-1} for **1–3** respectively. This error is similar to that found in other four coordinate copper complexes.[43]

Table S1 | Crystallographic information for the structural refinement of **1**.

Empirical Formula	$C_{12}H_{26}N_4Cu$
Formula weight	313.93
Temperature	101.0(2)K
Wavelength	0.71073 Å
Crystal System	Orthorhombic
Space Group	*Pbcn*
Unit Cell Dimensions	a = 11.819(2) Å, α = 90°
	b = 12.632(2) Å, β = 90°
	c = 10.1183(16)) Å, γ = 90°
Volume	1510.7(4) $Å^3$
Z	4
Density (calculated)	1.493 Mg/m^3
Absorption coefficient	1.436 mm^{-1}
F_{000}	732.0
Crystal color	Purple
Crystal size	0.378 × 0.133 × 0.061 mm^3
θ range	4.018 to 48.464°
Index ranges	$-19 \leq h \leq 19$
	$-20 \leq k \leq 20$
	$-6 \leq l \leq 16$
Reflections collected	45870
Independent reflections	3754 [R_{int} = 0.0461]
Completeness to θ = 52.48°	100 %
Absorption correction	Multi-scan
Maximum and minimum transmission	0.655 and 0.745
Refinement method	Full-matrix least-squares on F^2
Data / restraints / parameters	3754 / 0 / 191
Goodness-of-fit on F^{2a}	1.038
Final R indices [I > 2σ(I) = 17728 data]^b	R_1 = 3.11 %, wR_2 = 7.29 %
R indices (all data, 0.80 Å)	R_1 = 4.97 %, wR_2 = 8.04 %
Largest diff. peak and hole	0.57 and –0.51 e/$Å^{-3}$

^{a} GooF = $[\Sigma[w(F_o^2-F_c^2)^2] / (n-p)]^{1/2}$ where n is the number of reflections and p is the total number of parameters refined. $^bR_1 = \Sigma||F_o|-|F_c|| / \Sigma|F_o|$; $wR_2 = [\Sigma[w(F_o^2-F_c^2)^2] / \Sigma[w(F_o^2)^2]]^{1/2}$

Table S2 | Crystallographic information for the structural refinement of **2**.

Empirical Formula	$C_{12}H_{18}N_2O_2Cu{\cdot}0.25(H_2O)$
Formula weight	285.82 g/mol
Temperature	100.0(1) K
Wavelength	0.71073 Å
Crystal System	Monoclinic
Space Group	*C*2/*c*
Unit Cell Dimensions	a = 34.5263(12) Å, α = 90.0°
	b = 7.5707 Å, β = 107.444(2)°
	c = 19.7874(7) Å, γ = 90.0°
Volume	4935.3(3) Å^3
Z	16
Density (calculated)	1.545 Mg/m^3
Absorption coefficient	1.762 mm^{-1}
F_{000}	2395.0
Crystal color	Purple Red
Crystal size	1.478 × 0.179 × 0.103 mm^3
θ range	2.472 to 56.792°
Index ranges	$-46 \leq h \leq 46$
	$-9 \leq k \leq 11$
	$-28 \leq l \leq 28$
Reflections collected	118739
Independent reflections	7387 [R_{int} = 0.0562]
Completeness to θ = 52.48°	100.0 %
Absorption correction	Multi-scan
Maximum and minimum transmission	0.7462 and 0.6631
Refinement method	Full-matrix least-squares on F^2
Data / restraints / parameters	7387 / 0 / 324
Goodness-of-fit on F^{2a}	0.971
Final R indices [I > 2σ(I) = 4426 data]^b	R_1 = 3.60 %, wR_2 = 11.48 %
R indices (all data, 0.80 Å)	R_1 = 5.90 %, wR_2 = 13.58 %
Largest diff. peak and hole	0.85 and –0.88 e/Å^{-3}

^{a} GooF = $[\Sigma[w(F_o^2-F_c^2)^2] / (n-p)]^{1/2}$ where n is the number of reflections and p is the total number of parameters refined. $^b R_1 = \Sigma||F_o|-|F_c|| / \Sigma|F_o|$; $wR_2 = [\Sigma[w(F_o^2-F_c^2)^2] / \Sigma[w(F_o^2)^2]]^{1/2}$

Table S4 | Crystallographic information for the structural refinement of **4**.

Empirical Formula	$C_{14}H_{26}N_4Zn$
Formula weight	315.78 g/mol
Temperature	100.00(1)
Wavelength	0.71073 Å
Crystal System	orthorhombic
Space Group	*Pbcn*
Unit Cell Dimensions	a = 12.0640(4) Å, α = 90˚
	b = 11.9611(5) Å, β = 90˚
	c = 10.8780(3) Å, γ = 90˚
Volume	1570.34(9) $Å^3$
Z	4
Density (calculated)	1.326 Mg/m^3
Absorption coefficient	1.558 mm^{-1}
F_{000}	672.0
Crystal color	White (Transparent)
Crystal size	0.364 × 0.095 × 0.072 mm^3
θ range	4.794 to 71.472˚
Index ranges	$-13 \le h \le 18$
	$-16 \le k \le 18$
	$-17 \le l \le 15$
Reflections collected	13850
Independent reflections	3287 [R_{int} = 0.0317]
Completeness to θ = 52.48˚	100 %
Absorption correction	Multi-scan
Maximum and minimum transmission	1.000 and 0.561
Refinement method	Full-matrix least-squares on F^2
Data / restraints / parameters	3287 / 0 / 91
Goodness-of-fit on F^{2a}	1.025
Final R indices [I > 2σ(I) = 19146 data][b]	R_1 = 2.83 %, wR_2 = 6.87 %
R indices (all data, 0.80 Å)	R_1 = 4.47 %, wR_2 = 7.57 %
Largest diff. peak and hole	0.49 and –0.45 e/$Å^{-3}$

[a] GooF = $[\Sigma[w(F_o^2-F_c^2)^2] / (n-p)]^{1/2}$ where n is the number of reflections and p is the total number of parameters refined. [b] $R_1 = \Sigma||F_o|-|F_c|| / \Sigma|F_o|$; $wR_2 = [\Sigma[w(F_o^2-F_c^2)^2] / \Sigma[w(F_o^2)^2]]^{1/2}$

Table S4 | Crystallographic information for the structural refinement of **5**.

Empirical Formula	$C_{12}H_{18}NiN_2O_2 \cdot 0.5(H_2O)$
Formula weight	291.01 g/mol
Temperature	100(2) K
Wavelength	0.71073 Å
Crystal System	Monoclinic
Space Group	*C*2/*c*
Unit Cell Dimensions	a = 22.1196(18) Å, α = 90.0°
	b =8.3402(6) Å, β = 128.021(5)°
	c = 17.740(2) Å, γ = 90.0°
Volume	2578.1(3) $Å^3$
Z	8
Density (calculated)	1.499 Mg/m^3
Absorption coefficient	1.502 mm^{-1}
F_{000}	1232.0
Crystal color	Orange Red
Crystal size	0.564 × 0.115 × 0.078 mm^3
θ range	4.676 to 57.206°
Index ranges	$-29 \leq h \leq 29$
	$-11 \leq k \leq 11$
	$-23 \leq l \leq 23$
Reflections collected	20696
Independent reflections	3272 [R_{int} = 0.1044]
Completeness to θ = 52.48°	100 %
Absorption correction	Multi-scan
Maximum and minimum transmission	0.7457 and 0.5825
Refinement method	Full-matrix least-squares on F^2
Data / restraints / parameters	3272 / 0 / 166
Goodness-of-fit on F^{2a}	1.022
Final R indices [I > 2σ(I) = 10408 data][b]	R_1 = 4.81 %, wR_2 = 10.44 %
R indices (all data, 0.80 Å)	R_1 = 7.98 %, wR_2 = 11.70 %
Largest diff. peak and hole	0.64 and –0.72 e/$Å^{-3}$

[a] GooF = $[\Sigma[w(F_o^2 - F_c^2)^2] / (n-p)]^{1/2}$ where n is the number of reflections and p is the total number of parameters refined. [b] $R_1 = \Sigma||F_o| - |F_c|| / \Sigma|F_o|$; $wR_2 = [\Sigma[w(F_o^2 - F_c^2)^2] / \Sigma[w(F_o^2)^2]]^{1/2}$

Table S5 | Highlighted structural parameters for **1−3**. In the case of **1** and **3**, E_1 and E_2 are nitrogen atoms. In the case of **2**, E_1 and E_2 are oxygen atoms. **2** and **3** have two unique positions in the unit cell with slightly different geometries, both of which are described.

Parameter	**1**	**2**	**3**	**4**	**5**	**6**
Dihedral Angle	60.8°	4.2°	6.1°	75.33°	1.86°	2.7°
M-N_1	1.954	1.931	1.935	1.989	1.849	1.874
M-N_2	1.955	1.928	1.926	1.995	1.845	1.874
M-E_1	1.955	1.920	1.922	1.989	1.856	1.875
M-E_2	1.955	1.916	1.944	1.995	1.850	1.881
M-N/E_{avg}	1.955	1.924	1.932	1.992	1.850	1.876
N_1-M-E_1	94.83°	93.98	95.07	95.98	94.75	94.67
N_1-M-E_2	100.59°	175.34°	176.53°	125.99°	177.79°	176.83°
N_1-M-N_2	136.33°	85.98°	84.19°	95.98°	87.23°	84.81°
N_2-M-E_1	101.58°	86.28°	96.14°	108.63°	94.71°	95.20°
N_2-M-E_2	94.83°	174.87°	175.19°	125.99°	177.44°	179.32°
E_1-M-E_2	136.17°	86.08°	84.32°	108.63°	83.30°	85.30°
τ_4'	0.62	0.066	0.052	0.76	0.030	0.017
Second Site in Unit Cell						
M-N_1	---	1.936	1.925	---	---	1.853
M-N_2	---	1.934	1.929	---	---	1.871
M-E_1	---	1.919	1.927	---	---	1.853
M-E_2	---	1.920	1.925	---	---	1.877
Cu-N/E_{avg}	---	1.925	1.927	---	---	1.863
N_1-M-E_1	---	93.70	95.79°	---	---	93.98°
N_1-M-E_2	---	177.41°	177.15°	---	---	179.09°
N_1-M-N_2	---	85.54°	84.019°	---	---	85.60°
N_2-M-E_1	---	177.47°	84.46°	---	---	177.40°
N_2-M-E_2	---	93.60°	95.45°	---	---	85.27°
E_1-M-E_2	---	93.60°	95.45°	---	---	95.10°
τ_4'	---	0.030	0.049	---	---	0.017
Avg Cu-N between both sites in **3**	---	1.9245	1.9295	---	---	1.870

Table S6 | Highlighted structural parameters for **1−3** relaxed with the B3LYP functional. In the case of **1** and **3**, E_1 and E_2 are nitrogen atoms. In the case of **2**, E_1 and E_2 are oxygen atoms.

Parameter	**1**	**2**	**3**
Dihedral Angle	50.3°	10.0°	6.1°
Cu-N_1	1.990	1.959	1.945
Cu-N_2	1.990	1.959	1.944
Cu-E_1	1.990	1.943	1.944
Cu-E_2	1.990	1.943	1.945
Cu-N/E_{avg}	1.990	1.951	1.945
N_1-Cu-E_1	94.41	93.80	95.69
N_1-Cu-E_2	98.88	172.59	176.84
N_1-Cu-N_2	140.16	85.97	84.21
N_2-Cu-E_1	99.07	172.62	84.22
N_2-Cu-E_2	94.41	93.80	95.70
E_1-Cu-E_2	140.02	87.37	176.90
τ_4'	0.5631	0.1024	0.0418

Table S7 | Highlighted structural parameters for **1−3** relaxed with the PBE functional. In the case of **1** and **3**, E_1 and E_2 are nitrogen atoms. In the case of **2**, E_1 and E_2 are oxygen atoms.

Parameter	**1**	**2**	**3**
Dihedral Angle	56.4°	10.0°	6.1°
Cu-N_1	1.971	1.949	1.938
Cu-N_2	1.971	1.949	1.938
Cu-E_1	1.971	1.943	1.938
Cu-E_2	1.971	1.943	1.938
Cu-N/E_{avg}	1.990	1.946	1.938
N_1-Cu-E_1	95.96	93.80	95.59
N_1-Cu-E_2	101.67	172.59	179.43
N_1-Cu-N_2	133.87	85.97	84.41
N_2-Cu-E_1	101.77	172.62	84.41
N_2-Cu-E_2	95.96	93.80	95.59
E_1-Cu-E_2	133.87	87.37	179.45
τ_4'	0.6517	0.1024	0.0055

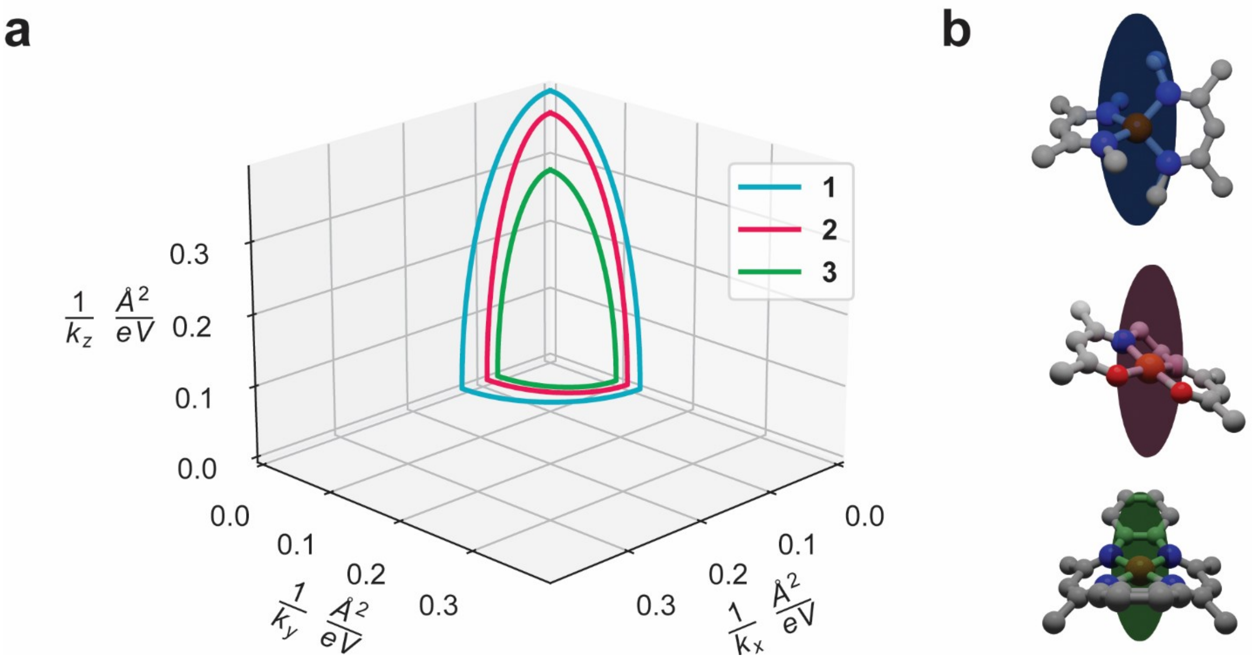

Figure S1 | Compliance of the copper metal center for **1–3**. A larger value indicates that there is less resistance to motion of the copper along that axis. Though this metric does not fully capture the rigidity of ligand π-bonds, it does demonstrate how tightly bound the spin bearing metal center is in the binding pocket. (**a**) Compliance ellipsoids for **1–3** viewed on the same axis. The compliance increases in the order of **1** < **2** < **3** in all directions. The compliance of **3** is markedly reduced (rigidity increased) compared to **1** and **2** in all directions. **2** and **3** have similar compliance in the xy plane, likely as a result of the square planar geometry. (**b**) Compliance ellipsoids visualized over the molecular structures of **1**–**3**.

Table S8 | The energies of the spin-up and spin down molecular orbitals in **1** derived from the atomic d orbitals, as well as the percent-d orbital character of the molecular orbital. The HOMO orbital of the system (the highest energy orbital with an occupancy of 1) is arbitrarily set to 0 eV.

d Orbital	Spin-up Orbital Energy (eV)	d Orbital Character (%)	Spin-down Orbital Energy (eV)	d Orbital Character (%)
d_{xz}	–3.280	35.6	–2.843	47.4
d_{yz}	–3.178	55.0	–2.736	74.2
d_{xy}	–3.042	75.5	–2.485	36.3
d_{z^2}	–2.335	47.1	–1.146	42.4
$d_{x^2-y^2}$	0	20.1	3.952	58.1

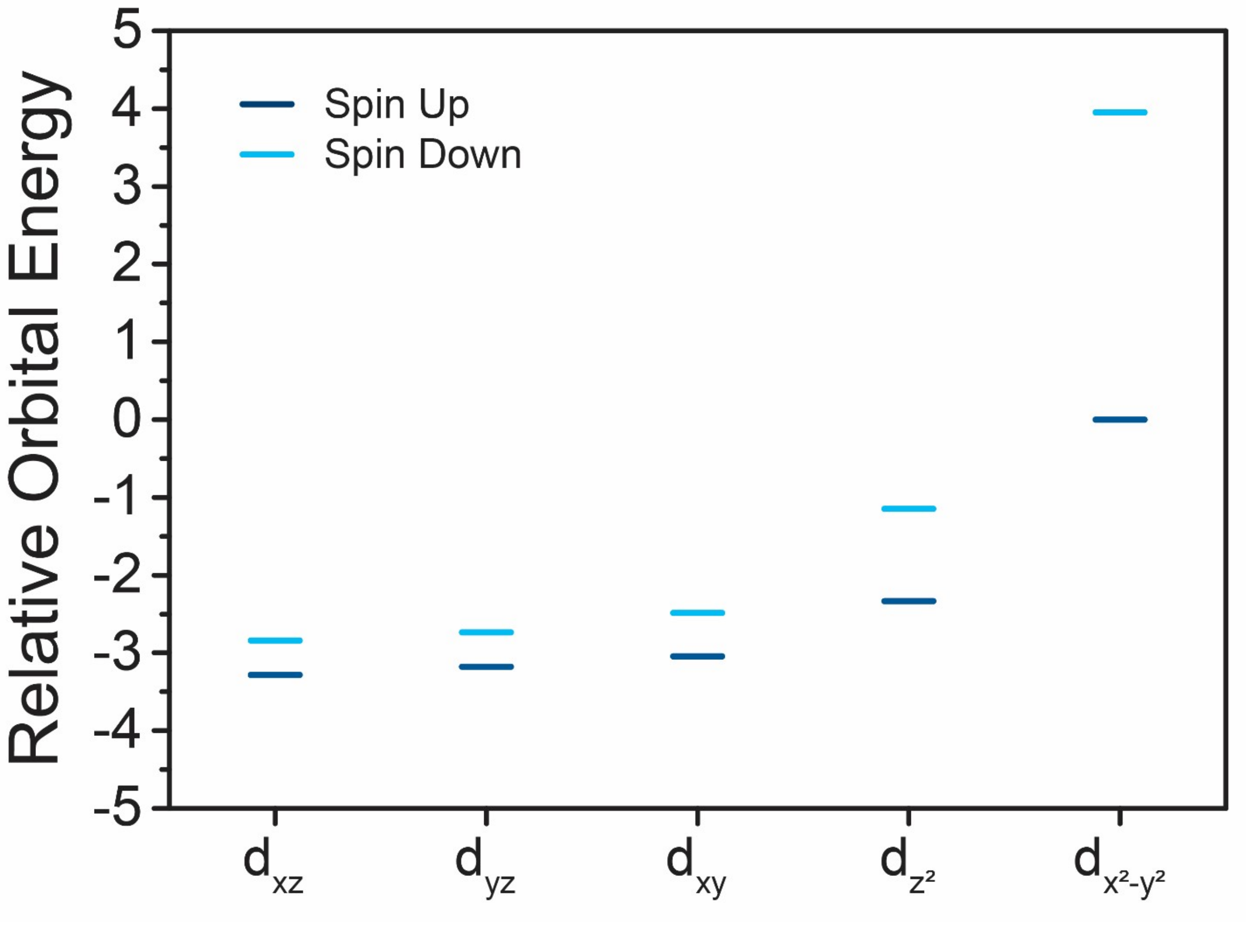

Figure S2 | Energy of the spin-up and spin-down molecular orbitals derived from the copper d-orbitals in **1** (in eV), and the percent character of the molecular orbital comprised of the atomic d-orbital. Numbers index molecular orbitals in ascending energy. The HOMO orbital is assigned to 0 eV. The displayed orbitals are the five orbitals with the highest %d-orbital character.

Table S9 | The energies of the spin-up and spin down molecular orbitals in **2** derived from the atomic d orbitals, as well as the percent-d orbital character of the molecular orbital. The HOMO orbital of the system (the highest energy orbital with an occupancy of 1) is arbitrarily set to 0 eV.

d Orbital	Spin-up Orbital Energy (eV)	d Orbital Character (%)	Spin-down Orbital Energy (eV)	d Orbital Character (%)
d_{xy}	–3.533	56.1	–3.240	62.4
d_{z^2}	–3.145	35.6	–2.797	31.8
d_{yz}	–2.866	39.7	–2.600	23.7
d_{sz}	–2.399	20.7	–2.181	35.9
$d_{x^2-y^2}$	0	20.9	3.877	53.4

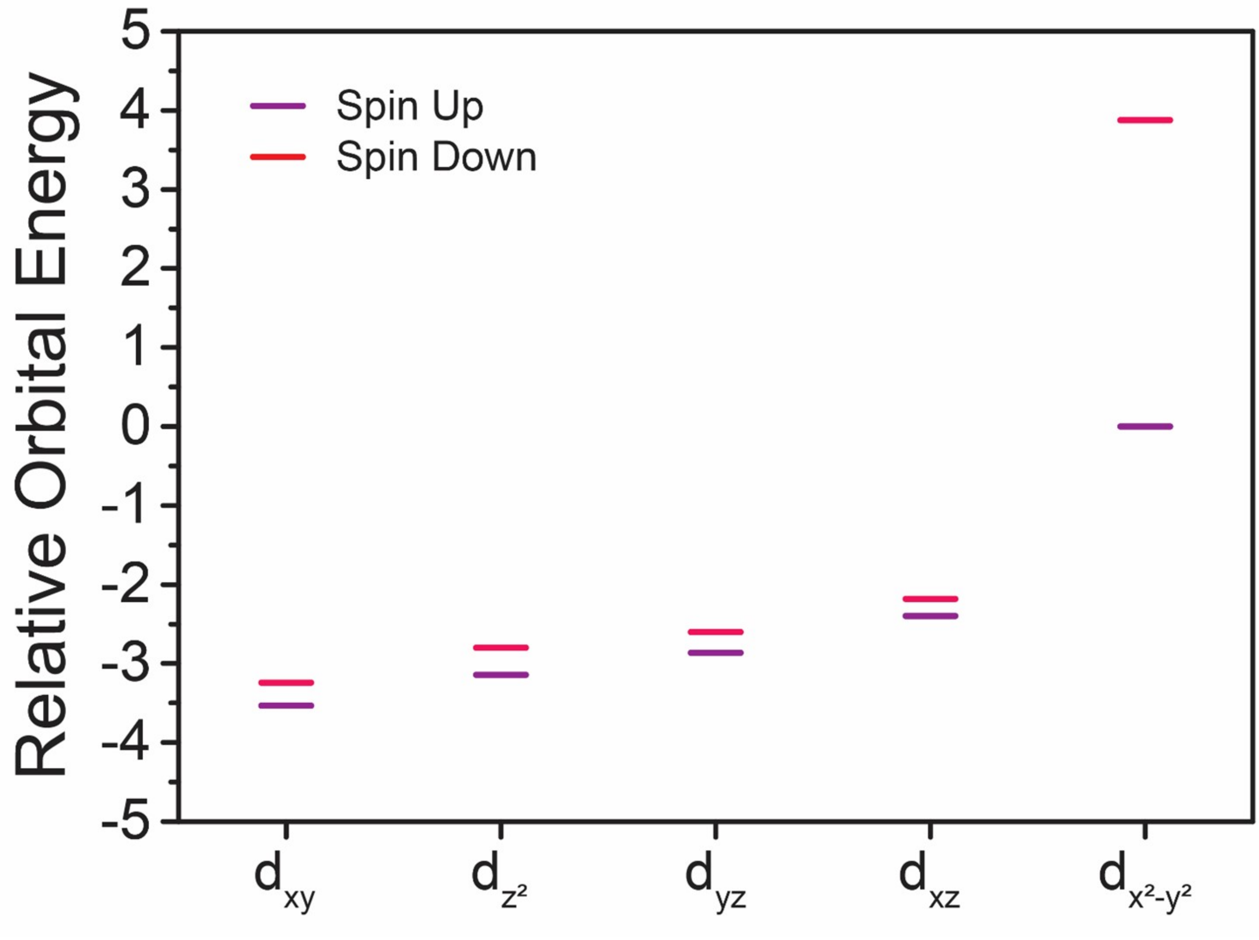

Figure S3 | Energy of the spin-up and spin-down molecular orbitals derived from the copper d-orbitals in **2** (in eV) and the percent character of the molecular orbital comprised of the atomic d-orbital. Numbers index molecular orbitals in ascending energy. The HOMO orbital is assigned to 0 eV. The displayed orbitals are the five orbitals with the highest %d-orbital character.

Table S10 | The energies of the spin-up and spin down molecular orbitals in **3** derived from the atomic d orbitals, as well as the percent-d orbital character of the molecular orbital. The HOMO orbital of the system (the highest energy orbital with an occupancy of 1) is arbitrarily set to 0 eV.

d Orbital	Spin-up Orbital Energy (eV)	d Orbital Character (%)	Spin-down Orbital Energy (eV)	d Orbital Character (%)
d_{xy}	–4.089	74.3	–3.894	54.8
d_{z^2}	–3.344	63.1	–2.956	63.5
d_{yz}	–3.164	66.1	–2.685	69.4
d_{sz}	–2.816	29.8	–2.566	28.3
$d_{x^2-y^2}$	0	27.3	4.416	30.8

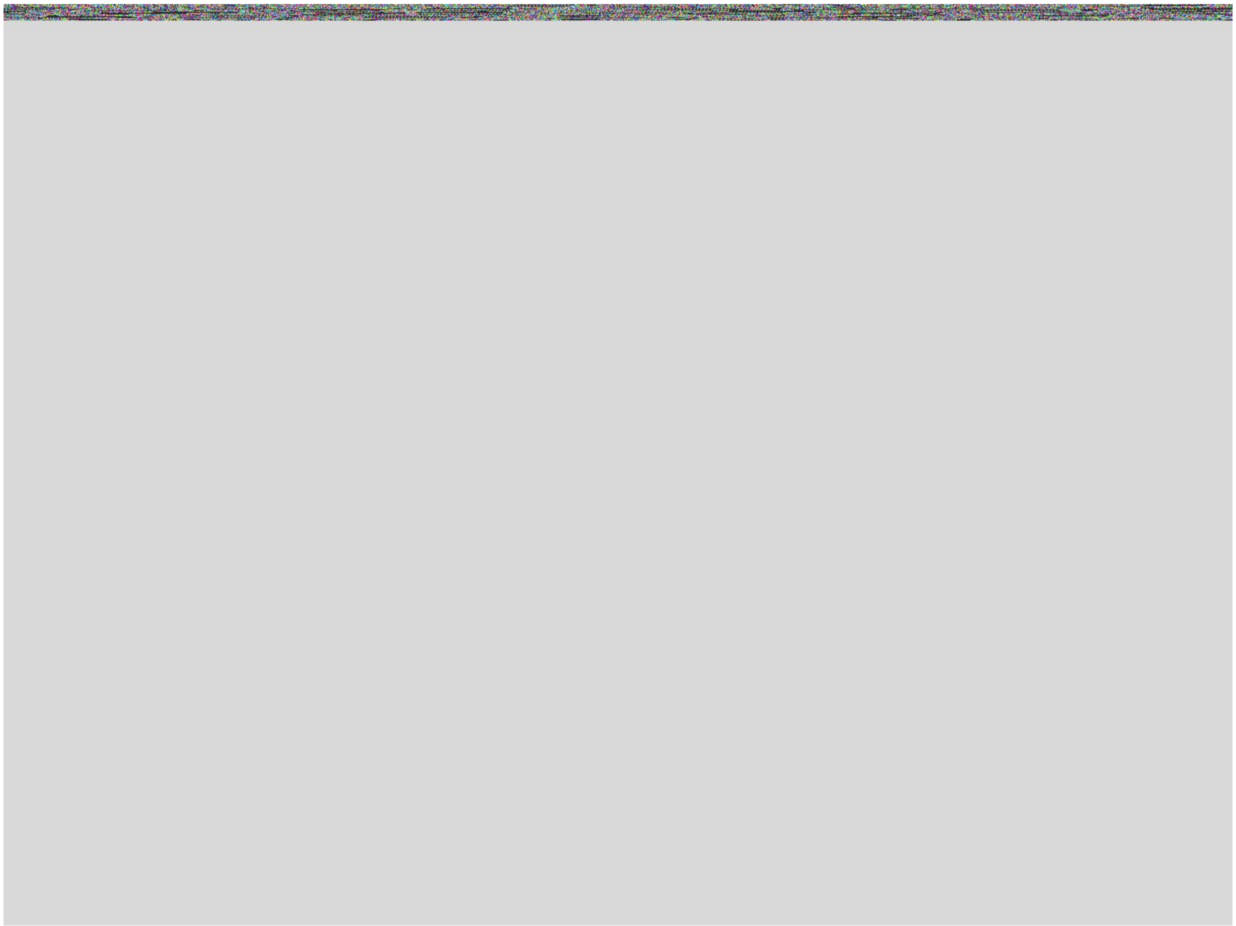

Figure S4 | Energy of the spin-up and spin-down molecular orbitals derived from the copper d-orbitals in **3** (in eV) and the percent character of the molecular orbital comprised of the atomic d-orbital. Numbers index molecular orbitals in ascending energy. The HOMO orbital is assigned to 0 eV. The displayed orbitals are the five orbitals with the highest %d-orbital character.

Table S11 | The combined cw-EPR fit parameters for **1'−3'** at 5 K, and **1−3** in OTP (0.1 weight percent, 60 K, **1'''−3'''**). The errors given are approximate errors from simulating the spectra.

	**1'**	**1'''**	**2'**	**2'''**	**3'**	**3'''**
$g_{\perp}$	2.055(5)	2.05(5)	2.036(4)	2.045(5)	2.06(2)	2.025(5)
$g_{\parallel}$	2.205(5)	2.200(5)	2.169(1)	2.18(1)	2.17(1)	2.18(1)
$A_{Cu,\perp}$	40(5)	40(10)	63(8)	100(10)	50(20)	65(5)
$A_{Cu,\parallel}$	440(20)	460(10)	650(20)	650(20)	630(20)	620(15)
$A_{N,\perp}$	10(5)	15(5)	50(3)	40(5)	50(15)	58(3)
$A_{N,\parallel}$	20(5)	20(5)	45(3)	45(5)	45(10)	45(3)
Lorentzian Broadening	----	----	0.96	1.1	----	1.68
H-Strain	[140 140 190]	[110 110 140]	----	----	----	----
g-Strain	----	----	----	----	[0.012 0.012 0.016]	----

Table S12 | Computed *A* and *g* tensors for all three molecules. *A* tensor is in MHz.

	**1**	**2**	**3**
g_x	2.0504	2.0390	2.0310
g_y	2.0509	2.0411	2.0349
g_z	2.1508	2.1355	2.1390
$A_{N,x}$	28.7	40.0	34.1
$A_{N,y}$	29.8	41.5	34.8
$A_{N,z}$	41.2	54.6	47.4
$A_{Cu,x}$	35.7	−61.4	−51.5
$A_{Cu,y}$	11.9	−64.9	−53.2
$A_{Cu,z}$	−518.48	−655.37	−622

Table S13 | The spin density of the unpaired electron spin on the copper and the directly bound donor atom (O or N). As the antibonding character of the orbital the electron is in increases, the spin becomes increasing localized on the ligand, represented by a decrease in ρ_{Cu} and an increase in $\rho_{E,Avg}$. The decreased spin density on the ligand in **2** is a result of weaker resonance in the ligand. In viewing the ligands of **1−3**, the acacen ligand in **2** has weaker resonance contributions from structures which place increased spin density on carbon, causing a comparatively greater $\rho_{E,Avg}$ than would otherwise be expected.

	**1**	**2**	**3**
ρ_{Cu}	0.599	0.591	0.541
$\rho_{E,Avg}$	0.088	0.096	0.092

Table S14 | Saturation recovery fit parameters for **1′**

Temperature (K)	T_1 (μs)	σ	A	I_0
5	5210(360)	0.340(10)	1.16(3)	0.10(2)
10	1420(14)	0.713(6)	0.987(5)	0.0126(5)
20	147.00(7)	0.912(4)	0.980(3)	0.0112(3)
30	41.77(2)	0.957(5)	0.981(3)	0.0018(7)
40	16.90(9)	0.959(5)	0.966(4)	0.0124(3)
50	7.21(6)	0.940(8)	0.972(6)	0.0112(4)
60	2.98(2)	0.97(1)	0.984(7)	−0.010(2)
70	1.35(5)	1.00(3)	0.98(3)	0.018(2)
80	0.83(12)	1.0(1)	0.87(12)	0.945(4)
90	0.47(50)	1.0(7)	0.845(8)	0.155(7)

Table S15 | Saturation recovery fit parameters for **1″**

Temperature (K)	T_1 (μs)	σ	A	I_0
5	3870(100)	0.470(7)	0.989(5)	0.008(3)
10	373(14)	0.66(2)	0.936(8)	0.024(5)
20	48(2)	0.81(4)	0.95(1)	0.041(5)
30	17.3(8)	0.79(5)	0.96(1)	0.025(6)
40	10.0(8)	0.77(8)	0.94(3)	0.06(1)
50	5.25(8)	0.911(7)	1.090(8)	0.0101(4)
60	2.44(3)	0.96(1)	0.935(9)	0.0122(4)
70	1.54(4)	0.99(2)	1.25(2)	0.0178(8)
80	0.80(3)	0.83(3)	1.90(3)	−0.08(1)
90	0.68(3)	0.94(9)	2.2(2)	−0.21(4)

Table S16 | Saturation recovery fit parameters for **2′**

Temperature (K)	T_1 (μs)	σ	A	I_0
5	14000(300)	0.548(6)	1.006(5)	0.002(3)
10	1830(10)	0.783(6)	0.994(2)	0.000(2)
20	172(1)	0.864(6)	0.996(2)	0.002(2)
30	53.2(4)	0.910(8)	0.997(2)	0.006(1)
40	22.3(2)	0.906(1)	0.997(2)	0.009(1)
50	12.0(1)	0.936(1)	0.999(3)	0.12(1)
60	7.20(7)	0.924(1)	1.016(4)	0.009(2)
70	4.54(5)	0.97(1)	1.014(5)	0.010(1)
80	3.43(1)	0.979(6)	1.025(2)	0.0021(8)
90	2.43(1)	0.985(8)	1.033(3)	0.0046(9)
100	1.92(1)	0.98(1)	1.050(5)	0.005(1)
120	1.09(1)	0.97(2)	1.090(9)	0.009(2)
140	0.76(1)	1.00(1)	1.12(1)	0.009(1)
160	0.57(2)	1.00(4)	1.17(2)	0.013(2)
180	0.39(3)	0.97(8)	1.23(6)	0.049(4)
200	0.27(7)	0.9(2)	1.38(3)	0.06(1)
220	0.25(1)	1.2(3)	1.3(3)	0.09(1)
240	0.23(8)	1.4(7)	1.1(4)	0.14(2)
260	0.27(3)	2.0(8)	1.0(2)	0.13(2)

Table S17 | Saturation recovery fit parameters for **2″**

Temperature (K)	T_1 (μs)	σ	A	I_0
5	7060(80)	0.825(9)	0.993(3)	0.005(2)
10	960(10)	0.86(1)	0.992(4)	0.00(2)
20	126(1)	0.930(9)	0.993(3)	−0.001(1)
30	42.9(3)	0.932(8)	0.997(2)	−0.024(1)
40	18.5(1)	0.94(1)	0.994(6)	−0.006(5)
50	10.6(2)	0.93(1)	1.004(7)	0.017(3)
60	6.7(2)	0.93(4)	1.00(1)	0.02(1)
70	4.2(1)	0.90(4)	1.02(2)	0.021(6)
80	3.2(1)	1.05(6)	0.99(2)	0.025(7)
90	2.10(8)	0.98(6)	1.01(2)	0.044(6)
100	1.43(1)	0.97(3)	1.071(8)	0.010(2)
120	1.00(4)	0.99(5)	1.08(3)	0.029(3)
140	0.90(2)	1.03(3)	1.1(1)	0.010(2)
160	0.570(5)	0.99(1)	1.191(8)	0.0053(7)
180	0.40(2)	1.02(2)	1.25(5)	0.022(3)
200	0.38(1)	1.4(1)	1.13(4)	0.033(4)
220	0.28(2)	1.1(1)	1.3(1)	0.028(5)
240	0.23(6)	1.6(8)	1.2(4)	0.13(2)
260	0.2(1)	0.7(4)	2(1)	0.11(2)

Table S18 | Saturation recovery fit parameters for **3′**

Temperature (K)	T_1 (μs)	σ	A	I_0
5	10700(260)	0.635(11)	0.994(5)	0.007(3)
10	4060(30)	0.727(5)	1.000(1)	0.003(1)
20	764(4)	0.870(5)	0.993(1)	0.0051(8)
30	280(1)	0.928(6)	0.991(1)	0.0062(8)
40	136.3(5)	0.946(4)	0.994(1)	0.0045(4)
50	72.4(4)	0.946(6)	0.996(2)	0.0080(9)
60	37.1(2)	0.958(7)	0.990(2)	0.0114(9)
70	23.8(3)	0.922(14)	0.997(4)	0.078(1)
80	13.9(2)	0.91(2)	0.981(2)	0.020(2)
90	8.71(2)	0.96(3)	0.99(1)	0.026(2)
100	5.2(4)	0.83(8)	0.99(4)	0.056(1)
120	4.2(7)	0.86(6)	0.98(3)	0.045(7)
140	2.0(1)	0.88(1)	1.05(6)	0.042(9)
160	1.50(7)	1.00(3)	1.02(3)	0.042(5)
180	0.93(4)	1.01(6)	1.08(3)	0.035(3)
200	0.66(5)	1.2(1)	1.05(4)	0.035(4)
220	0.47(6)	1.1(2)	1.11(1)	0.089(9)
240	0.37(4)	1.0(1)	1.2(1)	0.051(6)
260	0.30(6)	1.1(3)	1.2(2)	0.09(1)
280	0.25(4)	1.3(3)	1.3(3)	0.07(1)
300	0.22(5)	1.4(5)	1.3(4)	0.09(2)

Table S19 | Saturation recovery fit parameters for **3″**

Temperature (K)	T_1 (μs)	σ	A	I_0
5	1320(10)	0.602(3)	1.005(8)	0.004(1)
10	537(6)	0.656(6)	0.990(2)	0.012(1)
20	162(1)	0.734(5)	0.993(1)	0.0082(4)
30	76.6(8)	0.747(7)	0.996(3)	0.005(1)
40	39.2(5)	0.75(1)	0.995(4)	0.010(1)
50	25.1(3)	0.80(1)	1.002(4)	0.006(1)
60	16.7(2)	0.85(1)	1.000(5)	0.012(2)
70	11.0(3)	0.88(3)	0.996(9)	0.009(4)
80	6.5(1)	0.84(2)	1.017(9)	0.016(3)
90	4.2(1)	0.86(4)	1.02(2)	0.029(5)
100	2.90(6)	0.84(4)	1.05(1)	0.019(2)
120	1.85(5)	0.90(4)	1.05(2)	0.020(3)
140	1.17(5)	0.96(5)	1.07(3)	0.027(4)
160	0.76(4)	0.92(6)	1.13(4)	0.019(5)
180	0.54(8)	0.92(7)	1.19(6)	0.037(0)
200	0.43(3)	1.0(1)	1.22(8)	0.025(5)
220	0.36(3)	1.3(2)	1.2(1)	0.058(8)
240	0.3(1)	1.0(3)	1.2(3)	0.10(1)
260	0.2(2)	0.7(4)	1.9(1)	0.11(2)
280	0.30(7)	1.8(1)	0.9(3)	0.22(2)

Table S20 | Fit parameters for 2 pulse Hahn echo decay measurements for **1′**

Temperature (K)	T_m (μs)	A	I_0
5	0.32(2)	1.0(1)	0.008(3)
10	0.37(6)	1.0(2)	0.067(6)
20	0.64(4)	1.0(1)	0.005(4)
30	0.391(8)	0.91(9)	0.11(3)
40	0.599(6)	1.0(2)	0.001(4)
50	0.47(7)	1.0(3)	0.000(2)
60	0.56(2)	1.00(4)	−0.015(4)
70	0.47(2)	1,00(5)	−0.010(3)
80	0.32(3)	1.0(2)	−0.004(2)
90	0.32(2)	1.0(1)	0.008(3)

Table S21 | Fit parameters for 2 pulse Hahn echo decay measurements for **1″**

Temperature (K)	T_m (μs)	A	I_0
5	14.1(2)	0.81(1)	0.11(1)
10	14.3(6)	0.78(1)	0.11(1)
20	11.1(2)	0.789(9)	0.068(1)
30	5.3(2)	0.77(1)	−0.004(4)
40	2.7(1)	0.64(1)	−0.011(3)
50	0.49(2)	0.63(3)	−0.012(4)
60	0.32(3)	0.54(3)	−0.003(3)
70	0.36(4)	0.61(3)	−0.005(5)
80	0.33(1)	0.58(1)	−0.004(2)
90	0.33(1)	0.59(1)	−0.012(5)

Table S22 | Fit parameters for 2 pulse Hahn echo decay measurements for **2′**

Temperature (K)	T_m (μs)	A	I_0
5	2.82(6)	1.00(9)	0.014(5)
10	2.88(6)	1.00(9)	0.011(4)
20	2.55(5)	1.00(1)	0.014(4)
30	1.99(9)	1.00(2)	0.04(1)
40	1.37(3)	1.00(1)	0.024(5)
50	1.12(2)	1.00(1)	0.017(3)
60	0.452(6)	1.00(9)	−0.004(1)
70	0.37(3)	1.00(7)	0.002(3)
80	0.452(6)	1.00(1)	0.001(2)
90	0.485(7)	1.00(1)	0.002(2)
100	0.466(7)	1.00(1)	0.002(2)
120	0.409(6)	1.00(1)	0.001(2)
140	0.43(3)	1.00(6)	0.003(3)
160	0.292(6)	1.00(1)	−0.000(1)
180	0.257(9)	1.00(2)	0.001(4)
200	0.21(1)	1.00(3)	0.006(5)
220	0.16(1)	0.96(5)	0.002(6)
240	0.19(2)	0.99(6)	0.006(9)

Table S23 | Fit parameters for 2 pulse Hahn echo decay measurements for **2″**

Temperature (K)	T_m (μs)	A	I_0
5	6.20(4)	1.000(3)	−0.057(1)
10	5.88(4)	1.000(4)	−0.095(1)
20	7.2(1)	1.005(6)	−0.058(4)
30	3.06(3)	0.897(5)	0.023(2)
40	2.21(2)	0.903(5)	0.023(1)
50	1.68(2)	0.895(7)	0.032(1)
60	1.30(2)	0.900(7)	0.022(3)
70	1.14(2)	0.936(7)	0.012(3)
80	1.07(2)	0.912(8)	0.038(3)
90	1.0(2)	1.00(1)	−0.116(3)
100	1.0679 (3)	1.000(1)	−0.00254(6)
120	0.66(1)	0.954(9)	0.054(1)
140	0.546(8)	1.000(9)	0.044(2)
160	0.491(6)	1.000(8)	−0.044(1)
180	0.43(1)	1.00(1)	0.000(3)
200	0.41(1)	1.00(1)	0.002(2)
220	0.33(1)	0.999(1)	0.00206(1)
240	0.316(8)	1.00(1)	0.006(3)
260	0.275(8)	1.00(2)	−0.001(3)

Table S24 | Fit parameters for 2 pulse Hahn echo decay measurements for **3′**

Temperature (K)	T_m (μs)	A	I_0
5	1.49(8)	0.887(7)	−0.0019(9)
10	1.84(2)	0.874(7)	−0.001(1)
20	2.03(2)	0.873(7)	0.000(1)
30	2.05(2)	0.871(7)	0.000(1)
40	2.01(2)	0.869(6)	0.000(1)
50	1.98(2)	0.873(7)	0.002(1)
60	1.77(2)	0.897(7)	0.000 (1)
70	0.80(1)	1.00(3)	−0.001(3)
80	0.79(1)	0.945(9)	0.0000(1)
90	0.574(9)	1.000(9)	−0.0002(8)
100	0.38(3)	1.00(7)	−0.002(3)
120	0.355(8)	1.00(1)	0.004(3)
140	0.50(1)	1.00(1)	0.001(3)
160	0.79(2)	1.00(1)	−0.009(5)
180	0.79(2)	1.00 (1)	−0.012(5)
200	0.67(2)	1.00(1)	0.009(4)
220	0.60(1)	1.00(1)	−0.006(4)
240	0.53(1)	1.00(1)	−0.005(4)
260	0.47(1)	1.00(2)	−0.003(3)
280	0.43(1)	1.00(2)	0.001(3)
300	0.43(1)	1.00(2)	−0.005(4)

Table S25 | Fit parameters for 2 pulse Hahn echo decay measurements for **3″**

Temperature (K)	T_m (μs)	A	I_0
5	3.98(4)	0.837(5)	0.0046(1)
10	3.58(3)	0.859(5)	0.004(1)
20	3.26(3)	0.872(5)	0.001(1)
30	1.97(2)	0.882(6)	0.005(1)
40	1.40(2)	0.913(7)	0.0054(9)
50	1.75(2)	0.929(5)	0.006(1)
60	2.12(2)	0.907(5)	0.006(2)
70	2.12(2)	0.912(6)	0.007(1)
80	1.82(2)	0.920(6)	0.004(1)
90	1.82(2)	0.920(6)	0.004(1)
100	0.70(1)	1.000(9)	0.003(1)
120	0.552(9)	1.000(9)	0.019(2)
140	0.543(9)	1.000(1)	0.019(3)
160	0.75(2)	1.00(1)	0.006(4)
180	0.75(2)	1.00(1)	0.004(4)
200	0.67(2)	1.00(1)	0.001(4)
220	0.59(1)	1.00(1)	0.005(4)
240	0.50(1)	1.00(1)	0.002(4)
260	0.47(1)	1.00(1)	0.001(4)
280	0.44(3)	1.00(1)	−0.005(4)

Table 26 | Fit parameters extracted from fits to **1'−3'** and **1"−3"**. When fitting **1"−3"**, the Debye temperature was held to a constant, arbitrary value to reflect that all measurements were performed in the same matrix (OTP). This is signified by boldface text.

	1'	1"	2'	2"	3'	3"
A_{Dir} (s^{-1} K^{-1})	39(4)	65(25)	13.8(4)	32(6)	18(1)	160(20)
B_{Ram} (s^{-1}) x 10^5	15(5)	7.3(3)	2(1)	10.8(5)	2.7(2)	3.7(1)
Θ_D (K)	75(10)	**65**	63(8)	**65**	81(4)	**65**
C_{Loc} (s^{-1}) x 10^7	18.5(9)	56(4)	0.6(1)	0.32(5)	1.1(1)	0.8(1)
Δ_{Loc} (s^{-1})	290(40)	250(30)	213(25)	192(15)	328(15)	273(18)

Table 27 | Room temperature Rabi rate for **3'** at variable microwave power

	1 dB	3 dB	7.2 dB
Rabi Rate (MHz)	27.3	19.5	11.7

Table S28 | Linear fit for *g*-tensor for **1** with eq. 1 ($g_{ii} = (b(g_{ii}) * x) + g_{eq}$) for each component of the *g*-tensor (x, y, and z). The r^2 value is the coefficient of determination for each linear regression.

Mode	Frequency	$b(g_{xx})$	$r^2\ (g_{xx})$	$b(g_{yy})$	$r^2\ (g_{yy})$	$b(g_{zz})$	$r^2\ (g_{zz})$
3	7.40	0.0000	0.784	0.0000	0.988	0.0001	0.971
4	14.77	0.0000	0.814	0.0000	0.352	−0.0000	0.013
5	20.09	0.0000	0.485	−0.0000	0.906	−0.0001	0.681
6	38.38	−0.0005	0.688	0.0005	0.670	0.0001	0.507
7	40.67	0.0007	0.999	0.0000	0.935	0.0002	0.934
8	56.96	−0.0014	0.999	−0.0006	0.999	−0.0030	0.999
9	74.64	−0.0001	0.000	0.0001	0.000	−0.0000	0.350
10	90.59	−0.0002	0.939	0.0002	0.952	−0.0001	0.835
11	112.12	0.0026	0.999	0.0003	0.998	−0.0045	0.999
12	115.41	−0.0011	0.999	−0.0000	0.995	0.0025	0.999
13	132.06	−0.0001	0.996	−0.0002	0.998	−0.0006	0.999
14	133.15	−0.0002	0.999	0.0003	0.999	0.0020	1.000
15	143.79	0.0002	0.997	0.0000	0.988	0.0004	0.997
16	145.55	0.0004	0.984	−0.0000	0.717	0.0004	0.994
17	149.51	0.0102	0.999	0.0056	0.999	0.0189	1.000
18	158.63	−0.0001	0.985	0.0002	0.609	0.0000	0.000
19	161.12	−0.0026	0.040	0.0026	0.040	−0.0000	0.709
20	188.66	0.0019	0.999	0.0008	0.999	−0.0019	0.999
21	189.18	0.0000	0.150	−0.0001	0.990	0.0001	0.871
22	211.25	−0.0066	0.055	0.0066	0.053	−0.0002	0.032
23	219.52	−0.0002	0.999	−0.0002	0.999	−0.0003	0.998
24	225.45	−0.0011	0.354	0.0009	0.275	−0.0002	0.987
25	227.33	0.0001	0.509	−0.0000	0.478	0.0001	0.147
26	230.58	−0.0077	0.999	−0.0081	0.999	−0.0123	1.000
27	252.21	0.0000	0.335	−0.0000	0.016	0.0002	0.023
28	259.60	−0.0000	0.013	0.0000	0.089	0.0000	0.028
29	275.50	−0.0027	0.998	0.0006	0.992	0.0061	0.999
30	292.34	0.0001	0.197	0.0001	0.154	0.0001	0.068
31	305.93	−0.0000	0.034	0.0001	0.206	0.0001	0.030
32	312.69	−0.0035	0.073	0.0035	0.073	0.0000	0.013
33	336.33	−0.0005	0.756	0.0005	0.594	−0.0000	0.009
34	339.75	0.0000	0.298	0.0001	0.999	0.0001	0.939
35	341.37	−0.0014	0.999	−0.0011	0.999	−0.0019	0.985
36	341.89	0.0021	0.999	0.0016	0.999	0.0028	0.999
37	387.97	0.0001	0.003	−0.0002	0.004	−0.0001	0.391
38	391.96	0.0002	0.867	0.0002	0.934	0.0005	0.744
39	420.35	0.0126	0.999	0.0136	1.000	0.0372	0.999
40	426.94	−0.0000	0.006	−0.0001	0.018	−0.0001	0.009

Table S29 | Linear fit for g-tensor for **2** with eq. 1 ($g_{ii} = (b(g_{ii}) * x) + g_{eq}$) for each component of the g-tensor (x, y, and z). The r^2 value is the coefficient of determination for each linear regression.

Mode	Frequency	b(g_{xx})	r^2 (g_{xx})	b(g_{yy})	r^2 (g_{yy})	b(g_{zz})	r^2 (g_{zz})
7	34.8	0.0000	0.946	−0.0002	−0.998	−0.0017	−0.999
8	35.3	0.0000	−0.970	0.0000	0.968	0.0003	0.998
9	85.3	0.0000	0.971	0.0000	0.052	0.0000	0.937
10	96.3	0.0000	0.999	0.0000	−1.000	−0.0003	−0.999
11	99.9	−0.0014	−1.000	−0.0008	−0.999	−0.0022	−0.997
12	105.2	−0.0000	−0.970	−0.0000	−0.9711	−0.0000	−0.995
13	129.3	0.0011	0.999	0.0018	0.999	0.0076	0.999
14	132.7	−0.0000	−0.953	−0.0000	−0.825	−0.0001	−0.956
15	139.3	−0.0030	−1.000	−0.0032	−0.999	−0.0131	−0.999
16	155.2	−0.0005	−1.000	−0.0005	−1.000	−0.0014	−1.000
17	179.2	0.0000	0.410	0.0000	−0.135	0.0000	0.475
18	191.3	−0.0010	−1.000	−0.0010	−1.000	−0.0039	−1.000
19	197.1	0.0000	0.204	−0.0000	−0.247	−0.0000	−0.327
20	211.2	0.0002	0.291	−0.0002	−0.290	0.0000	0.268
21	241.5	0.0014	0.999	0.0002	0.999	0.0067	0.999
22	260	0.0033	1.000	0.0041	1.000	0.0107	1.000
23	265	0.0000	0.108	−0.0001	−0.307	−0.0001	−0.357
24	272.1	0.0000	0.202	−0.0001	−0.277	−0.0001	−0.497
25	293.6	0.0000	−0.886	−0.0000	−0.425	−0.0000	−0.337
26	298.1	−0.0014	−0.999	−0.0011	−0.999	−0.0037	−0.999
27	331.9	0.0052	0.999	0.0058	0.999	0.0233	0.999
28	384	0.0000	0.729	−0.0000	−0.365	−0.0000	−0.298
29	399.1	0.0077	1.000	0.0084	1.000	0.0294	0.999
30	439.9	0.0001	0.354	0.0001	0.462	0.0003	0.376
31	447.2	−0.0076	−1.000	−0.0091	−0.999	−0.0253	−0.999

Table S30 | Linear fit for g-tensor for **3** with eq. 1 ($g_{ii} = (b(g_{ii}) * x) + g_{eq}$) for each component of the g-tensor (x, y, and z). The r^2 value is the coefficient of determination for each linear regression.

Mode	Frequency	$b(g_{xx})$	r^2 (g_{xx})	$b(g_{yy})$	r^2 (g_{yy})	$b(g_{zz})$	r^2 (g_{zz})
7	49.4	−0.0009	0.999	−0.0009	0.999	−0.0006	0.999
8	49.8	0.0000	0.760	0.0000	0.265	0.0000	0.097
9	69.4	0.0012	0.999	0.0004	0.999	−0.0036	0.999
10	78.8	0.0000	0.060	0.0000	0.000	0.0000	0.061
11	93.5	0.0000	0.080	0.0000	0.533	0.0000	0.071
12	101.9	0.0000	0.000	0.0000	0.003	0.0000	0.007
13	123.6	0.0000	0.043	0.0000	0.036	0.0000	0.031
14	150.6	0.0000	0.054	0.0000	0.055	0.0000	0.055
15	180.4	0.0000	0.064	0.0000	0.034	0.0000	0.026
16	185.6	−0.0004	0.998	0.0004	0.999	0.0042	0.999
17	190.7	0.0000	0.633	0.0000	0.057	−0.0002	0.044
18	199.3	0.0036	0.993	0.0044	0.997	0.0035	0.953
19	225.1	−0.0028	0.999	−0.0024	0.999	−0.0083	0.999
20	228.3	0.0000	0.018	0.0000	0.136	0.0000	0.051
21	230	0.0000	0.991	0.0000	0.958	0.0000	0.981
22	231.8	0.0012	1.000	0.0010	1.000	0.0031	1.000
23	233.1	0.0000	0.064	0.0000	0.065	0.0000	0.304
24	247.9	−0.0000	0.129	0.0000	0.046	−0.0000	0.016
25	251	0.0000	0.052	0.0000	0.053	0.0000	0.039
26	260.8	−0.0001	0.036	0.0001	0.052	0.0000	0.006
27	267.6	0.0000	0.039	0.0000	0.041	0.0000	0.543
28	309	0.0026	1.000	−0.0029	1.000	−0.0179	0.999
29	315.2	0.0000	0.072	0.0000	0.027	0.0000	0.025
30	316.6	−0.0000	0.055	0.0000	0.000	−0.0000	0.049
31	339.8	0.0000	0.06522	0.0000	0.076	0.0000	0.053
32	350.1	−0.0000	0.04305	−0.0000	0.047	−0.0001	0.043
33	368.3	0.0040	0.99978	0.0049	0.999	0.0202	0.999
34	380.2	−0.0000	0.05372	−0.0000	0.051	−0.0001	0.050
35	385	0.0062	1.00000	−0.0062	1.000	−0.0266	1.000
36	409.2	0.0000	0.11536	0.0000	0.000	−0.0000	0.010
37	465.7	−0.0000	0.05517	−0.0000	0.047	−0.0000	0.050
38	482.2	0.0020	0.99995	0.0016	0.999	0.0067	0.998
39	494.4	−0.0000	0.05013	0.0000	0.064	0.0000	0.041

Table S31 | Quadratic fit for *g*-tensor for **1** with eq. 2 ($g_{ii} = \left(a(g_{ii}) * x^2\right) + g_{eq}$) for each component of the *g*-tensor (x, y, and z). The r^2 value is the coefficient of determination for each quadratic regression.

Mode	Frequency	$a(g_{xx})$	$r^2\ (g_{xx})$	$a(g_{yy})$	$r^2\ (g_{yy})$	$a(g_{zz})$	$r^2\ (g_{zz})$
3	7.4	0.0003	0.399	0.0003	0.101	0.0007	0.141
4	14.77	0.0004	0.377	0.0003	0.829	0.0006	0.886
5	20.09	0.0003	0.717	0.0001	0.009	0.0004	0.139
6	38.38	−0.0063	0.524	0.0065	0.542	0.0008	0.702
7	40.67	0.0004	0.038	0.0003	0.208	0.0015	0.210
8	56.96	−0.0039	0.041	−0.0017	0.039	−0.0087	0.044
9	74.64	−0.0547	0.964	0.0558	0.962	0.0006	0.425
10	90.59	−0.0010	0.202	0.0010	0.179	−0.0006	0.345
11	112.12	0.0080	0.051	0.0011	0.066	−0.0135	0.048
12	115.41	−0.0033	0.042	−0.0002	0.021	0.0070	0.043
13	132.06	−0.0003	0.023	−0.0005	0.064	−0.0018	0.041
14	133.15	−0.0005	0.034	0.0007	0.034	0.0060	0.046
15	143.79	0.0009	0.073	0.0001	0.012	0.0008	0.026
16	145.55	0.0005	0.008	0.0005	0.114	0.0007	0.022
17	149.51	0.0291	0.043	0.0183	0.056	0.0560	0.046
18	158.63	−0.0007	0.112	0.0022	0.606	0.0062	0.951
19	161.12	−0.1748	0.991	0.1778	0.991	0.0002	0.116
20	188.66	0.0054	0.044	0.0021	0.038	−0.0060	0.052
21	189.18	−0.00037	0.66715	0.00057	0.09711	−0.00013	0.018
22	211.25	−0.39286	0.95859	−0.39128	0.95863	−0.01265	0.999
23	219.52	−0.00034	0.01482	−0.00056	0.03104	−0.00076	0.042
24	225.45	−0.02322	0.78371	0.02310	0.84847	−0.00061	0.071
25	227.33	−0.00205	0.33483	−0.00158	0.67685	−0.00235	0.720
26	230.58	−0.01769	0.02642	−0.01702	0.02206	−0.02788	0.026
27	252.21	0.00092	0.80081	0.00290	0.92047	0.01934	0.999
28	259.6	−0.00165	0.99807	−0.00071	0.80073	−0.00055	0.847
29	275.5	−0.00796	0.04241	0.00063	0.00519	0.01416	0.027
30	292.34	−0.00252	0.65894	−0.00183	0.70960	−0.00624	0.831
31	305.93	−0.00227	0.99922	−0.00139	0.65276	0.01017	0.999
32	312.69	−0.17799	0.96274	0.17951	0.96308	0.00539	0.997
33	336.33	−0.00500	0.38975	0.00659	0.56317	0.00125	0.931
34	339.75	−0.00012	0.50210	0.00015	0.03316	0.00033	0.134
35	341.37	−0.00272	0.01995	−0.00195	0.01605	−0.00098	0.001
36	341.89	0.00486	0.02715	0.00394	0.02985	0.00781	0.038
37	387.97	−0.03685	0.92937	0.03857	0.92293	0.00140	0.391
38	391.96	0.00128	0.30890	0.00123	0.21000	0.00579	0.462
39	420.35	0.03586	0.04311	0.03981	0.04578	0.10709	0.044
40	426.94	−0.00539	0.98097	−0.00513	0.99297	−0.01493	0.985

Table S32 | Quadratic fit for g-tensor for **2** with eq. 2 ($g_{ii} = (a(g_{ii}) * x^2) + g_{eq}$) for each component of the g-tensor (x, y, and z). The r^2 value is the coefficient of determination for each quadratic regression.

Mode	Frequency	$a(g_{xx})$	$r^2\ (g_{xx})$	$a(g_{yy})$	$r^2\ (g_{yy})$	$a(g_{zz})$	$r^2\ (g_{zz})$
7	34.8	0.0003	0.474	−0.0000	0.002	−0.0035	0.027
8	35.3	0.0000	0.001	0.0003	0.361	0.0016	0.115
9	85.3	−0.0000	0.049	0.0001	0.9447	−0.0000	0.303
10	96.3	0.0001	0.034	−0.0000	0.061	−0.0011	0.054
11	99.9	−0.0038	0.045	−0.0015	0.022	−0.0012	0.002
12	105.2	0.0000	0.080	0.0000	0.150	−0.0000	0.005
13	129.3	0.0042	0.067	0.0076	0.080	0.0267	0.059
14	132.7	0.0001	0.229	0.0003	0.776	0.0003	0.233
15	139.3	−0.0083	0.043	−0.0086	0.040	−0.0346	0.039
16	155.2	−0.0015	0.047	−0.0015	0.048	−0.0043	0.048
17	179.2	0.0001	0.927	0.0001	0.994	0.0003	0.905
18	191.3	−0.0031	0.043	−0.0030	0.046	−0.0117	0.047
19	197.1	−0.0033	0.998	0.0075	0.999	0.0038	0.999
20	211.2	−0.0204	0.999	0.0190	0.999	−0.0020	0.999
21	241.5	0.0042	0.037	0.0004	0.018	0.0245	0.056
22	260	0.0060	0.024	0.0075	0.026	0.0197	0.026
23	265	−0.0054	0.994	0.0114	0.999	0.0189	0.998
24	272.1	−0.0048	0.999	0.0052	0.999	0.0023	0.983
25	293.6	−0.000	0.563	0.0005	0.995	0.0016	0.999
26	298.1	−0.0049	0.065	−0.0030	0.044	−0.0105	0.045
27	331.9	0.0133	0.037	0.0141	0.033	0.0610	0.039
28	384	−0.0001	0.880	0.0011	0.998	0.0019	0.999
29	399.1	0.0226	0.045	0.0252	0.047	0.0909	0.050
30	439.9	−0.0097	0.998	−0.0031	0.990	−0.0203	0.997
31	447.2	−0.0213	0.042	−0.0252	0.041	−0.0651	0.036

Table S33 | Quadratic fit for g-tensor for **3** with eq. 2 ($g_{ii} = (a(g_{ii}) * x^2) + g_{eq}$) for each component of the g-tensor (x, y, and z). The r^2 value is the coefficient of determination for each quadratic regression.

Mode	Frequency	$a(g_{xx})$	r^2 (g_{xx})	$a(g_{yy})$	r^2 (g_{yy})	$a(g_{zz})$	r^2 (g_{zz})
7	49.4	−0.0009	1.000	−0.0009	1.000	−0.0006	0.999
8	49.8	0.0000	0.998	0.0000	0.999	0.0000	0.999
9	69.4	0.0012	1.000	0.0004	1.000	−0.0036	1.000
10	78.8	0.0000	0.993	0.0000	0.880	0.0000	0.992
11	93.5	0.0000	0.882	0.0000	0.860	0.0000	0.999
12	101.9	0.0000	0.910	0.0000	0.944	−0.0000	0.999
13	123.6	0.0000	0.999	0.0000	0.999	0.0000	0.999
14	150.6	0.0000	0.999	0.0000	0.999	0.0000	0.999
15	180.4	0.0000	0.995	0.0000	0.997	0.0000	0.990
16	185.6	−0.0004	1.000	0.0004	1.000	0.0042	1.000
17	190.7	0.0000	0.995	0.0000	0.999	0.0000	1.000
18	199.3	0.0035	1.000	0.0043	1.000	0.0033	1.000
19	225.1	−0.0029	1.000	−0.0024	1.000	−0.0084	1.000
20	228.3	0.0000	0.970	0.0000	0.861	0.0000	0.985
21	230	0.0000	0.999	0.0000	0.998	0.0000	0.999
22	231.8	0.0012	1.000	0.0010	1.000	0.0031	1.000
23	233.1	0.0000	0.963	0.0000	0.921	0.0000	0.923
24	247.9	0.0000	0.979	0.0000	0.999	0.0000	0.999
25	251	0.0000	0.999	0.0000	0.999	0.0000	0.999
26	260.8	0.0000	0.999	0.0000	0.999	0.0000	0.999
27	267.6	0.0000	0.999	0.0000	0.999	0.0000	0.755
28	309	−0.0026	1.00000	−0.0029	1.000	−0.0179	1.000
29	315.2	0.0000	0.99667	0.0000	0.999	−0.0000	0.999
30	316.6	0.0000	0.99541	0.0000	0.000	0.0000	0.999
31	339.8	0.0000	0.13022	0.0000	0.999	0.0000	0.999
32	350.1	0.0000	0.99972	0.0000	0.999	0.0000	0.999
33	368.3	0.0040	1.00000	0.0049	1.000	0.0202	1.000
34	380.2	0.0000	0.99979	0.0000	0.999	0.0000	0.999
35	385	−0.0062	1.00000	−0.0062	1.000	−0.0266	1.000
36	409.2	0.0000	0.99707	0.0000	0.996	0.0000	0.999
37	465.7	0.0000	0.99997	0.0000	0.999	0.0000	0.999
38	482.2	0.0020	1.00000	0.0016	1.000	0.0067	1.000
39	494.4	0.0000	0.99985	0.0000	0.963	0.0000	0.999

Table S34 | Linear fit for Cu A-tensor for **1** with eq. 2 ($A_{ii} = (a(A_{ii}) * x) + A_{eq}$) for each component of the A-tensor (x, y, and z). The r^2 value is the coefficient of determination for each linear regression.

Mode	Frequency	b(A_{xx})	r^2 (A_{xx})	b(A_{yy})	r^2 (A_{yy})	b(A_{zz})	r^2 (A_{zz})
3	7.40	−155.02	0.157	155.14	0.157	−0.04	0.348
4	14.77	−155.14	0.157	154.78	0.156	0.23	0.959
5	20.09	−155.07	0.157	154.71	0.156	0.23	0.799
6	38.38	−155.43	0.157	155.33	0.157	0.09	0.348
7	40.67	−154.64	0.156	155.75	0.158	−0.77	0.978
8	56.96	−158.18	0.162	147.98	0.145	5.90	1.000
9	74.64	−154.78	0.155	154.54	0.154	0.07	0.011
10	90.59	−154.92	0.156	155.33	0.157	−0.37	0.811
11	112.12	−140.29	0.132	177.05	0.195	−29.68	1.000
12	115.41	−162.42	0.169	144.21	0.138	14.90	1.000
13	132.06	−155.92	0.158	153.78	0.154	0.50	0.958
14	133.15	−152.21	0.152	158.25	0.162	0.45	0.914
15	143.79	−155.02	0.157	155.38	0.157	−0.18	0.628
16	145.55	−154.97	0.156	155.21	0.157	−0.05	0.136
17	149.51	−156.98	0.160	169.61	0.182	−3.96	0.983
18	158.63	−155.37	0.157	154.70	0.156	0.00	0.000
19	161.12	−155.41	0.152	155.28	0.152	−0.07	0.002
20	188.66	−146.92	0.143	165.16	0.174	−21.18	1.000
21	189.18	−155.38	0.157	154.57	0.156	0.87	0.903
22	211.25	−98.16	0.055	108.43	0.055	−5.86	0.038
23	219.52	−95.95	0.054	94.08	0.052	1.50	0.996
24	225.45	−95.90	0.054	95.27	0.053	0.45	0.810
25	227.33	−94.72	0.053	95.18	0.054	−0.34	0.406
26	230.58	−126.08	0.090	65.48	0.026	49.34	1.000
27	252.21	−94.98	0.053	94.73	0.053	−0.46	0.012
28	259.60	−94.95	0.053	95.18	0.053	−0.26	0.129
29	275.50	−117.33	0.079	63.92	0.025	51.03	1.000
30	292.34	−94.92	0.053	95.67	0.054	−0.81	0.717
31	305.93	−95.27	0.053	95.12	0.053	−0.08	0.008
32	312.69	−97.72	0.055	97.49	0.055	−0.18	0.047
33	336.33	−95.52	0.054	95.11	0.053	0.14	0.045
34	339.75	−95.03	0.053	95.12	0.053	−0.04	0.080
35	341.37	−98.81	0.057	91.58	0.050	1.70	0.963
36	341.89	−89.50	0.048	100.19	0.059	−2.56	0.992
37	387.97	−154.50	0.155	154.84	0.156	0.01	0.007
38	391.96	−155.07	0.157	155.02	0.156	−0.26	0.088
39	420.35	−158.55	0.162	156.53	0.159	−23.18	1.000
40	426.94	−154.79	0.157	154.73	0.157	0.13	0.019

Table S35 | Linear fit for Cu A-tensor for **2** with eq. 2 ($A_{ii} = (a(A_{ii}) * x) + A_{eq}$) for each component of the A-tensor (x, y, and z). The r^2 value is the coefficient of determination for each linear regression.

Mode	Frequency	$b(A_{xx})$	$r^2\ (A_{xx})$	$b(A_{yy})$	$r^2\ (A_{yy})$	$b(A_{zz})$	$r^2\ (A_{zz})$
7	34.8	2.93	0.998	2.74	1.000	5.74	0.999
8	35.3	−0.63	0.921	−0.52	0.958	−1.08	0.997
9	85.3	0.04	0.348	0.03	0.065	0.00	nan
10	96.3	0.41	0.939	0.47	0.983	0.99	0.996
11	99.9	−1.63	0.973	−1.83	0.997	1.89	0.895
12	105.2	−0.03	0.065	0.04	0.348	0.10	0.543
13	129.3	−10.11	0.991	−8.93	0.993	−17.63	0.990
14	132.7	0.01	0.004	0.11	0.272	0.01	0.002
15	139.3	1.32	0.957	0.28	0.735	12.44	0.997
16	155.2	0.39	0.935	0.34	0.950	−0.04	0.345
17	179.2	−0.06	0.141	0.00	nan	−0.04	0.340
18	191.3	1.19	0.996	0.71	0.989	3.34	1.000
19	197.1	−1.72	0.056	1.32	0.059	−0.38	0.042
20	211.2	−7.52	0.027	1.43	0.045	−3.27	0.024
21	241.5	−6.54	0.990	3.56	0.955	−9.46	0.998
22	260	0.85	0.971	2.28	0.999	−11.56	1.000
23	265	0.06	0.022	−0.20	0.032	−0.66	0.046
24	272.1	−1.38	0.026	1.12	0.026	−0.25	0.018
25	293.6	−0.60	0.076	−0.32	0.050	−0.55	0.053
26	298.1	12.60	1.000	11.22	1.000	7.35	0.991
27	331.9	8.66	0.898	−11.40	0.988	−12.48	1.000
28	384	−0.62	0.047	0.03	0.069	−0.33	0.050
29	399.1	9.40	0.999	8.15	0.998	−30.42	0.999
30	439.9	0.20	0.041	−0.28	0.026	0.09	0.019
31	447.2	−14.28	1.000	−15.46	1.000	16.44	0.995

Table S36 | Linear fit for Cu A-tensor for **3** with eq. 2 ($A_{ii} = (a(A_{ii}) * x) + A_{eq}$) for each component of the A-tensor (x, y, and z). The r^2 value is the coefficient of determination for each linear regression.

Mode	Frequency	b(A_{xx})	r^2 (A_{xx})	b(A_{yy})	r^2 (A_{yy})	b(A_{zz})	r^2 (A_{zz})
7	49.4	0.04	0.348	0.09	0.696	−2.08	0.999
8	49.8	0.00	0.000	0.00	nan	0.00	0.000
9	69.4	3.88	0.999	3.46	0.999	9.62	0.999
10	78.8	−0.03	0.065	0.00	nan	0.00	nan
11	93.5	0.00	nan	0.00	nan	−0.03	0.065
12	101.9	0.10	0.009	0.00	nan	0.07	0.011
13	123.6	−0.03	0.065	0.00	nan	−0.03	0.022
14	150.6	−0.07	0.071	−0.03	0.022	−0.13	0.104
15	180.4	0.00	nan	0.00	nan	−0.03	0.022
16	185.6	−13.61	1.000	−11.21	0.999	−17.87	0.999
17	190.7	−0.80	0.022	0.33	0.013	−0.97	0.047
18	199.3	9.60	0.998	9.42	0.997	23.30	0.996
19	225.1	0.98	0.973	4.68	0.998	10.18	0.999
20	228.3	0.00	nan	0.00	nan	0.00	nan
21	230	0.14	0.019	−0.01	0.002	0.05	0.026
22	231.8	1.26	0.999	0.42	0.981	−0.14	0.918
23	233.1	0.00	nan	0.00	nan	0.00	nan
24	247.9	0.14	0.023	−0.01	0.001	0.05	0.001
25	251	−0.04	0.019	0.03	0.065	−0.03	0.022
26	260.8	−10.46	0.043	2.08	0.448	−4.45	0.029
27	267.6	0.03	0.022	−0.03	0.022	−0.20	0.041
28	309	−1.36	0.998	−2.71	0.999	10.68	0.999
29	315.2	0.00	nan	0.00	0.000	−0.04	0.022
30	316.6	0.04	0.010	−0.20	0.038	−0.06	0.027
31	339.8	0.09	0.178	−0.10	0.038	−0.06	0.027
32	350.1	−0.54	0.060	0.19	0.044	−0.14	0.038
33	368.3	14.04	0.999	12.86	0.999	5.04	0.989
34	380.2	0.00	0.000	0.11	0.060	−0.04	0.010
35	385	−0.86	0.989	−5.10	0.999	11.83	0.998
36	409.2	−0.03	0.026	−0.10	0.418	−0.18	0.073
37	465.7	−0.06	0.071	0.03	0.065	−0.08	0.038
38	482.2	−15.82	0.999	−14.23	0.999	−16.08	0.999
39	494.4	0.03	0.022	0.08	0.060	−0.12	0.032

Table S37 | Quadratic fit of Cu A-tensor for **1** with eq. 2 ($A_{ii} = \left(a(A_{ii}) * x^2\right) + A_{eq}$) for each component of the A-tensor (x, y, and z). The r^2 value is the coefficient of determination for each quadratic regression.

Mode	Frequency	b(A_{xx})	r^2 (A_{xx})	b(A_{yy})	r^2 (A_{yy})	b(A_{zz})	r^2 (A_{zz})
3	7.4	−4481.23	0.694	4481.598	0.694	−0.65574	0.420
4	14.77	−4481.6	0.694	4480.656	0.694	0.532787	0.028
5	20.09	−4480.57	0.694	4480.943	0.694	−0.77869	0.049
6	38.38	−4485.33	0.694	4486.475	0.694	−1.31148	0.420
7	40.67	−4480.12	0.694	4483.238	0.693	−3.72951	0.121
8	56.96	−4489.8	0.692	4461.23	0.697	16.51639	0.042
9	74.64	−4506.72	0.697	4514.918	0.698	−7.95082	0.894
10	90.59	−4480.04	0.694	4482.787	0.694	−3.44262	0.373
11	112.12	−4436.84	0.700	4547.828	0.682	−90.6147	0.049
12	115.41	−4502.17	0.690	4449.959	0.699	41.76231	0.042
13	132.06	−4482.99	0.693	4477.705	0.694	0.163934	0.001
14	133.15	−4472.25	0.695	4490.615	0.692	−0.36885	0.003
15	143.79	−4481.23	0.694	4482.172	0.694	-2.2541	0.496
16	145.55	−4480.7	0.694	4482.418	0.694	−1.84426	0.830
17	149.51	−4489.1	0.693	4522.705	0.686	−18.6066	0.115
18	158.63	−4480.82	0.694	4483.278	0.694	−7.45902	0.953
19	161.12	−4589.67	0.705	4599.262	0.706	−18.2787	0.971
20	188.66	−4456.43	0.697	4511.27	0.689	−65.5328	0.051
21	189.18	−4482.17	0.694	4479.099	0.694	−1.22951	0.010
22	211.25	−4840.18	0.672	5592.852	0.732	−425.799	0.999
23	219.52	−4748.12	0.666	4744.169	0.666	2.076677	0.010
24	225.45	−4754.39	0.666	4755.391	0.666	−1.95687	0.077
25	227.33	−4743.21	0.665	4738.338	0.665	4.872204	0.417
26	230.58	−4817.01	0.659	4674.92	0.665	104.6326	0.023
27	252.21	−4731.63	0.664	4725.759	0.664	−58.3866	0.998
28	259.6	−4744.61	0.665	4743.69	0.665	−9.98403	0.951
29	275.5	−4799.88	0.662	4672.364	0.665	104.6725	0.021
30	292.34	−4762.22	0.667	4755.95	0.666	5.071885	0.141
31	305.93	−4762.02	0.667	4758.706	0.667	−12.9393	0.992
32	312.69	−4855.27	0.675	4840.295	0.674	−11.5815	0.981
33	336.33	−4742.65	0.665	4756.27	0.667	−8.70607	0.863
34	339.75	−4745.73	0.666	4745.767	0.666	−1.67732	0.704
35	341.37	−4754.59	0.665	4738.578	0.666	−0.79872	0.001
36	341.89	−4733.07	0.666	4757.947	0.665	−8.70607	0.057
37	387.97	−4494.51	0.696	4494.959	0.695	−0.86066	0.241
38	391.96	−4480.57	0.694	4482.541	0.694	−12.0492	0.991
39	420.35	−4495.86	0.693	4480.697	0.692	−75.2049	0.056
40	426.94	−4463.4	0.692	4466.189	0.692	12.95082	0.992

Table S38 | Quadratic fit for Cu A-tensor data for **2** with eq. 2 ($A_{ii} = \left(a(A_{ii}) * x^2\right) + A_{eq}$) for each component of the A-tensor (x, y, and z). The r^2 value is the coefficient of determination for each quadratic regression.

Mode	Frequency	b(A_{xx})	r^2 (A_{xx})	b(A_{yy})	r^2 (A_{yy})	b(A_{zz})	r^2 (A_{zz})
7	34.8	6.60	0.032	7.38	0.046	13.60	0.036
8	35.3	−4.28	0.213	−0.17	0.001	−3.85	0.063
9	85.3	−0.57	0.420	1.45	0.919	0.00	nan
10	96.3	0.26	0.002	2.29	0.113	3.87	0.075
11	99.9	−7.67	0.137	−6.02	0.069	−2.81	0.013
12	105.2	−1.48	0.919	0.58	0.420	1.08	0.369
13	129.3	−44.13	0.092	−37.78	0.087	−79.11	0.098
14	132.7	−2.18	0.888	2.37	0.788	−3.41	0.928
15	139.3	0.19	0.000	−1.32	0.095	26.56	0.025
16	155.2	0.26	0.002	1.76	0.140	−0.65	0.416
17	179.2	−1.90	0.842	0.00	nan	−0.67	0.412
18	191.3	2.78	0.028	2.83	0.079	10.23	0.048
19	197.1	−96.28	0.999	71.68	0.999	−24.45	0.999
20	211.2	−657.36	0.998	91.43	0.887	−305.66	0.999
21	241.5	−11.58	0.013	23.25	0.173	−36.89	0.064
22	260	0.08	0.000	4.65	0.032	−23.17	0.031
23	265	8.56	0.971	−26.16	0.994	−71.56	1.000
24	272.1	−110.23	0.998	89.06	0.997	−24.07	0.998
25	293.6	−28.35	0.997	−18.46	0.998	−31.23	0.999
26	298.1	37.15	0.049	34.44	0.054	13.52	0.019
27	331.9	−13.26	0.012	−16.47	0.011	−37.41	0.050
28	384	−38.69	0.999	1.62	0.926	−19.72	0.998
29	399.1	31.53	0.059	28.87	0.066	−100.19	0.057
30	439.9	13.73	0.998	−23.35	0.996	8.37	0.985
31	447.2	−39.15	0.041	−41.05	0.038	31.41	0.020

Table S39 | Quadratic fit for Cu A-tensor data for **3** with eq. 2 ($A_{ii} = \left(a(A_{ii}) * x^2\right) + A_{eq}$) for each component of the A-tensor (x, y, and z). The r^2 value is the coefficient of determination for each quadratic regression.

Mode	Frequency	$a(A_{xx})$	r^2 (A_{xx})	$a(A_{yy})$	r^2 (A_{yy})	$a(A_{zz})$	r^2 (A_{zz})
7	49.4	0.04	0.348	0.09	0.696	−2.08	0.999
8	49.8	0.00	0.000	0.00	nan	0.00	0.000
9	69.4	3.88	0.999	3.46	0.999	9.62	1.000
10	78.8	−0.03	0.065	0.00	nan	0.00	nan
11	93.5	0.00	nan	0.00	nan	−0.03	0.065
12	101.9	0.10	0.009	0.00	nan	0.07	0.011
13	123.6	−0.03	0.065	0.00	nan	−0.03	0.022
14	150.6	−0.07	0.071	−0.03	0.022	−0.13	0.104
15	180.4	0.00	nan	0.00	nan	−0.03	0.022
16	185.6	−13.61	1.000	−11.21	1.000	−17.87	1.000
17	190.7	−0.80	0.022	0.33	0.013	−0.97	0.047
18	199.3	9.60	0.998	9.42	0.999	23.30	0.996
19	225.1	0.98	0.973	4.68	0.998	10.18	1.000
20	228.3	0.00	nan	0.00	nan	0.00	nan
21	230	0.14	0.019	−0.01	0.002	0.05	0.026
22	231.8	1.26	0.999	0.42	0.981	−0.14	0.918
23	233.1	0.00	nan	0.00	nan	0.00	nan
24	247.9	0.14	0.023	−0.01	0.001	0.05	0.001
25	251	−0.04	0.019	0.03	0.065	−0.03	0.022
26	260.8	−10.46	0.043	2.08	0.448	−4.45	0.029
27	267.6	0.03	0.022	−0.03	0.022	−0.20	0.041
28	309	−1.36	0.998	−2.71	1.000	10.68	1.000
29	315.2	0.00	nan	0.00	0.000	−0.04	0.022
30	316.6	0.04	0.010	−0.20	0.038	−0.06	0.027
31	339.8	0.09	0.178	−0.10	0.038	−0.06	0.027
32	350.1	−0.54	0.060	0.19	0.044	−0.14	0.038
33	368.3	14.04	1.000	12.86	1.000	5.04	0.989
34	380.2	0.00	0.000	0.11	0.060	−0.04	0.010
35	385	−0.86	0.989	−5.10	0.999	11.83	0.998
36	409.2	−0.03	0.026	−0.10	0.418	−0.18	0.073
37	465.7	−0.06	0.071	0.03	0.065	−0.08	0.038
38	482.2	−15.82	1.000	−14.23	1.000	−16.08	0.999
39	494.4	0.03	0.022	0.08	0.060	−0.12	0.032

Table S40 | Linear fit for N A-tensor data for **1** with eq. 2 ($A_{ii} = (a(A_{ii}) * x) + A_{eq}$) for each component of the A-tensor (x, y, and z). The r^2 value is the coefficient of determination for each linear regression.

Mode	Frequency	b(A_{xx})	r^2 (A_{xx})	b(A_{yy})	r^2 (A_{yy})	b(A_{zz})	r^2 (A_{zz})
3	7.40	0.02	0.532	−0.02	0.261	−0.02	0.696
4	14.77	0.01	0.196	0.01	0.098	0.02	0.464
5	20.09	0.04	0.918	0.01	0.348	0.02	0.696
6	38.38	0.02	0.087	0.02	0.533	0.04	0.852
7	40.67	−0.06	0.957	−0.04	0.795	−0.049	0.880
8	56.96	0.59	0.999	0.52	0.997	0.59	0.999
9	74.64	0.01	0.196	0.00	0.000	−0.00	0.011
10	90.59	−0.05	0.898	−0.04	0.609	−0.02	0.533
11	112.12	−3.07	0.999	−3.12	0.999	−2.81	0.999
12	115.41	1.57	0.999	1.61	0.999	1.41	0.999
13	132.06	0.10	0.960	0.10	0.960	0.17	0.986
14	133.15	−0.13	0.985	−0.13	0.900	−0.42	0.999
15	143.79	−0.02	0.696	−0.02	0.464	−0.01	0.098
16	145.55	−0.02	0.533	0.00	0.007	−0.01	0.181
17	149.51	−0.20	0.951	−0.23	0.990	−0.40	0.995
18	158.63	0.016	0.391	0.02	0.199	0.03	0.725
19	161.12	−0.010	0.348	−0.02	0.464	0.01	0.272
20	188.66	−2.69	0.999	−2.75	0.999	−2.13	0.999
21	189.18	0.12	0.935	0.11	0.914	0.09	0.890
22	211.25	0.03	0.480	0.05	0.735	0.03	0.403
23	219.52	0.15	0.975	0.17	0.984	0.14	0.985
24	225.45	0.05	0.903	0.06	0.781	0.05	0.903
25	227.33	−0.03	0.483	−0.03	0.807	−0.03	0.320
26	230.58	4.62	0.999	4.68	0.999	4.45	0.999
27	252.21	−0.05	0.035	−0.03	0.012	−0.01	0.004
28	259.60	−0.04	0.074	−0.04	0.099	−0.03	0.097
29	275.50	8.03	0.999	8.16	0.999	6.90	0.999
30	292.34	−0.15	0.657	−0.14	0.720	−0.13	0.733
31	305.93	−0.01	0.002	−0.02	0.004	0.02	0.008
32	312.69	−0.01	0.007	−0.00	0.001	0.03	0.095
33	336.33	0.04	0.784	0.02	0.360	−0.01	0.080
34	339.75	0.00	nan	−0.01	0.040	−0.01	0.250
35	341.37	−0.59	0.997	−0.62	0.995	−0.43	0.993
36	341.89	1.04	0.999	1.06	0.999	0.79	0.999
37	387.97	0.03	0.438	0.01	0.043	0.05	0.785
38	391.96	−0.07	0.880	−0.08	0.932	−0.06	0.809
39	420.35	−6.25	0.999	−6.34	0.999	−5.73	0.999
40	426.94	−0.00	0.007	0.00	nan	0.03	0.543

Table S41 | Linear fit for N A-tensor data for **2** with eq. 2 ($A_{ii} = (a(A_{ii}) * x) + A_{eq}$) for each component of the A-tensor (x, y, and z). The r^2 value is the coefficient of determination for each linear regression.

Mode	Frequency	$b(A_{xx})$	$r^2\ (A_{xx})$	$b(A_{yy})$	$r^2\ (A_{yy})$	$b(A_{zz})$	$r^2\ (A_{zz})$
7	34.8	0.21	0.983	0.19	0.848	0.28	0.988
8	35.3	−0.04	0.696	−0.07	0.918	−0.01	0.022
9	85.3	0.00	nan	−0.01	0.087	0.00	nan
10	96.3	−0.06	0.877	0.00	nan	0.00	nan
11	99.9	−0.75	0.999	−0.87	0.996	−1.14	0.998
12	105.2	−0.02	0.348	−0.03	0.272	0.00	nan
13	129.3	0.72	0.859	0.90	0.902	0.83	0.883
14	132.7	−0.02	0.022	−0.05	0.117	−0.03	0.054
15	139.3	−2.27	0.998	−2.21	0.997	−2.40	0.998
16	155.2	−0.38	0.995	−0.39	0.990	−0.36	0.986
17	179.2	0.00	nan	0.02	0.340	0.00	nan
18	191.3	0.26	1.000	0.23	0.959	0.33	0.996
19	197.1	−0.04	0.533	−0.02	0.065	0.00	nan
20	211.2	−0.02	0.180	−0.02	0.080	0.00	nan
21	241.5	−3.82	0.998	−4.03	0.999	−3.59	0.997
22	260	−3.29	1.000	−3.26	1.000	−3.17	1.000
23	265	0.00	nan	−0.01	0.007	0.00	nan
24	272.1	0.01	0.029	−0.01	0.006	0.00	0.000
25	293.6	−0.02	0.340	0.00	0.000	0.00	nan
26	298.1	−1.12	0.980	−1.46	0.984	−0.86	0.969
27	331.9	−1.13	0.954	−1.31	0.953	−3.83	0.995
28	384	−0.02	0.340	−0.02	0.050	0.01	0.039
29	399.1	1.39	0.998	1.51	1.000	3.72	1.000
30	439.9	−0.03	0.026	−0.04	0.140	−0.03	0.050
31	447.2	17.41	1.000	17.73	1.000	19.55	1.000

Table S42 | Linear fit for N *A*-tensor data for **3** with eq. 2 ($A_{ii} = (a(A_{ii}) * x) + A_{eq}$) for each component of the *A*-tensor (x, y, and z). The r^2 value is the coefficient of determination for each linear regression.

Mode	*Frequency*	*b(A_{xx})*	*r^2 (A_{xx})*	*b(A_{yy})*	*r^2 (A_{yy})*	*b(A_{zz})*	*r^2 (A_{zz})*
7	49.4	−0.64	0.996	−0.67	0.999	−0.64	0.999
8	49.8	−0.03	0.543	0.00	0.000	0.00	0.003
9	69.4	0.34	0.986	0.46	0.999	0.66	0.999
10	78.8	−0.01	0.136	−0.02	0.587	−0.01	0.348
11	93.5	−0.01	0.136	0.01	0.348	0.00	nan
12	101.9	−0.02	0.157	−0.01	0.070	−0.02	0.199
13	123.6	−0.01	0.022	−0.01	0.065	−0.02	0.139
14	150.6	0.04	0.852	0.01	0.196	−0.02	0.278
15	180.4	−0.01	0.070	−0.02	0.532	−0.01	0.065
16	185.6	−0.34	0.980	−0.49	0.999	−0.99	0.999
17	190.7	−0.20	0.067	−0.19	0.064	−0.15	0.049
18	199.3	0.90	0.996	1.21	0.999	1.08	0.999
19	225.1	0.77	0.997	0.78	0.995	1.01	0.999
20	228.3	0.01	0.022	0.00	0.157	−0.01	0.098
21	230	0.00	0.000	0.01	0.348	−0.02	0.293
22	231.8	−0.42	0.994	−0.39	0.998	−0.40	0.996
23	233.1	−0.03	0.783	0.00	0.157	−0.00	0.004
24	247.9	−0.00	0.004	−0.02	0.032	−0.01	0.018
25	251	−0.02	0.073	−0.01	0.027	−0.01	0.018
26	260.8	−0.03	0.051	−0.03	0.054	−0.04	0.087
27	267.6	−0.03	0.051	−0.03	0.044	−0.04	0.066
28	309	2.48	0.999	2.48	0.999	2.52	0.999
29	315.2	0.00	0.000	−0.00	0.007	−0.02	0.391
30	316.6	−0.06	0.414	−0.03	0.126	−0.03	0.084
31	339.8	−0.04	0.328	−0.01	0.087	−0.02	0.107
32	350.1	−0.07	0.161	−0.05	0.078	−0.03	0.015
33	368.3	−3.76	0.999	−3.40	0.999	−3.34	0.999
34	380.2	−0.02	0.355	−0.02	0.261	−0.01	0.065
35	385	2.96	0.999	2.99	0.999	2.84	0.999
36	409.2	−0.03	0.060	−0.01	0.022	−0.02	0.033
37	465.7	−0.00	0.001	−0.01	0.029	−0.02	0.107
38	482.2	3.44	0.999	3.20	0.99884	2.07582	0.997
39	494.4	0.03	0.029	0.05	0.05280	0.05310	0.0654

Table S43 | Quadratic fit for N A-tensor for **1** with eq. 2 ($A_{ii} = (a(A_{ii}) * x^2) + A_{eq}$) for each component of the A-tensor (x, y, and z). The r^2 value is the coefficient of determination for each quadratic regression.

Mode	Frequency	b(A_{xx})	r^2 (A_{xx})	b(A_{yy})	r^2 (A_{yy})	b(A_{zz})	r^2 (A_{zz})
3	7.4	0.26	0.512	−0.14	0.107	0.00	0.000
4	14.77	0.09	0.133	−0.17	0.237	0.27	0.369
5	20.09	0.13	0.070	−0.16	0.420	0.00	0.000
6	38.38	0.51	0.455	−0.07	0.040	0.14	0.064
7	40.67	−0.14	0.029	−0.23	0.113	0.02	0.001
8	56.96	1.70	0.045	1.39	0.039	1.71	0.045
9	74.64	0.09	0.133	−0.27	0.369	0.26	0.512
10	90.59	−0.30	0.197	−0.47	0.496	−0.26	0.512
11	112.12	−9.41	0.050	−9.44	0.048	−8.31	0.046
12	115.41	4.61	0.046	4.58	0.043	4.20	0.047
13	132.06	0.40	0.080	0.07	0.003	0.30	0.017
14	133.15	−0.46	0.063	−0.71	0.135	−1.40	0.059
15	143.79	0.00	0.000	−0.27	0.370	0.17	0.237
16	145.55	−0.26	0.512	−0.22	0.241	−0.22	0.241
17	149.51	−0.25	0.007	−0.80	0.064	−0.87	0.024
18	158.63	−0.12	0.118	−0.29	0.184	0.04	0.009
19	161.12	0.16	0.420	−0.27	0.369	0.13	0.139
20	188.66	−8.27	0.050	−8.37	0.049	−6.40	0.048
21	189.18	−0.05	0.001	−0.09	0.003	−0.10	0.007
22	211.25	−0.32	0.273	−0.27	0.097	−0.35	0.326
23	219.52	0.44	0.044	0.18	0.006	0.21	0.011
24	225.45	−0.07	0.001	−0.27	0.073	0.25	0.125
25	227.33	0.31	0.219	−0.09	0.043	0.48	0.409
26	230.58	10.33	0.025	10.46	0.025	10.37	0.027
27	252.21	−3.94	0.997	−4.10343	0.99573	−2.82548	0.984
28	259.6	−1.80	0.977	−1.75719	0.95159	−1.42772	0.933
29	275.5	16.57	0.021	16.92293	0.02155	14.37700	0.022
30	292.34	−1.69	0.430	−1.46765	0.41094	−1.30791	0.386
31	305.93	−3.41	0.968	−3.37460	0.97074	−3.07508	0.935
32	312.69	−0.78	0.884	−1.02835	0.89196	−0.99840	0.761
33	336.33	0.06	0.012	0.11981	0.11502	0.03994	0.026
34	339.75	0.00	nan	−0.19968	0.31949	−0.12979	0.135
35	341.37	−0.86	0.011	−0.91853	0.01094	−0.51917	0.007
36	341.89	2.45	0.028	2.22644	0.02197	1.81709	0.027
37	387.97	0.01	0.000	−0.20492	0.32787	0.23566	0.108
38	391.96	−0.03	0.001	−0.14344	0.01530	0.18443	0.0408
39	420.35	17.16	0.040	17.72541	0.04142	15.75820	0.040
40	426.94	0.32	0.525	0.00000	nan	0.30738	0.369

Table S44 | Quadratic fit for N A-tensor for **2** with eq. 2 ($A_{ii} = \left(a(A_{ii}) * x^2\right) + A_{eq}$) for each component of the A-tensor (x, y, and z). The r^2 value is the coefficient of determination for each quadratic regression.

Mode	Frequency	b(A_{xx})	r^2 (A_{xx})	b(A_{yy})	r^2 (A_{yy})	b(A_{zz})	r^2 (A_{zz})
7	34.8	0.34	0.017	−0.38	0.021	0.68	0.038
8	35.3	0.00	0.000	−0.28	0.069	−0.02	0.002
9	85.3	0.00	nan	−0.07	0.026	0.00	nan
10	96.3	−0.20	0.044	0.00	nan	0.00	nan
11	99.9	−2.07	0.048	−3.01	0.075	−3.63	0.064
12	105.2	0.29	0.419	−0.23	0.139	0.00	nan
13	129.3	−1.84	0.028	−1.35	0.010	−1.64	0.017
14	132.7	−1.40	0.971	−1.78	0.945	−1.47	0.972
15	139.3	−7.96	0.068	−7.73	0.068	−8.39	0.068
16	155.2	−1.20	0.054	−1.31	0.058	−1.53	0.096
17	179.2	0.00	nan	−0.33	0.411	0.00	nan
18	191.3	0.77	0.046	0.56	0.028	1.05	0.051
19	197.1	−0.48	0.512	−0.78	0.919	0.00	nan
20	211.2	−0.19	0.129	−0.876	0.704	0.00	nan
21	241.5	10.08	0.029	11.37	0.034	−9.00	0.026
22	260	−5.95	0.025	−6.25	0.028	−5.65	0.024
23	265	0.00	nan	−1.94	0.595	0.00	nan
24	272.1	−1.01	0.862	−1.28	0.892	−0.66	0.658
25	293.6	−0.29	0.409	−1.07	0.785	0.00	nan
26	298.1	−5.45	0.132	−6.89	0.125	−4.55	0.154
27	331.9	−6.53	0.176	−7.55	0.175	14.51	0.079
28	384	−0.31	0.409	−0.73	0.557	−0.40	0.331
29	399.1	4.86	0.064	4.49	0.046	11.80	0.053
30	439.9	2.12	0.862	1.29	0.673	1.47	0.795
31	447.2	55.55	0.055	56.03	0.054	62.02	0.054

Table S45 | Quadratic fit for N A-tensor for **3** with eq. 2 ($A_{ii} = \left(a(A_{ii}) * x^2\right) + A_{eq}$) for each component of the A-tensor (x, y, and z). The r^2 value is the coefficient of determination for each quadratic regression.

Mode	Frequency	b(A_{xx})	r^2 (A_{xx})	b(A_{yy})	r^2 (A_{yy})	b(A_{zz})	r^2 (A_{zz})
7	49.4	−0.6	0.997	−0.67	0.999	−0.634	0.999
8	49.8	−0.02	0.755	0.00	0.387	0.012	0.713
9	69.4	0.34	0.987	0.46	0.999	0.663	0.999
10	78.8	-0.01	0.861	−0.03	0.608	−0.009	0.632
11	93.5	−0.01	0.861	0.01	0.632	0.000	nan
12	101.9	−0.01	0.743	−0.00	0.762	−0.013	0.856
13	123.6	0.00	0.656	−0.00	0.921	−0.013	0.531
14	150.6	0.04	0.855	0.01	0.272	−0.019	0.363
15	180.4	−0.00	0.644	−0.02	0.860	−0.002	0.921
16	185.6	−0.33	0.991	−0.49	0.999	−0.994	0.999
17	190.7	−0.04	0.999	−0.03	0.999	−0.005	0.999
18	199.3	0.91	0.999	1.21	0.999	1.089	0.999
19	225.1	0.76	0.998	0.77	0.999	0.999	0.999
20	228.3	0.00	0.052	0.00	0.743	−0.007	0.104
21	230	0.01	0.880	0.01	0.977	−0.021	0.366
22	231.8	−0.42	0.994	−0.39	0.998	−0.392	0.996
23	233.1	−0.03	0.783	0.00	0.743	0.001	0.172
24	247.9	0.02	0.975	−0.00	0.997	0.004	0.970
25	251	−0.01	0.971	0.00	0.976	0.008	0.970
26	260.8	−0.00	0.991	−0.00	0.972	−0.011	0.974
27	267.6	−0.00	0.991	0.00	0.997	−0.008	0.913
28	309	2.48	0.999	2.48	0.999	2.521	0.999
29	315.2	−0.01	0.445	−0.01	0.586	−0.023	0.631
30	316.6	−0.05	0.942	−0.01	0.911	−0.009	0.945
31	339.8	−0.03	0.922	−0.00	0.736	−0.009	0.948
32	350.1	−0.04	0.902	−0.01	0.976	0.022	0.973
33	368.3	−3.76	0.999	−3.40	0.999	-3.334	0.999
34	380.2	−0.03	0.511	−0.02	0.310	−0.002	0.921
35	385	2.96	0.999	2.98	0.999	2.833	0.999
36	409.2	−0.02	0.969	−0.00	0.855	−0.007	0.985
37	465.7	0.01	0.976	0.00	0.917	−0.008	0.948
38	482.2	3.46	0.999	3.22	1.000	2.100	0.999
39	494.4	−0.01	0.988	0.00	0.997	0.009	0.996

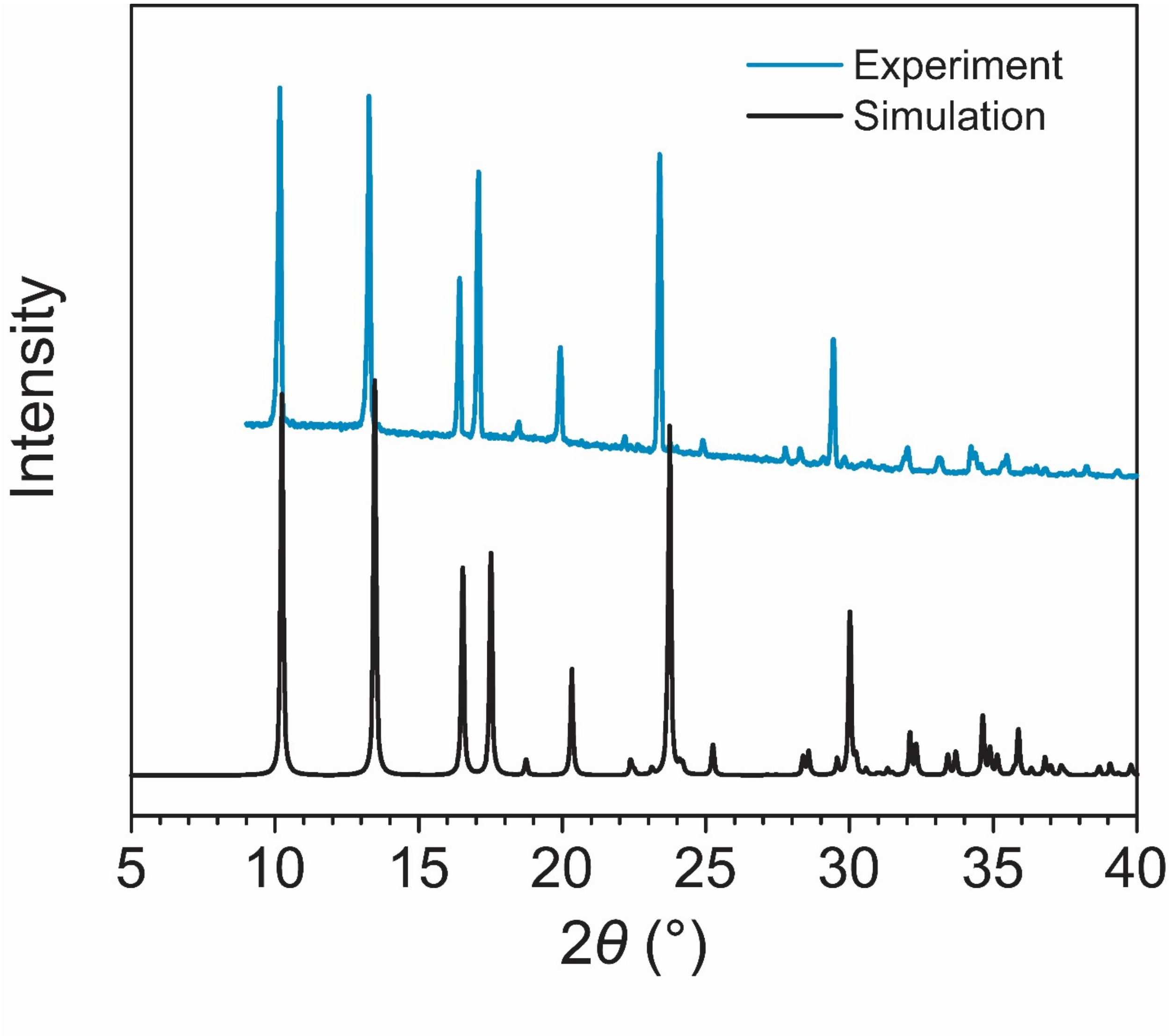

Figure S5 | PXRD comparison between ground crystals of **1** and the calculated pattern from our obtained crystal structure. The experimental pattern was collected at 298 K, and the simulation is calculated from the single crystal data taken at 100 K. Powder diffraction data were collected with pure CuKα (1.54065 Å) radiation.

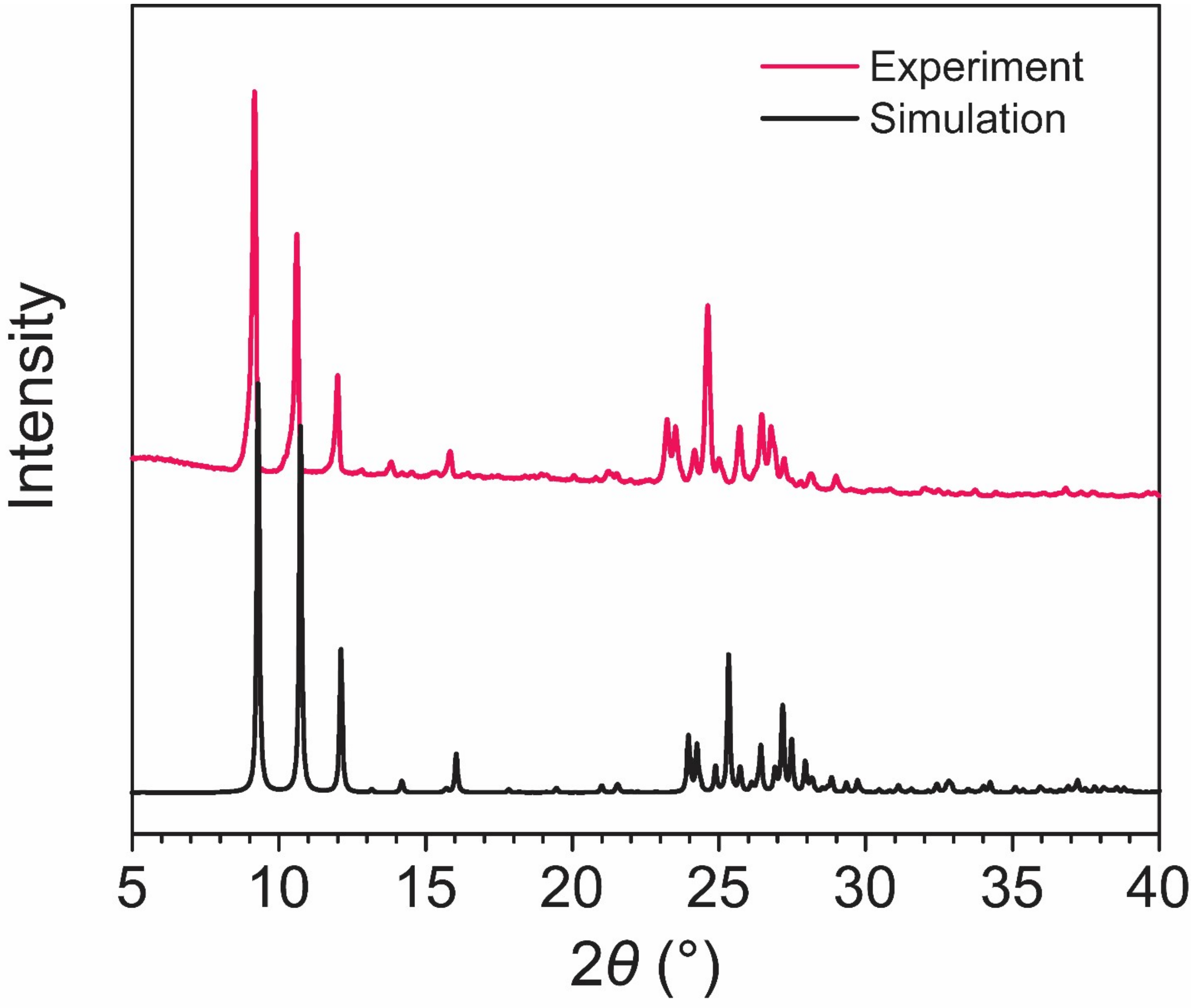

Figure S6 | PXRD comparison between ground crystals of **2** and the calculated pattern from our obtained crystal structure. The experimental pattern was collected at 298 K, and the simulation is calculated from the single crystal data taken at 100 K. Powder diffraction data were collected with pure CuKα (1.54065 Å) radiation.

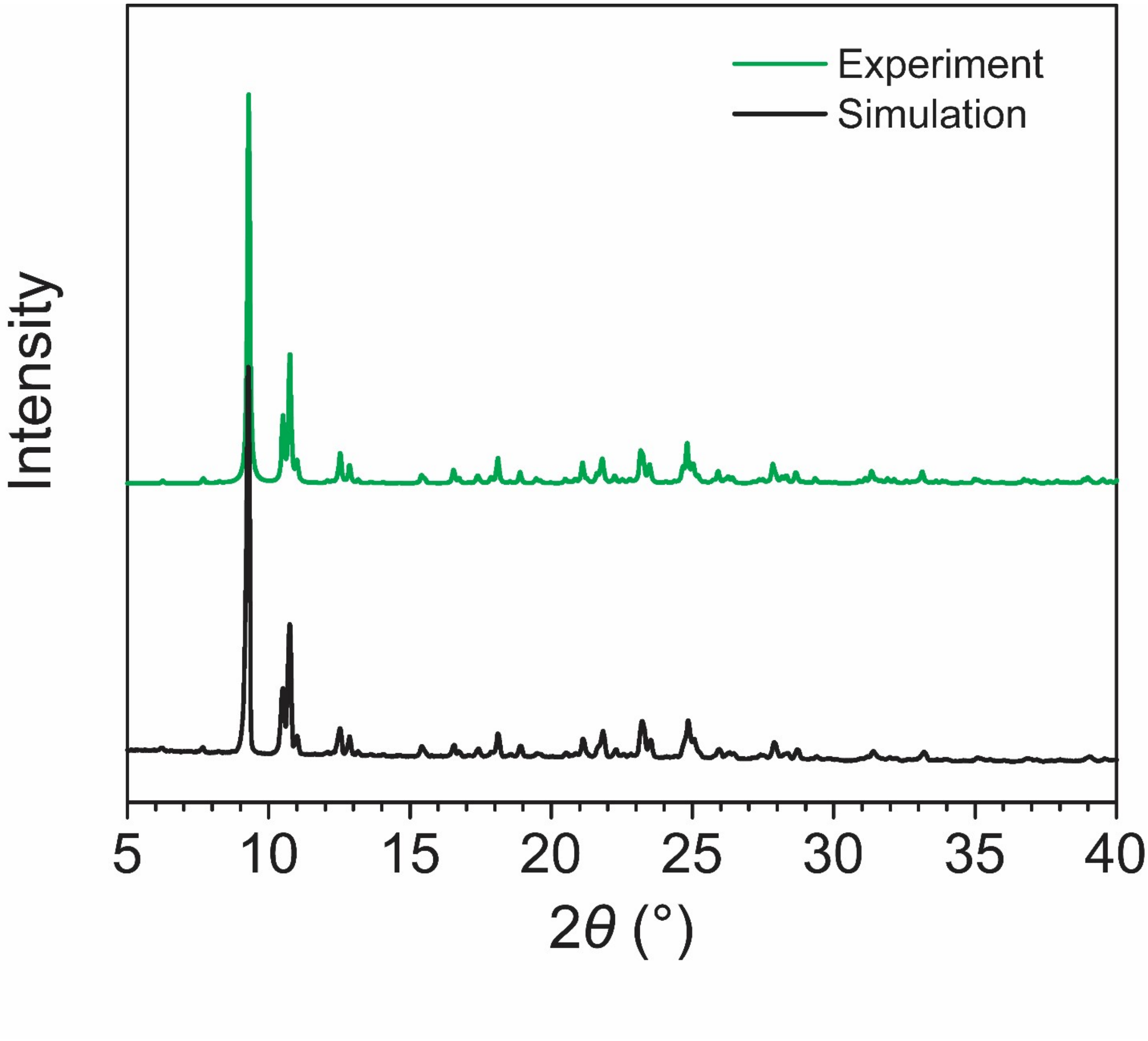

Figure S7 | PXRD comparison between ground up crystals of **3** with the literature reported structure.[21] Both data sets are at 298 K. Powder diffraction were was collected with pure CuKα (1.54065 Å) radiation.

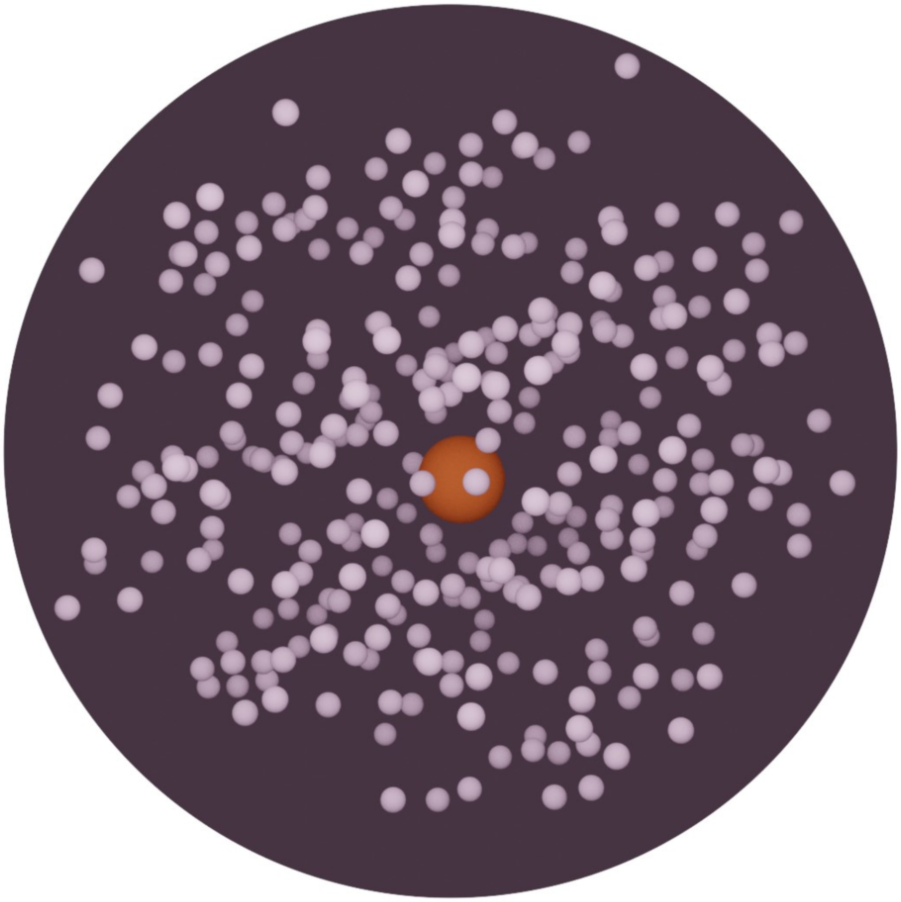

Figure S8 | A simplified view of the local environment of Cu(Me_2Nac)$_2$ (**1′**) in the solid state. This model shows all hydrogen atoms (white) within a 10 Å distance (represented by the purple sphere) from a copper metal center (orange). We attribute the unexpectedly short T_m for **1′** to this large proton density of more than 100 hydrogen atoms, many on free-rotating methyl groups.

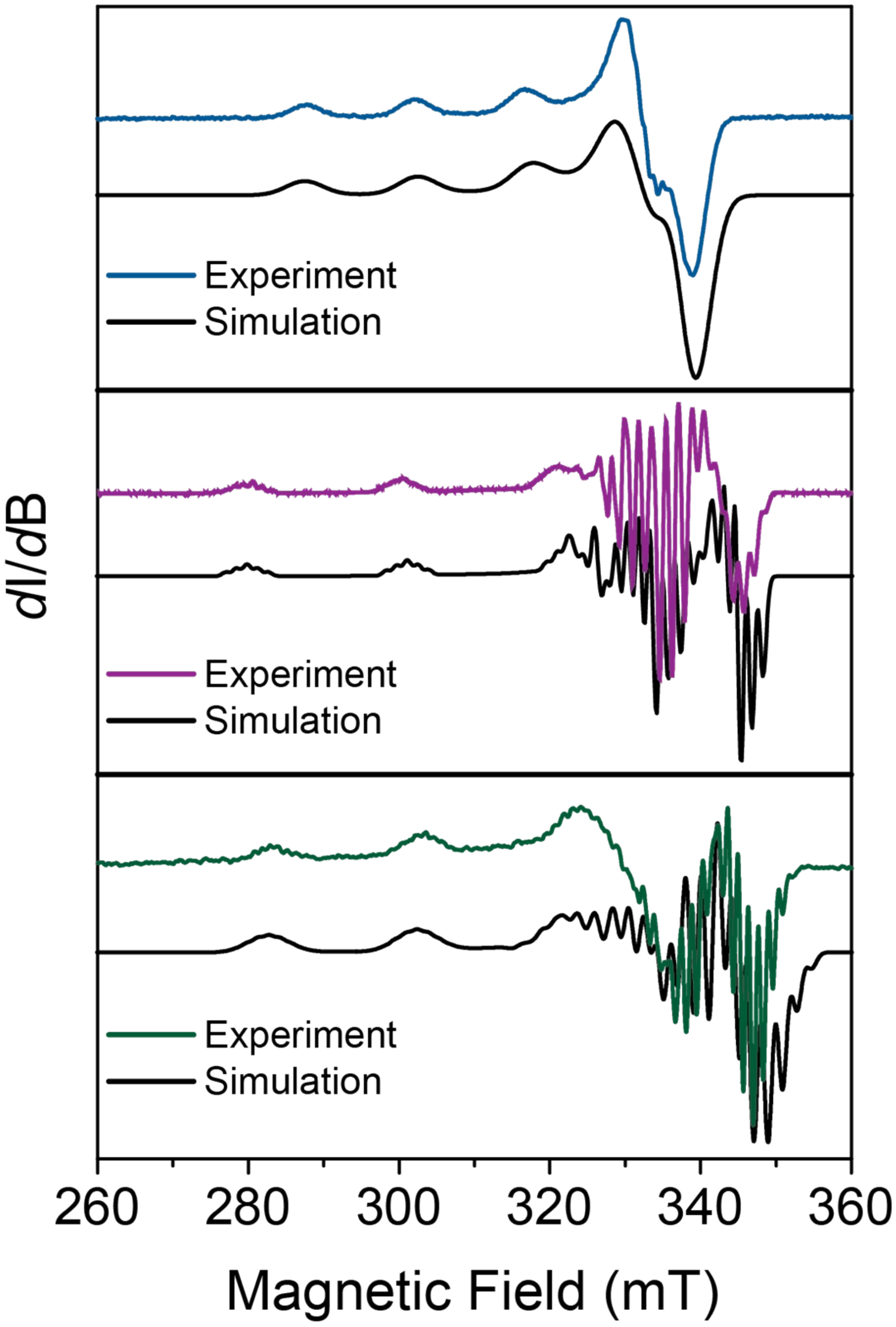

Figure S9 | CW spectra of **1** (top), **2** (middle), and **3** (bottom) taken in 0.1%$_w$ OTP at 60 K at X-band (9.540 GHz).

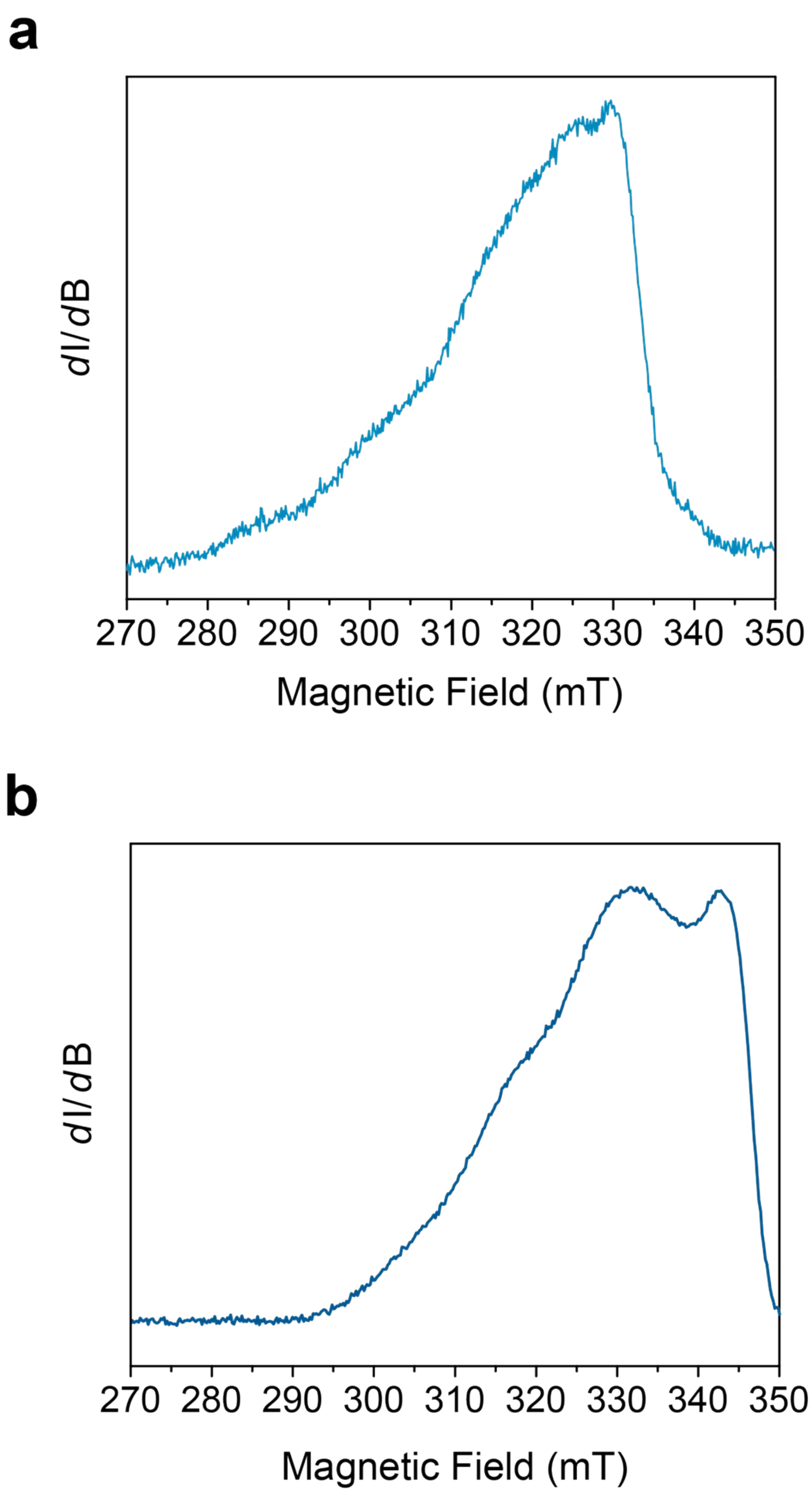

Figure S10 | Echo-detected field swept spectra for (**a**) **1′** (9.790 GHz, 5 K) and (**b**) **1′′** (9.384 GHz, 5 K)

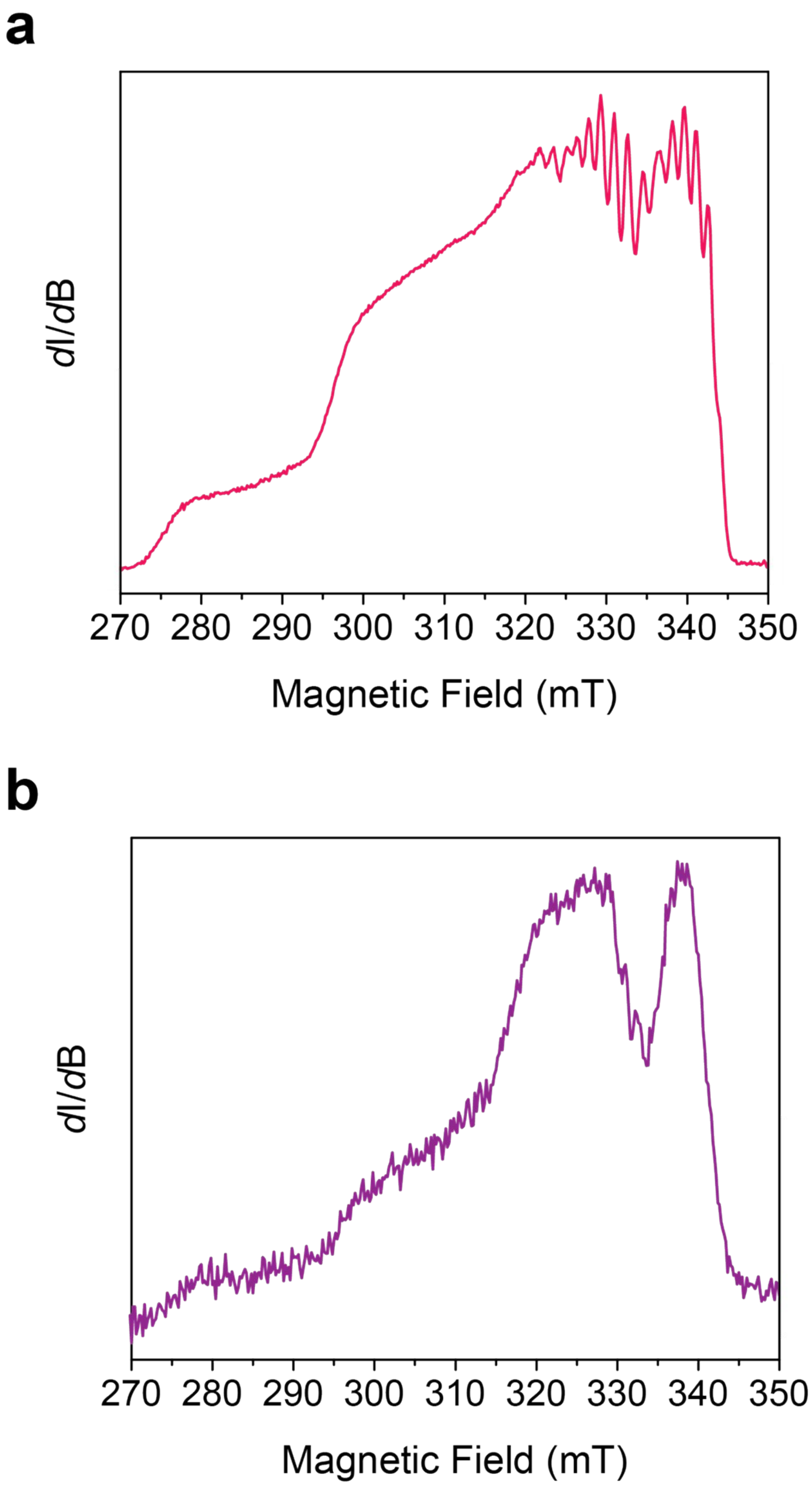

Figure S11 | Echo-detected field swept spectra for (**a**) **2′** (9.372 GHz, 5 K) and (**b**) **2″** (9.387 GHz, 5 K).

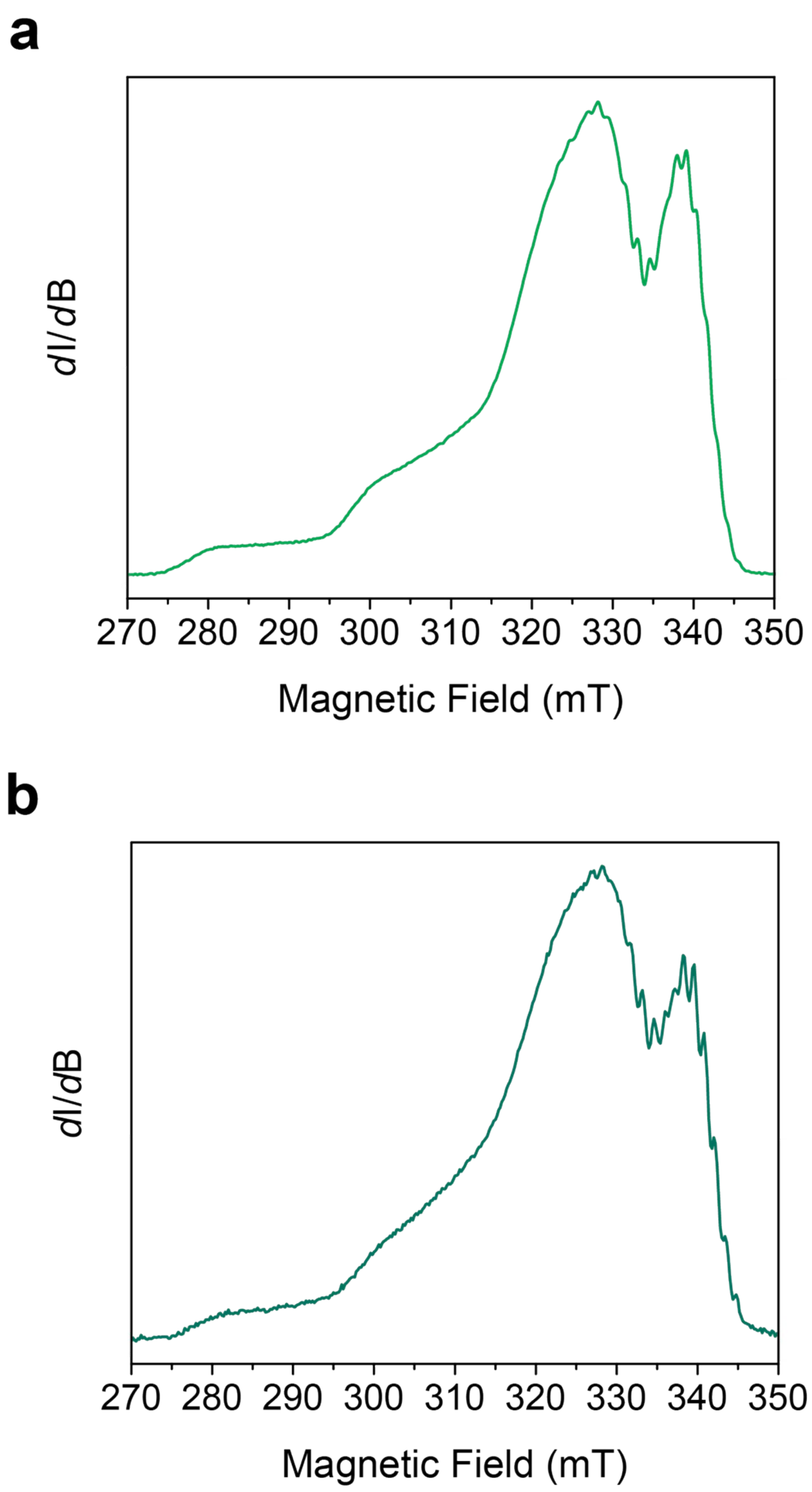

Figure S12 | Echo-detected field swept spectra for (**a**) **3′** (9.384 GHz, 5 K) and (**b**) **3′′** (9.377 GHz, 5 K)

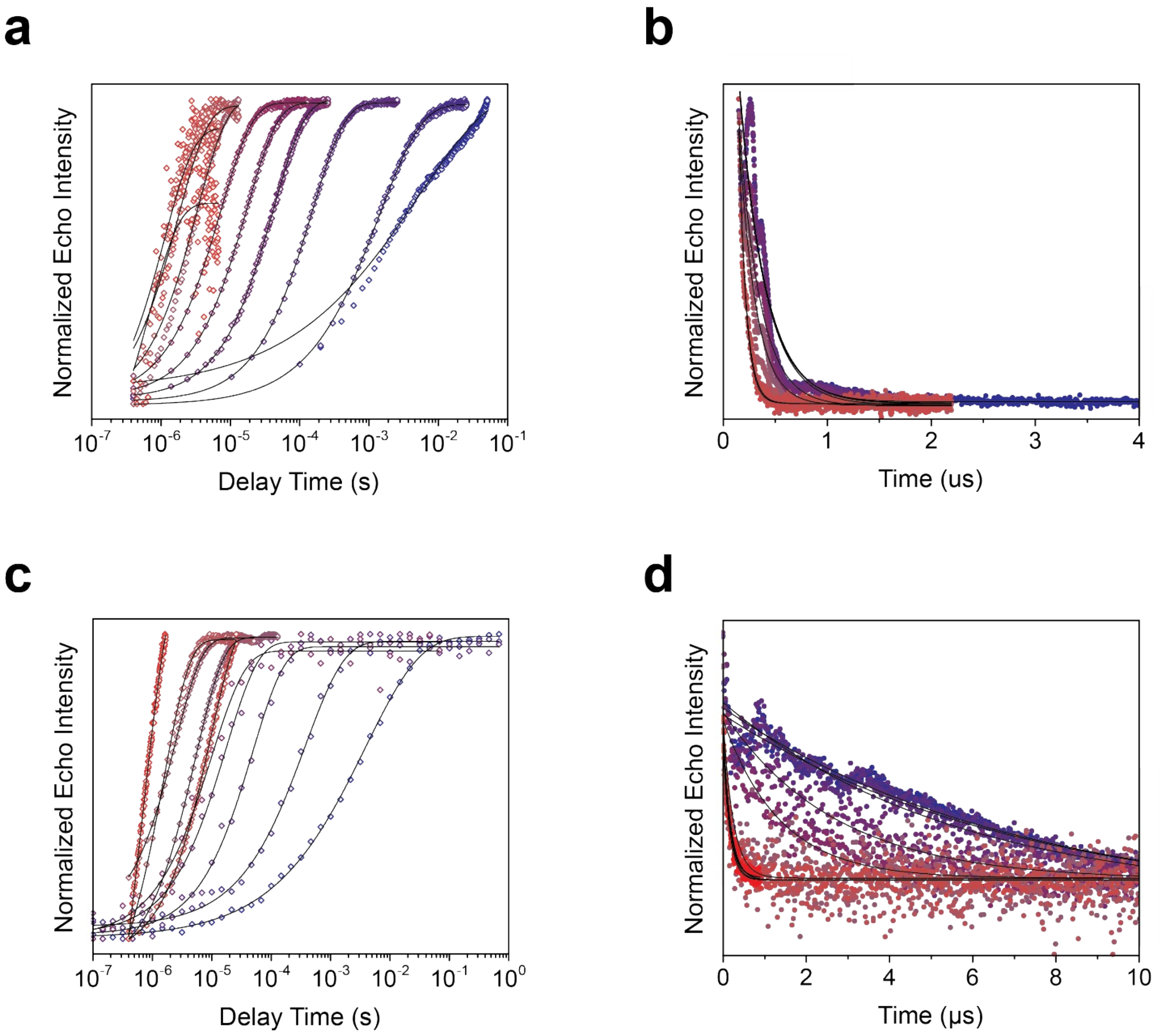

Figure S13 | Saturation recovery and Hahn echo decay curves for **1** progressing from the lowest temperature measured (blue, 5 K) to the highest (red, 90 K) at X-band (9.790 GHz, 332 mT for **1** and 9.384 GHz, 332 mT). (**a**) Saturation recovery **1′** (**b**) Hahn echo decay **1′** (**c**) Saturation recovery **1″** (**d**) Hahn echo decay **1″**. The T_1 of **1′** was collected with linear spacing, unlike the logarithmic spacing used for the other complexes. The larger error in fitting the tail has a minor effect on T_1, as the linear detection means that region of the curve is fit to only a small handful of data points – only the six shortest time points are poorly fit.

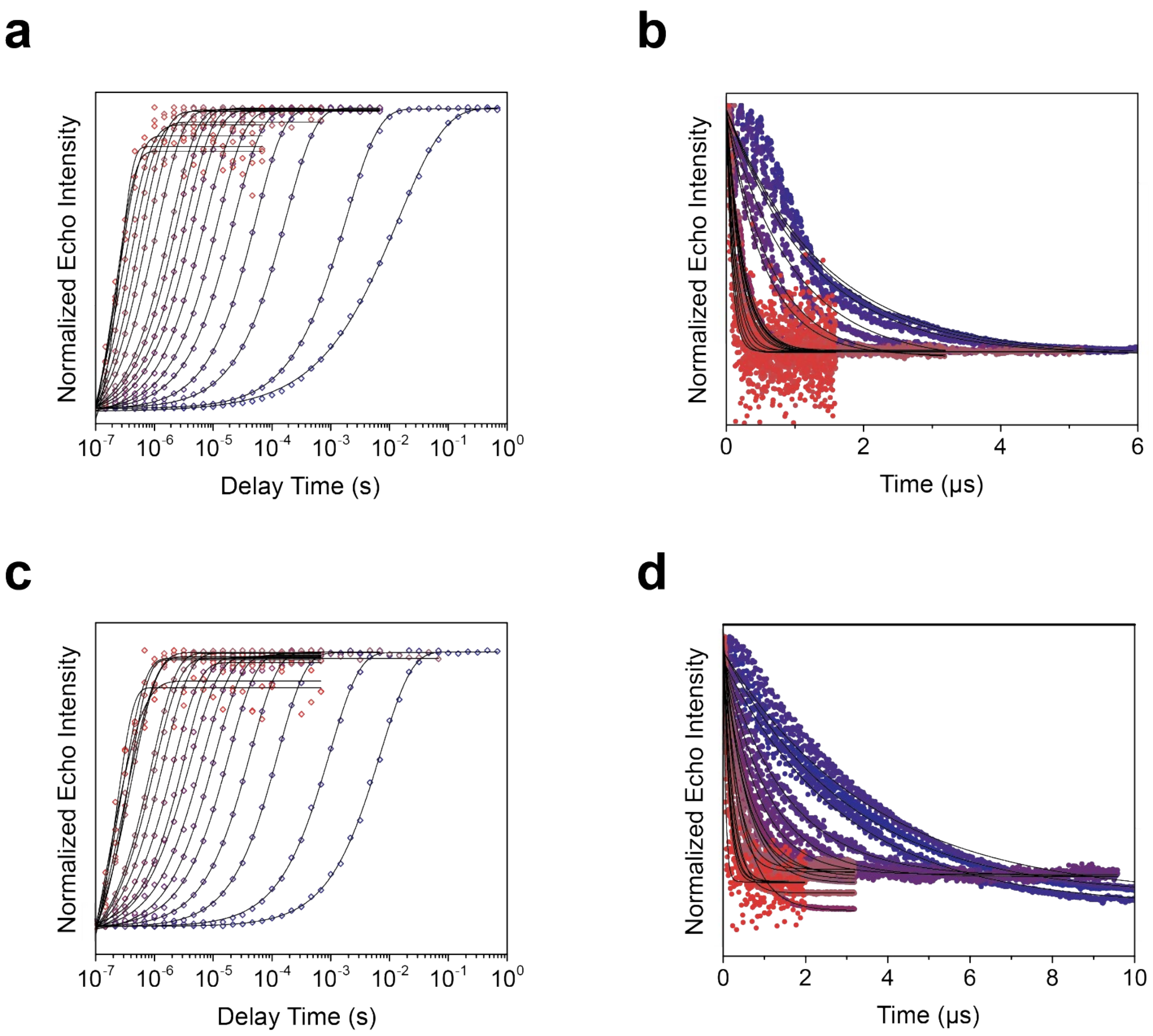

Figure S14 | Saturation recovery and Hahn echo decay curves for **2** progressing from the lowest temperature measured (blue, 5 K) to the highest (red, 260 K) at X-band (9.372 GHz, 329.2 mT for **2′** and 9.387 GHz, 337.0 mT for **2″**). (**a**) Saturation recovery **2′** (**b**) Hahn echo decay **2′** (**c**) Saturation recovery **2″** (**d**) Hahn echo decay **2″**.

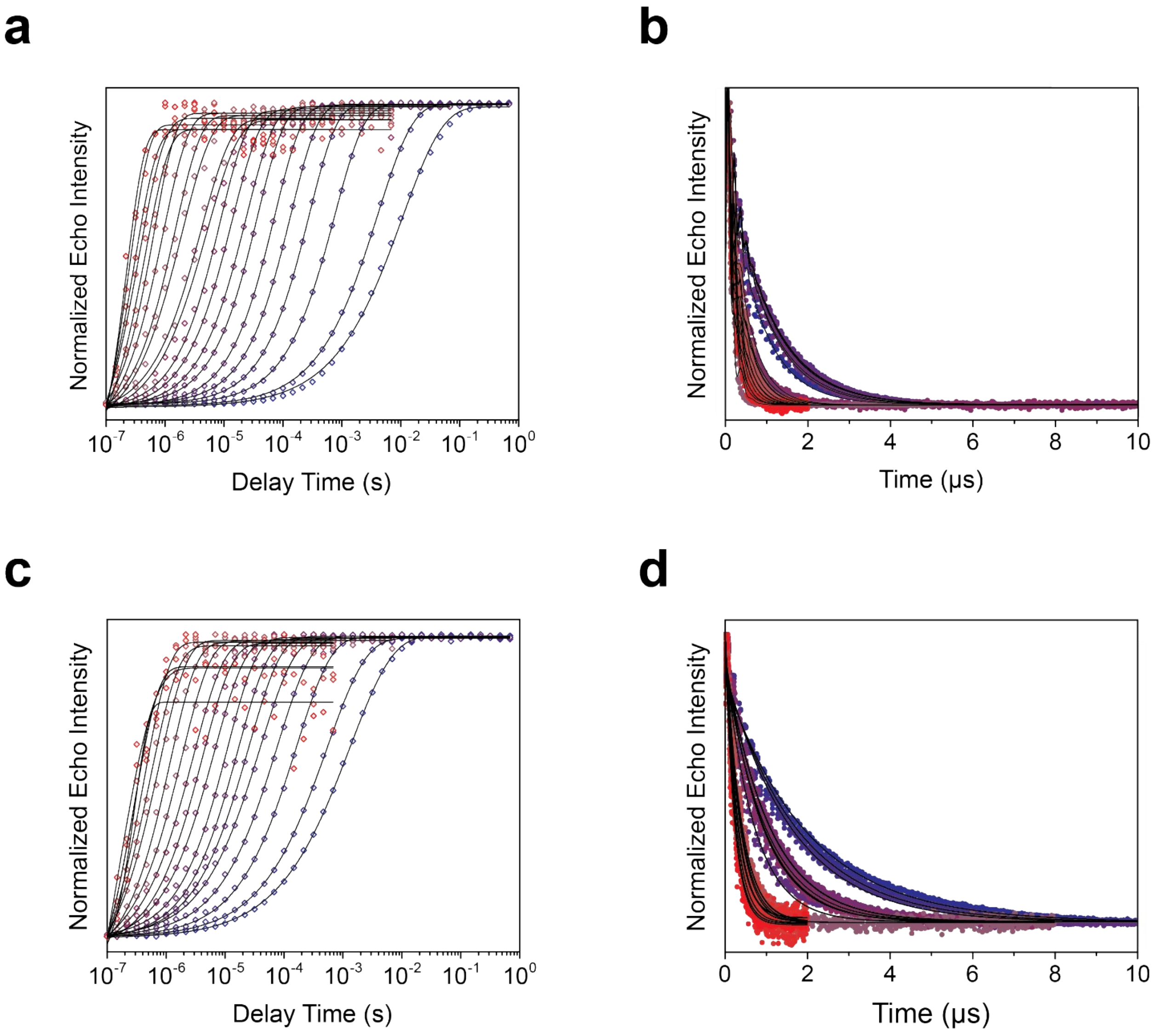

Figure S15 | Saturation recovery and Hahn echo decay curves for **3** progressing from the lowest temperature measured (blue, 5 K) to the highest (red, 300 K) at X-band (9.384 GHz, 328 mT for **3′** and 9.377 GHz and 328 mT for **3″**). (**a**) Saturation recovery **3′** (**b**) Hahn echo decay **3′** (**c**) Saturation recovery **3″** (**d**) Hahn echo decay **3″**.

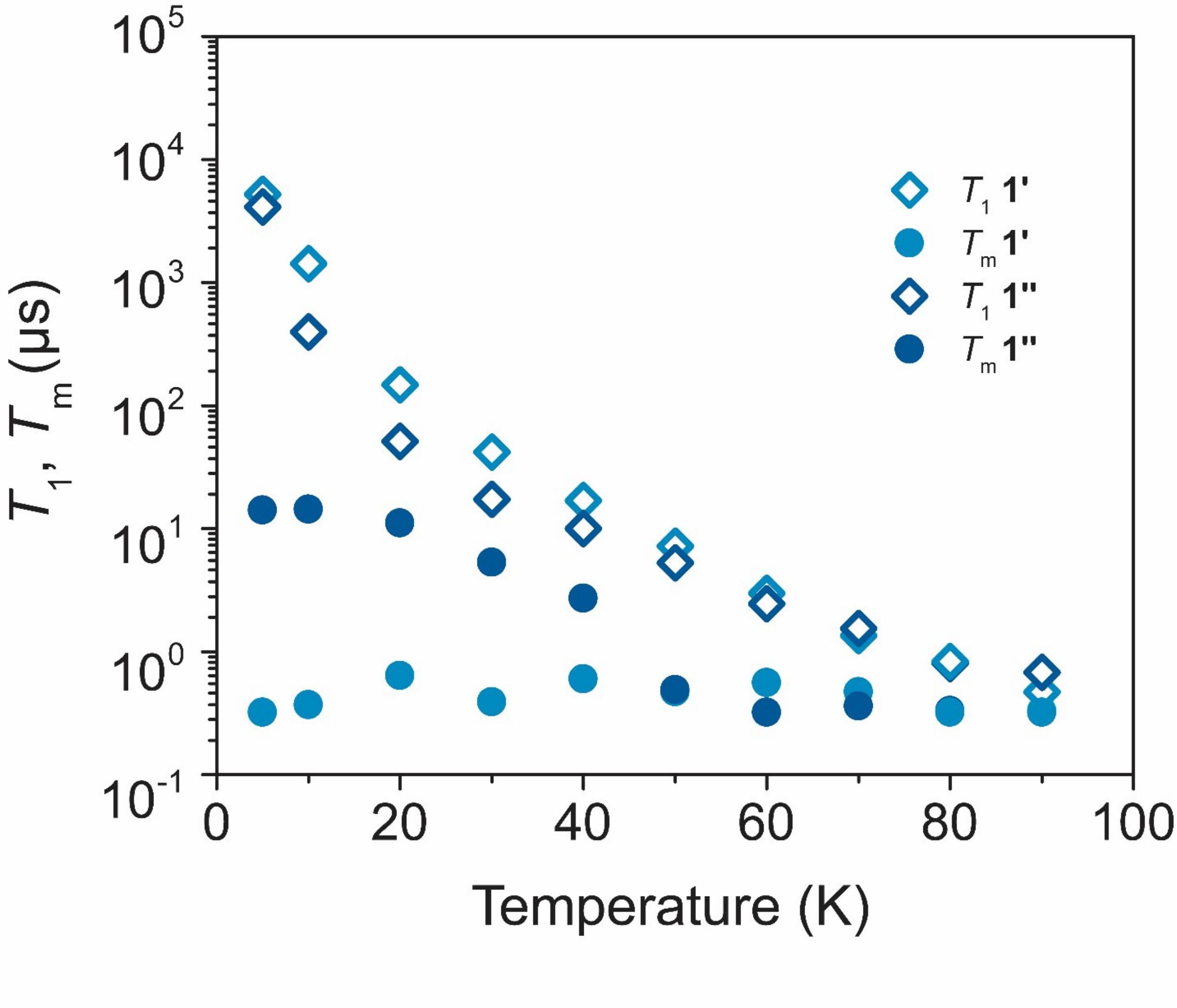

Figure S16 | T_1 and T_m vs temperature for **1'** and **1''** at X-band (9.790 GHz, 332 mT for **1** and 9.384 GHz, 332 mT).

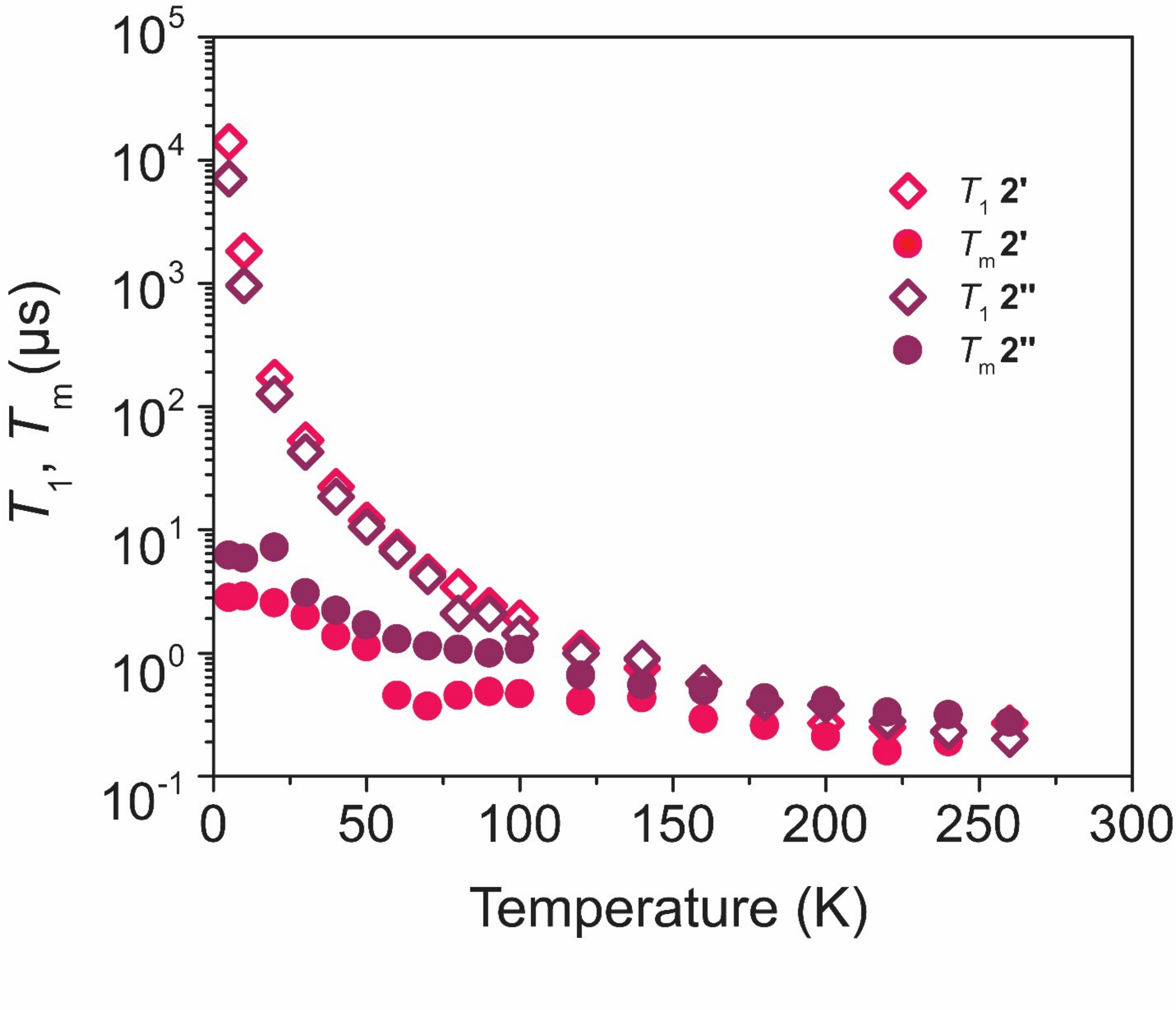

Figure S17 | T_1 and T_m vs temperature for **2'** and **2''** at X-band (9.372 GHz, 329.2 mT for **2′** and 9.387 GHz, 337.0 mT for **2′′**).

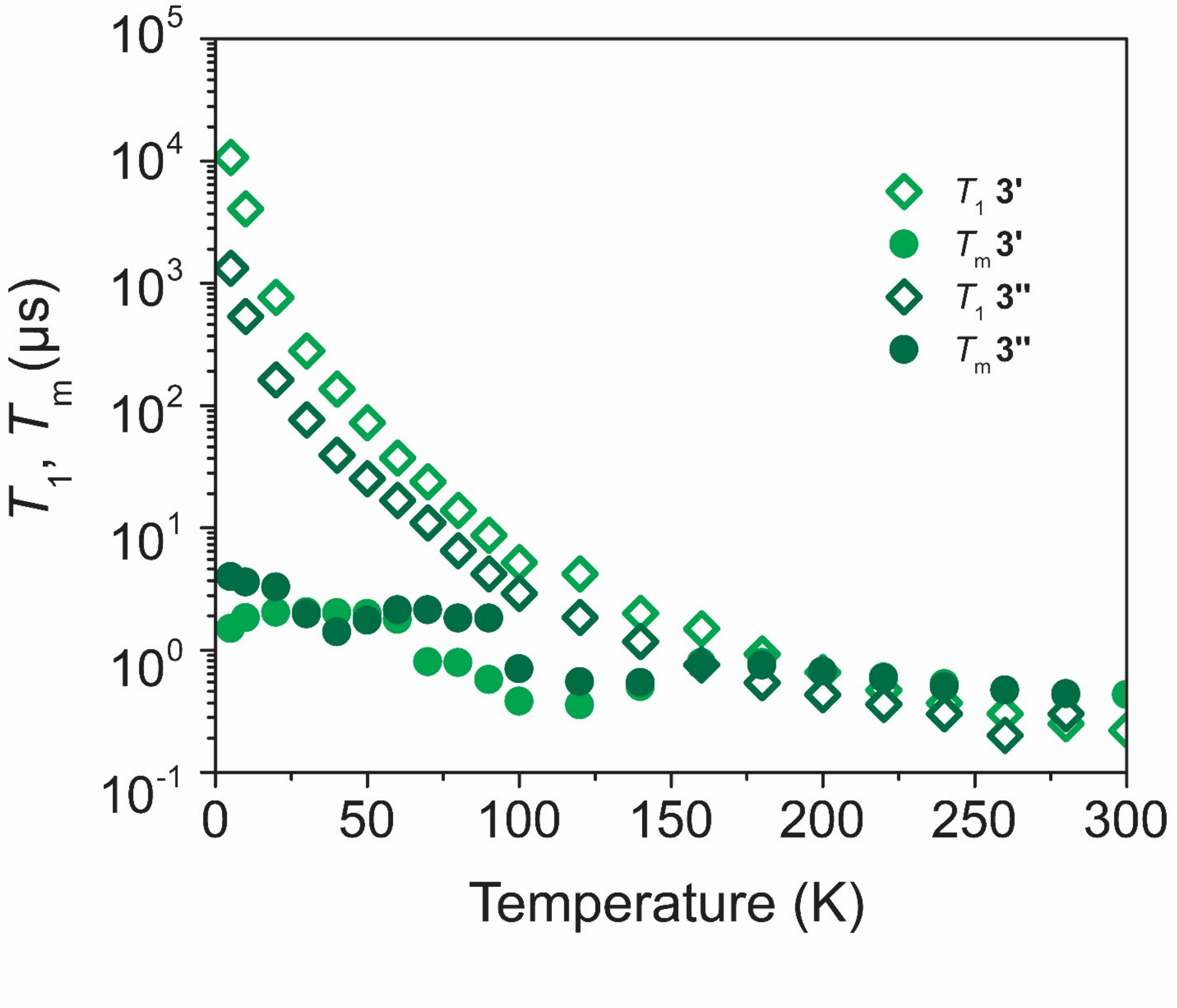

Figure S18 | T_1 and T_m vs temperature for **3'** and **3''** at X-band (9.384 GHz, 328 mT for **3′** and 9.377 GHz and 328 mT for **3′′**).

a

b

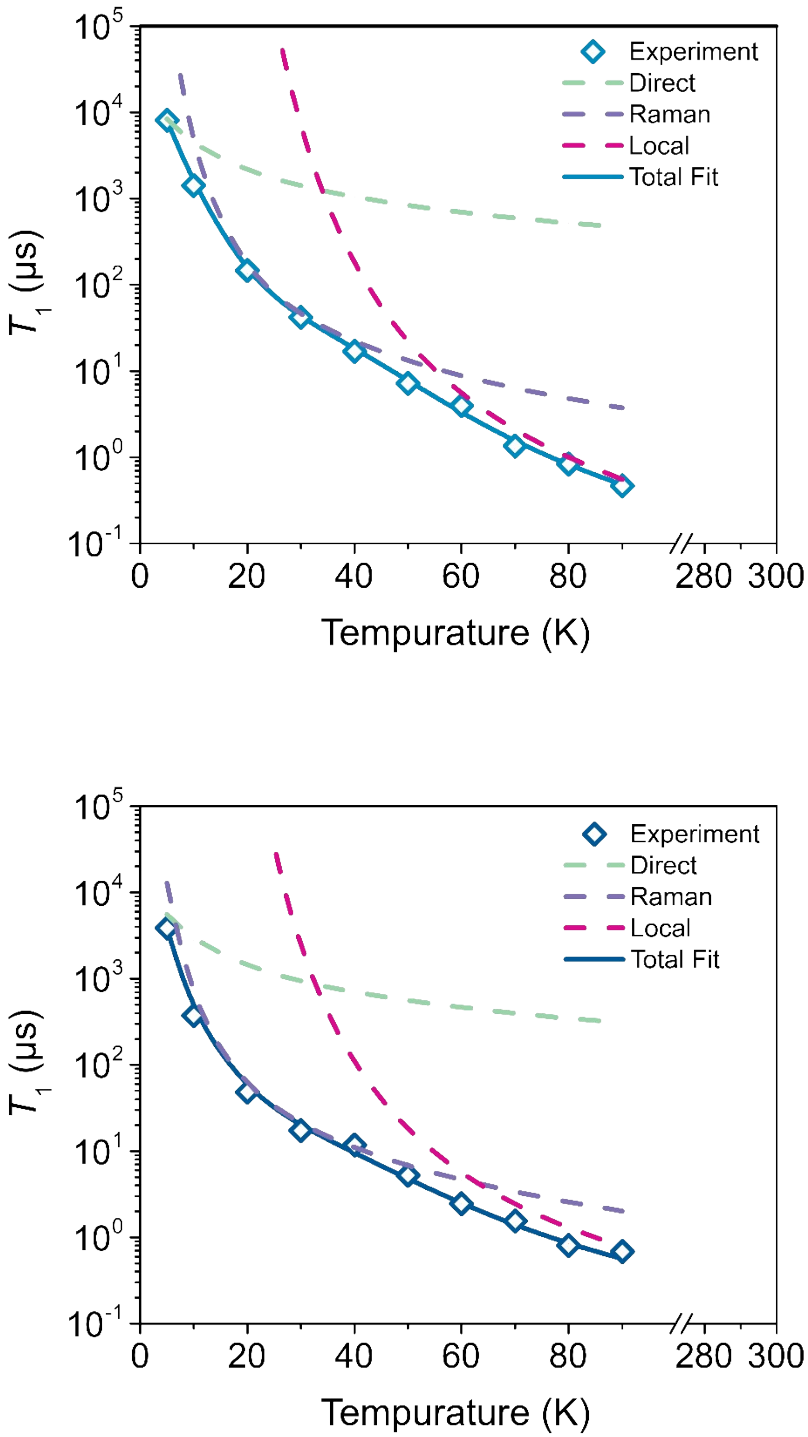

Figure S19 | Fits of the T_1 data for **1′** (**a**) and **1″** (**b**) using the Debye model. Data were taken at X-band (9.790 GHz, 332 mT for **1** and 9.384 GHz, 332 mT).

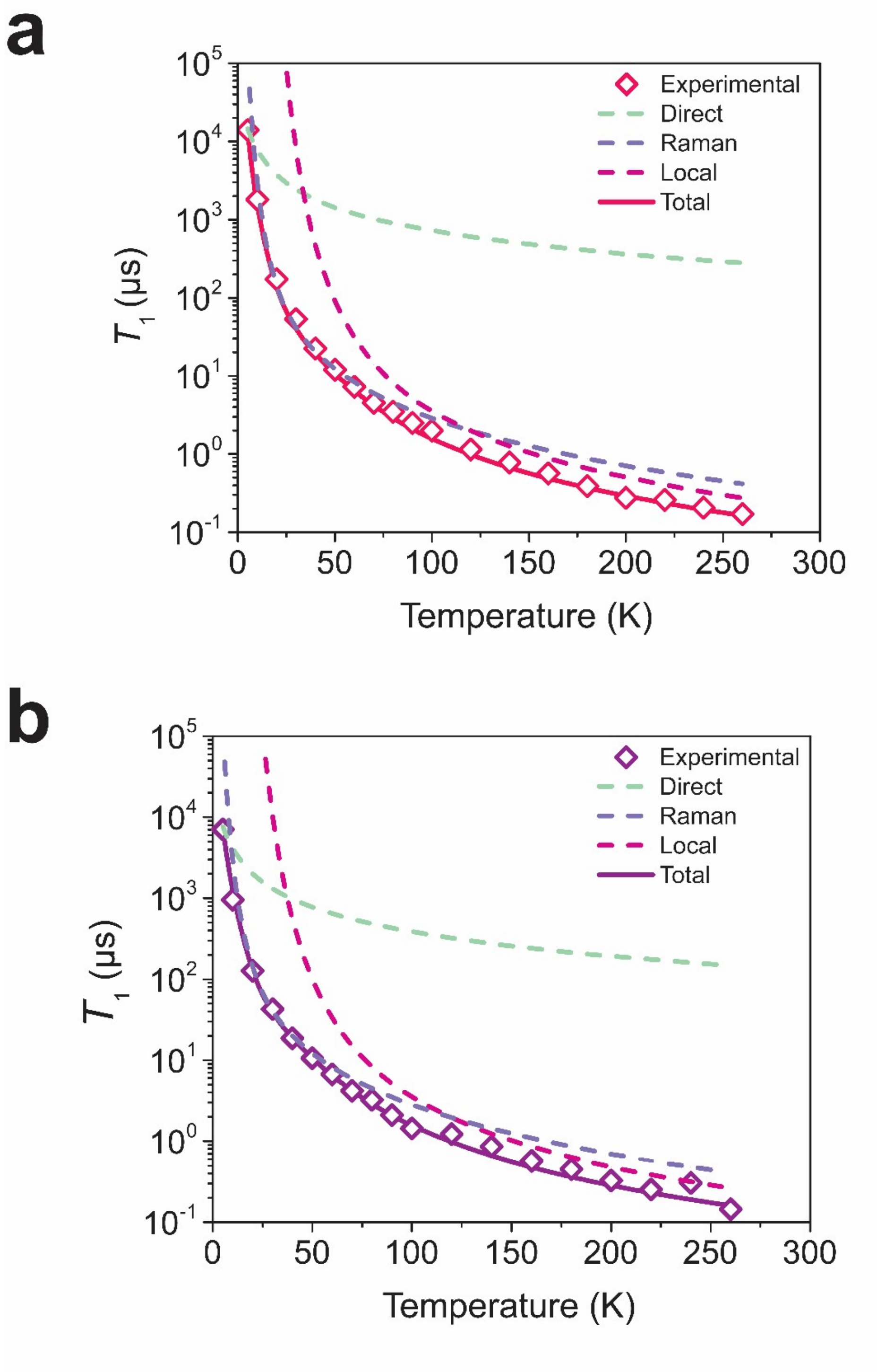

Figure S20 | Fits of the T_1 data for **2′** (**a**) and **2″** (**b**) using the Debye model. Data were taken at X-band (9.372 GHz, 329.2 mT for **2′** and 9.387 GHz, 337.0 mT for **2″**).

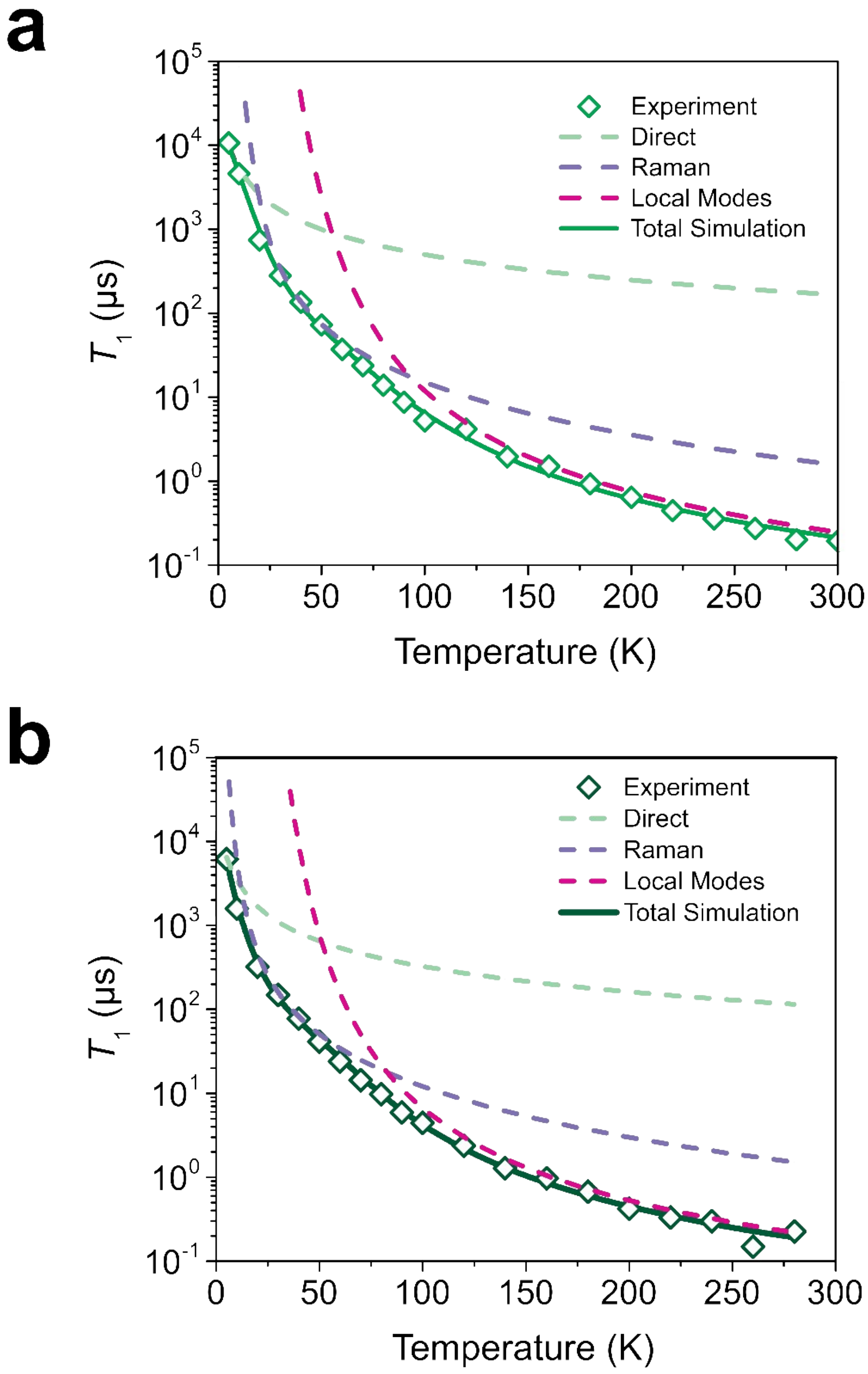

Figure S21 | Fits of the T_1 data for **3′** (**a**) and **3″** (**b**) using the Debye model. Data were acquired at X-band (9.384 GHz, 328 mT for **3′** and 9.377 GHz and 328 mT for **3″**).

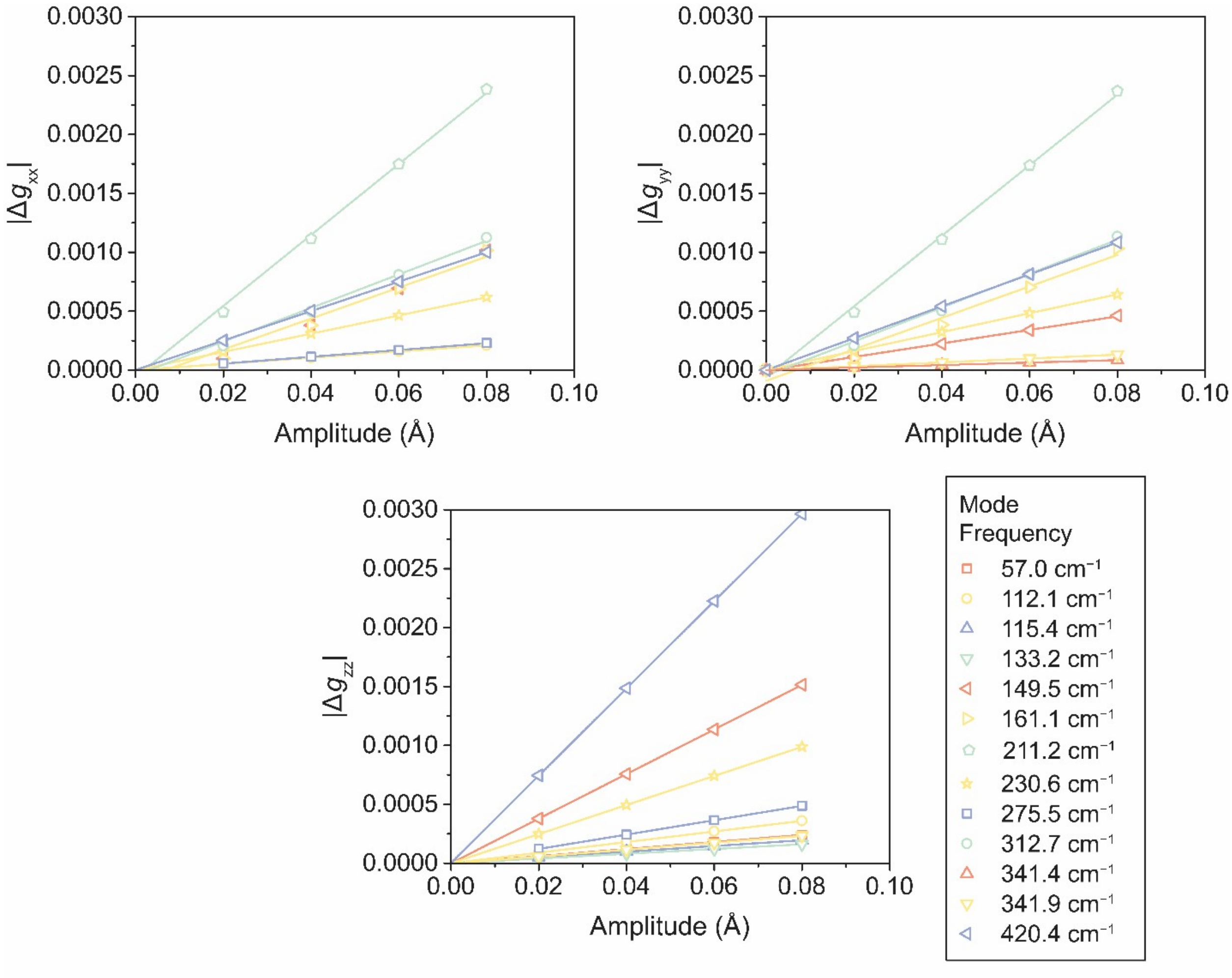

Figure S22 | The absolute value of the change in the x, y, and z components of the *g*-tensor from the ground state calculated for distorted structures corresponding to strongly coupled vibrational modes for **1**. Displacement vectors and *g*-tensor values are only shown for the eight most strongly coupled modes for each component of the *g* tensor.

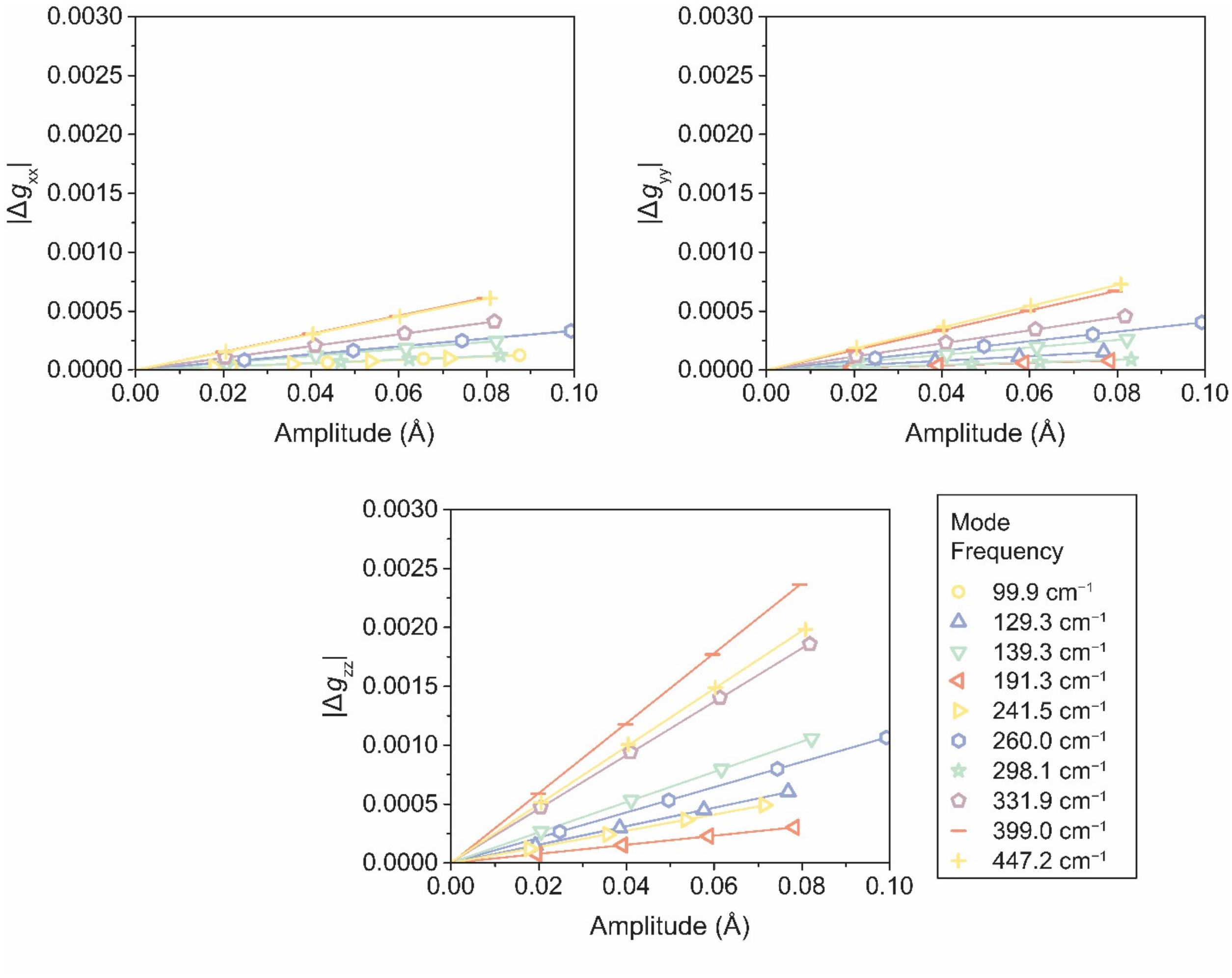

Figure S23 | The absolute value of the change in the x, y, and z components of the *g*-tensor from the ground state calculated for distorted structures corresponding to strongly coupled vibrational modes for **2.** Displacement vectors and *g*-tensor values are only shown for the eight most strongly coupled modes for each component of the *g* tensor.

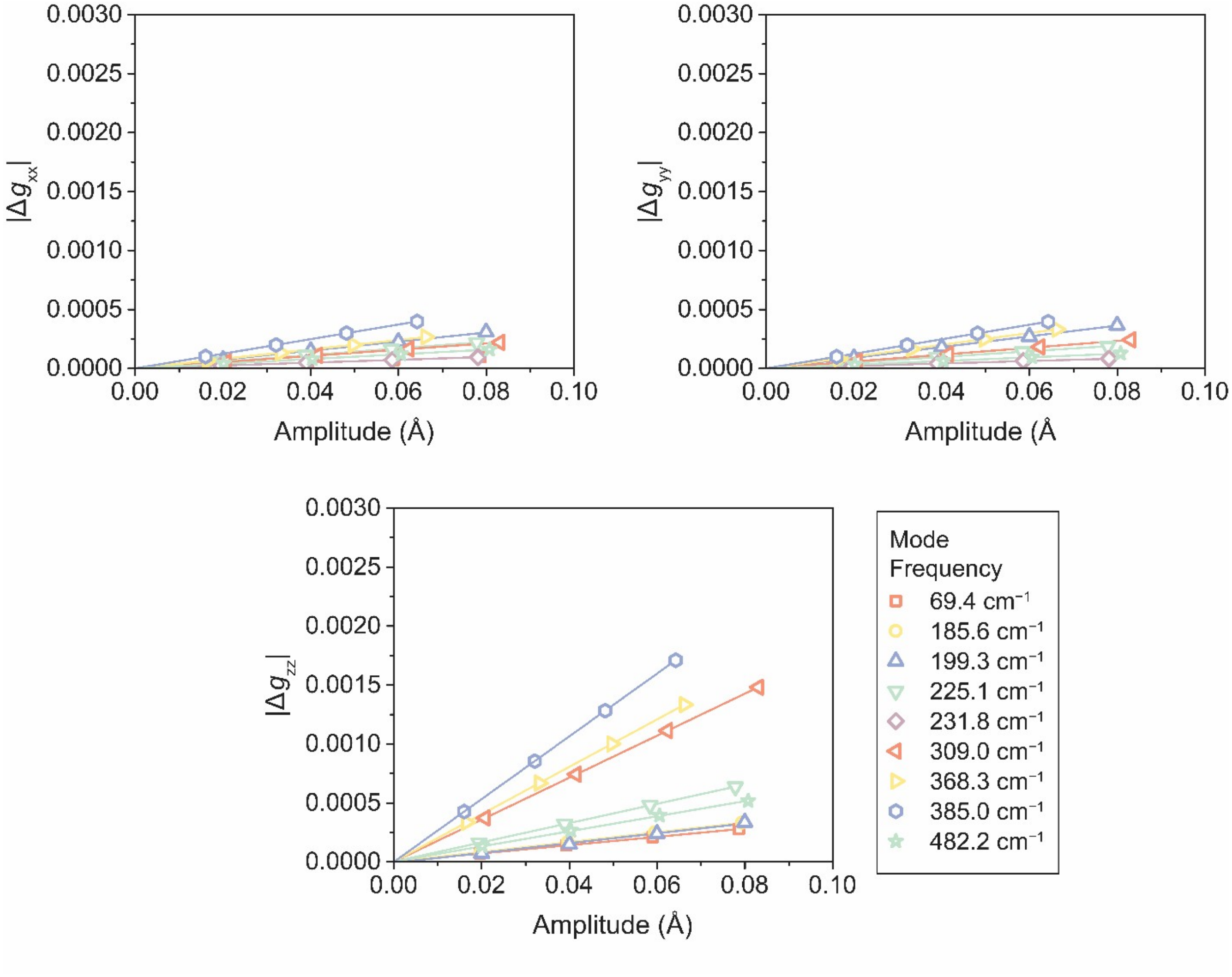

Figure S24 | The absolute value of the change in the x, y, and z components of the *g*-tensor from the ground state calculated for distorted structures corresponding to strongly coupled vibrational modes for **3**. Displacement vectors and *g*-tensor values are only shown for values are only shown for the eight most strongly coupled modes with significant changes in *g*-tensor ($\frac{\partial g}{\partial Q}$ > 0.001 Å^{-1}), except for along g_{yy}, where only seven modes had $\frac{\partial g}{\partial Q}$ > 0.001 Å^{-1}.

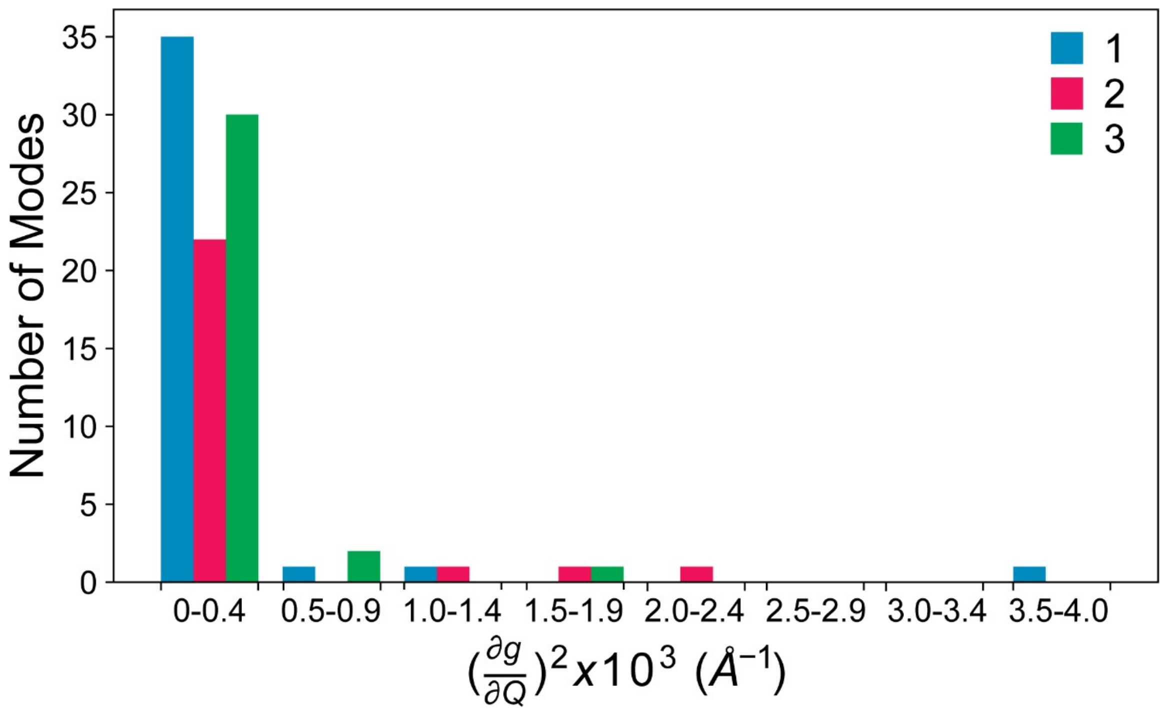

Figure S25 | Histograms showing the SPC of all local modes between 0 and 500 cm^{-1} in **1**, **2**, and **3**. The vast majority of local modes are weakly coupled ($\left(\frac{\partial g}{\partial Q}\right)^2$ < 0.0001 Å^{-1}). A small number of modes in each complex have significantly stronger coupling, and drive rapid relaxation in these complexes.

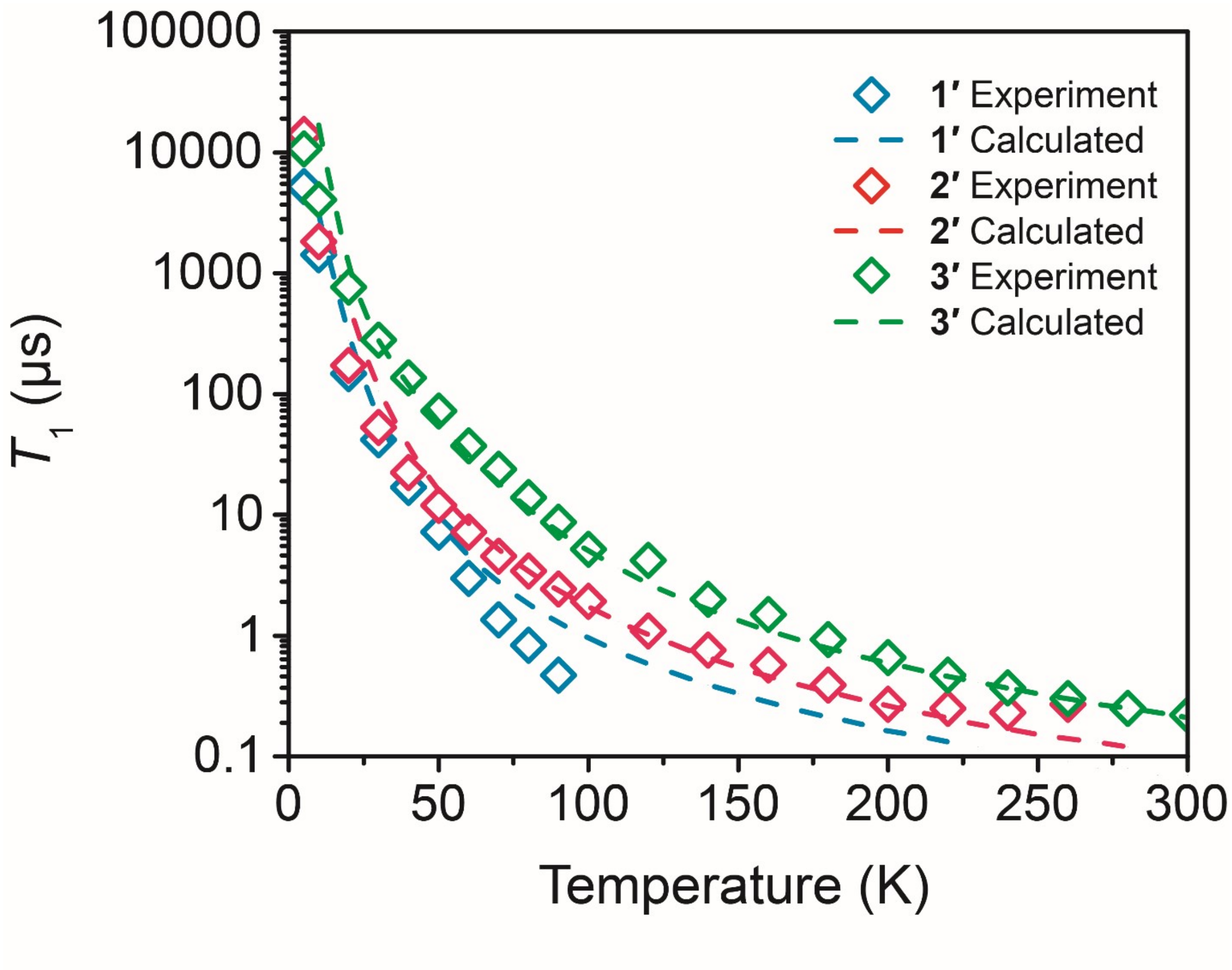

Figure S26 | Comparison of experimental T_1 values with calculated T_1 values from **Eq 6** for **1′–3′**. The calculated values match the experimental values well at high temperatures, but tend to deviate at lower temperatures where unmodeled lattice vibrations dominate.

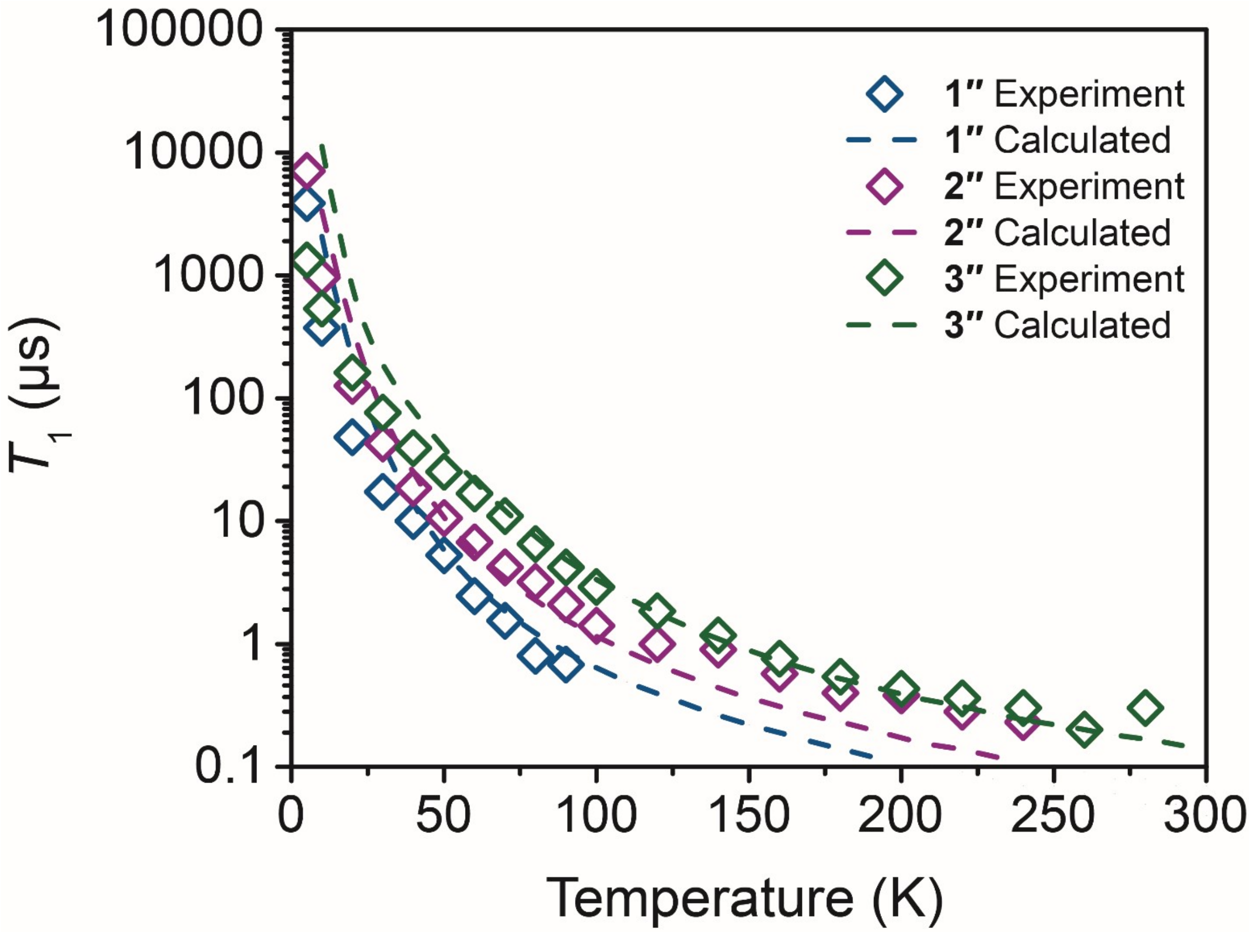

Figure S27 | Comparison of experimental T_1 values with calculated T_1 values from **Eq 6** for **1″–3″**. The calculated values match the experimental values well at high temperatures, but tend to deviate at lower temperatures where unmodeled lattice vibrations dominate. Additionally, these calculated values seem to deviate more from experiment than **1′–3′**, likely as a result of the complexity of phonon interactions in solution.

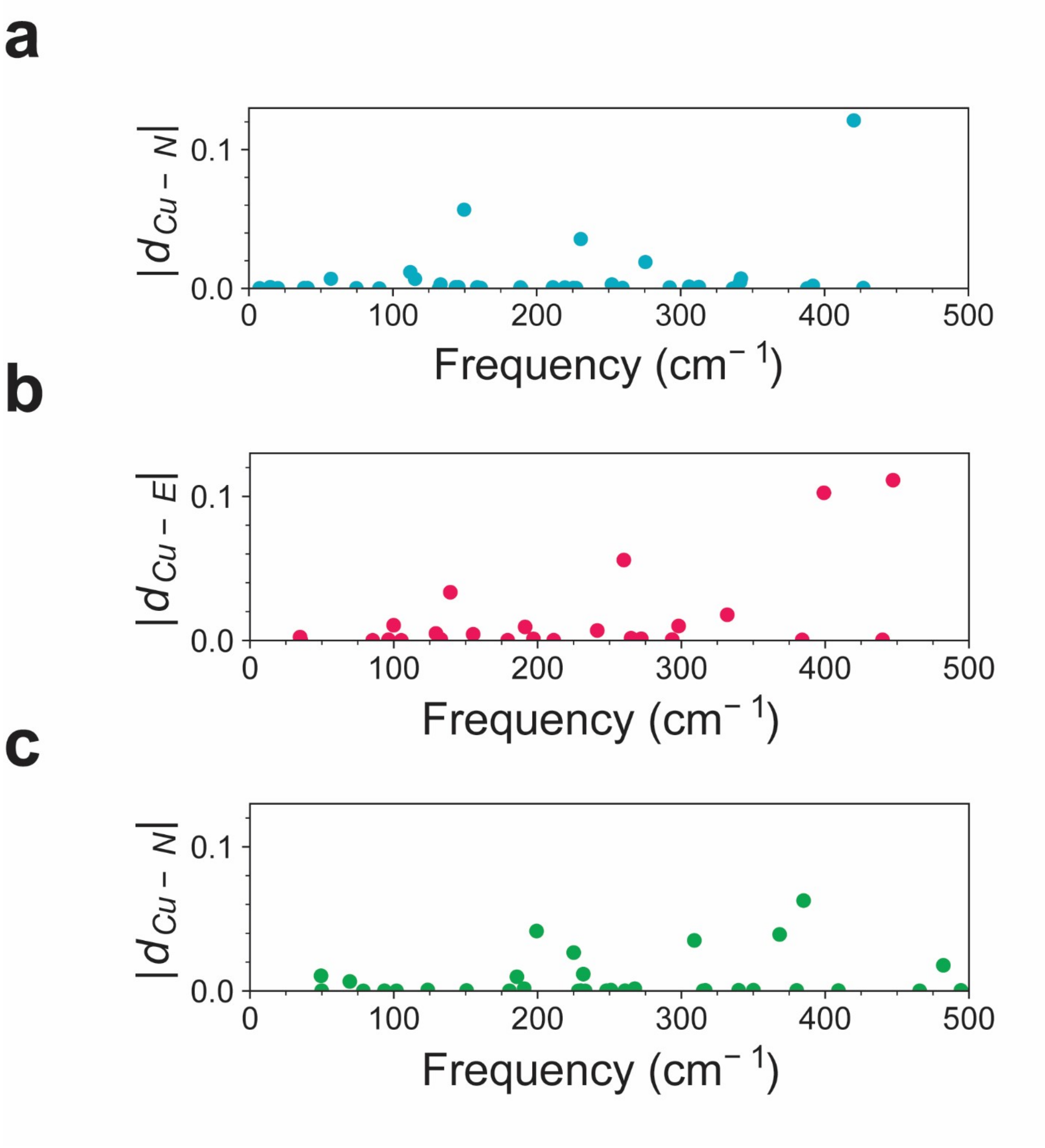

Figure S28 | Average absolute value of the change in the bond distance metric between the Cu metal center and each of the donor atoms for each of the modes. (**a**) **1** (**b**) **2** (**c**) **3**

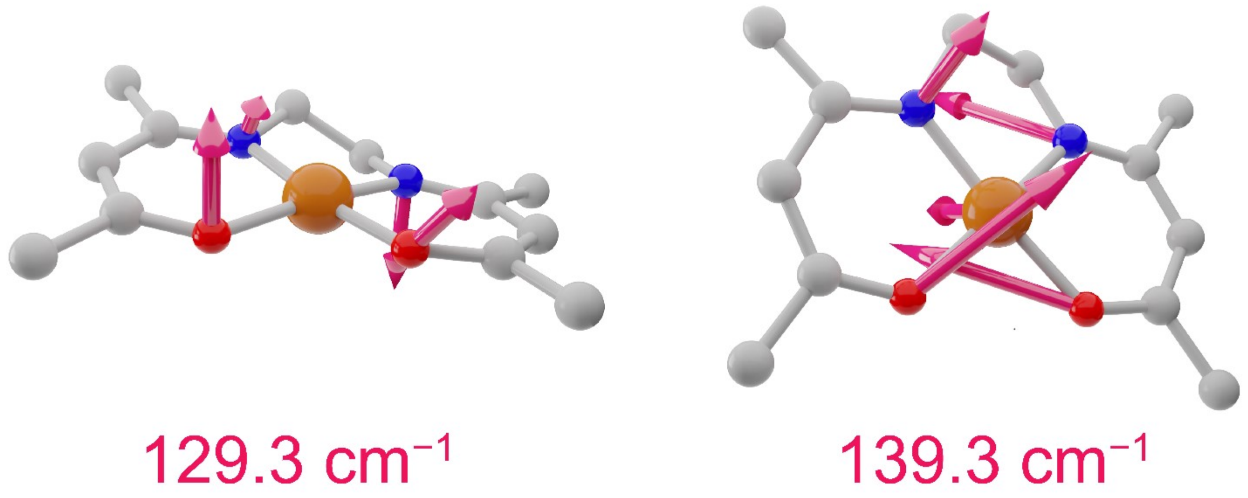

Figure S29 | Visualization of the motion of the primary coordination sphere in **2** along the strongly coupled low energy vibrational modes at 129.3 cm^{-1} (left) and 139.3 cm^{-1} (right). The untethered oxygen atoms have large displacements, causing large perturbations of the spin center and driving rapid relaxation.

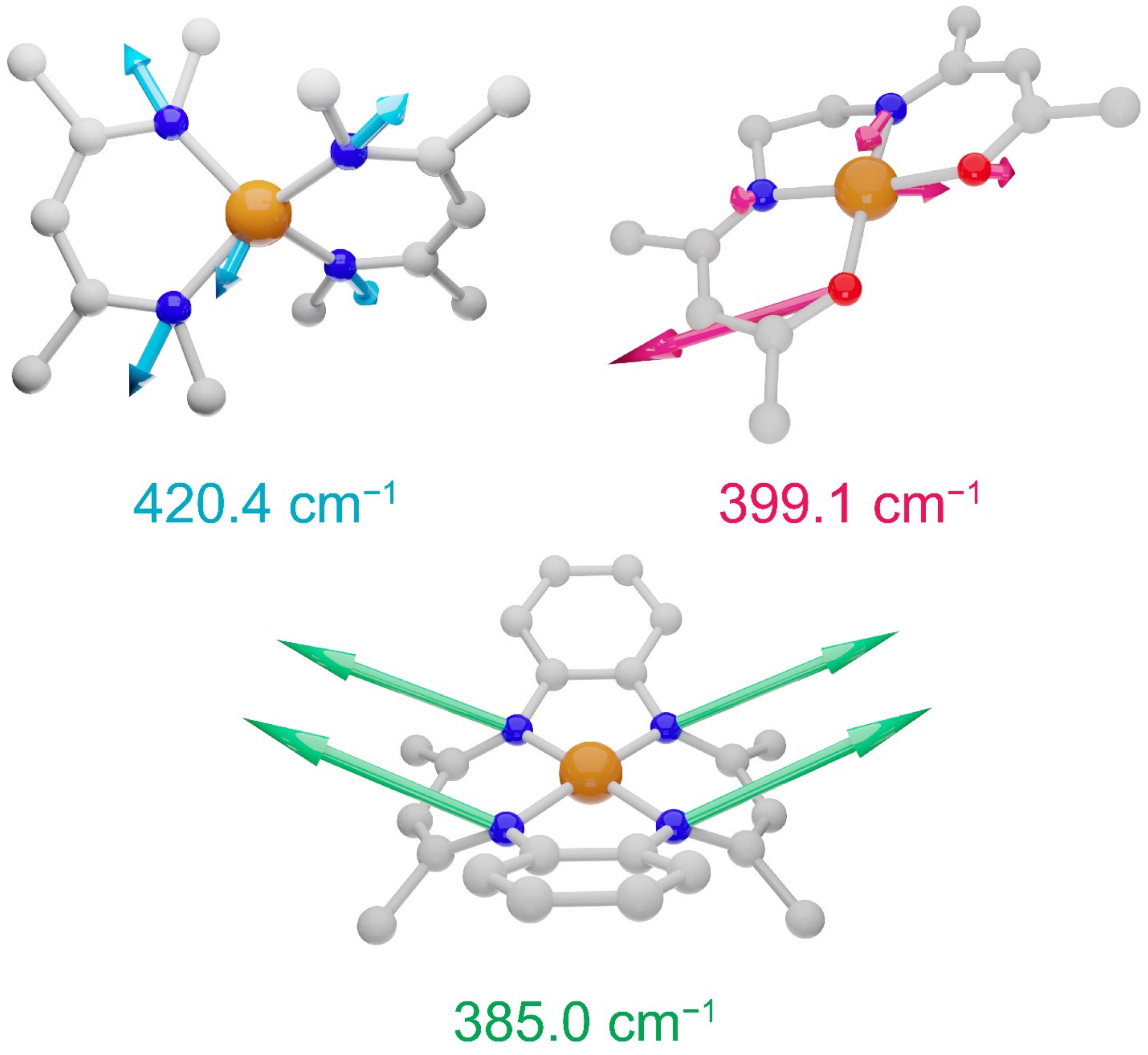

Figure S30 | The motion of the primary coordination sphere involved in the vibrational modes with the highest V_{sph}^2 coupling constant in each complex, along with the energy of the mode. Though **3** has the lowest energy of these modes, the slower net relaxation is a result of fewer strongly coupled low energy modes.

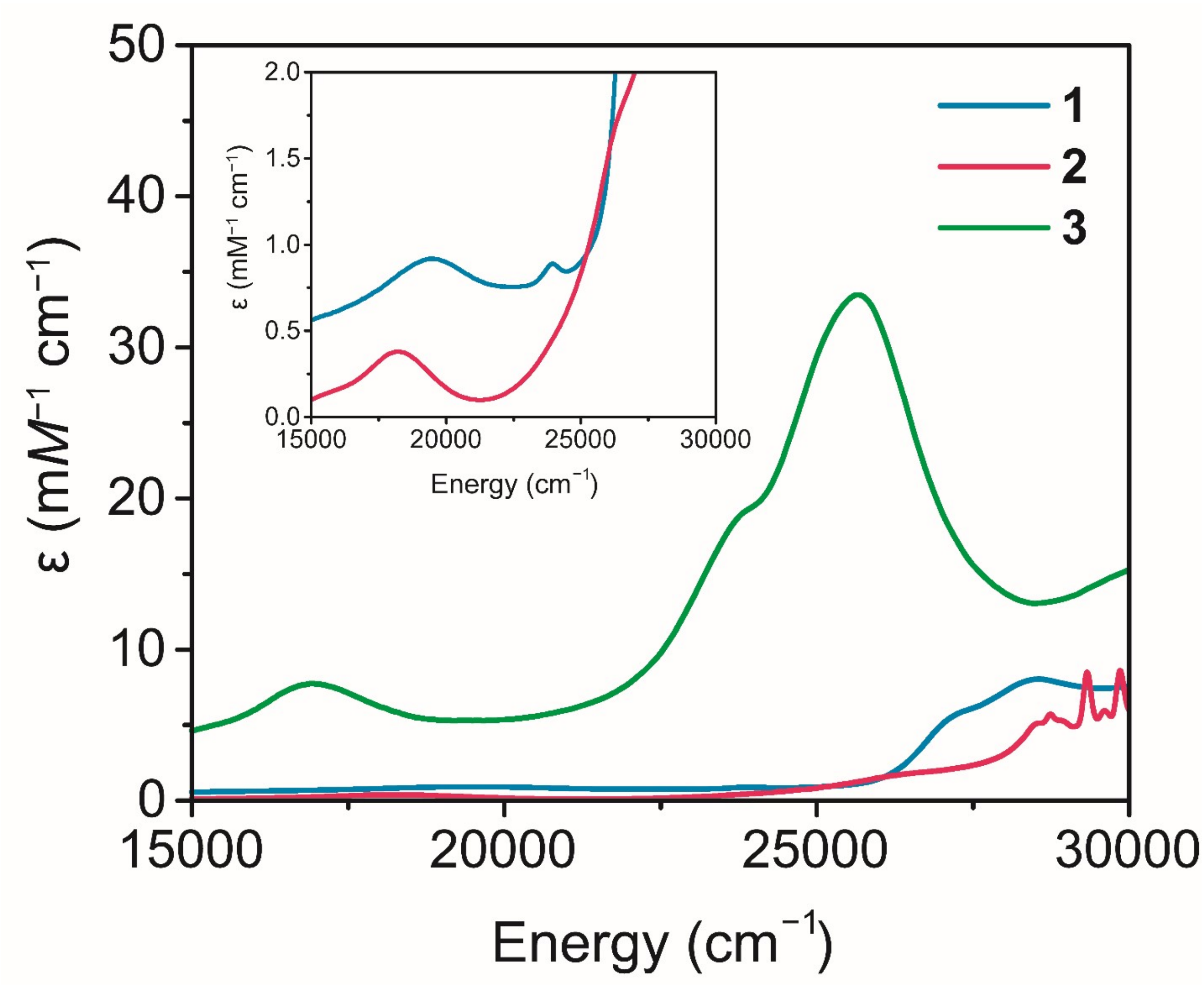

Figure S31 | UV-VIS spectra of **1**–**3** in toluene at room temperature. Inset: A better view of the transitions present in **1** and **2** with significantly lower molar absorptivity than **3**.

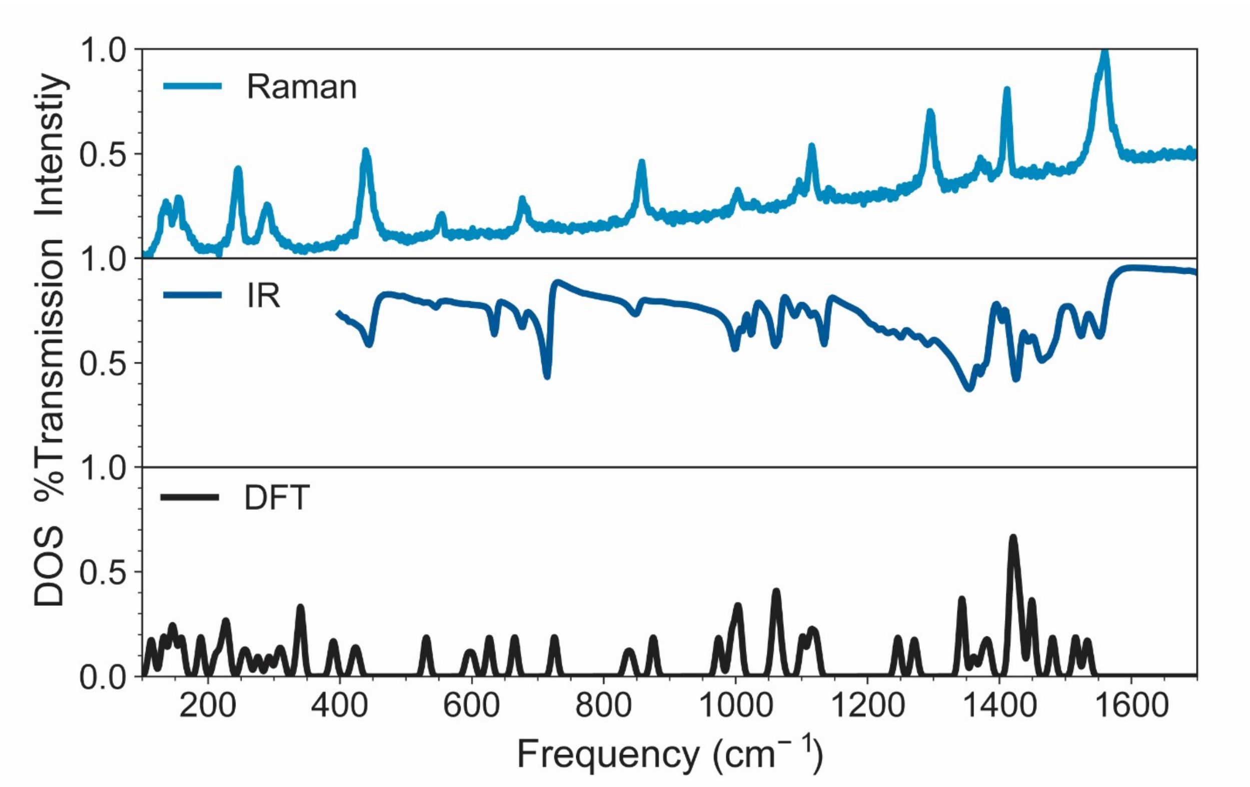

Figure S32 | Comparison of the experimental Raman spectroscopy normalized intensities, experimental FTIR spectroscopy percent transmission, and computed normalized phonon density of states for **1**. FTIR and Raman spectra were taken on powder samples at ambient temperature.

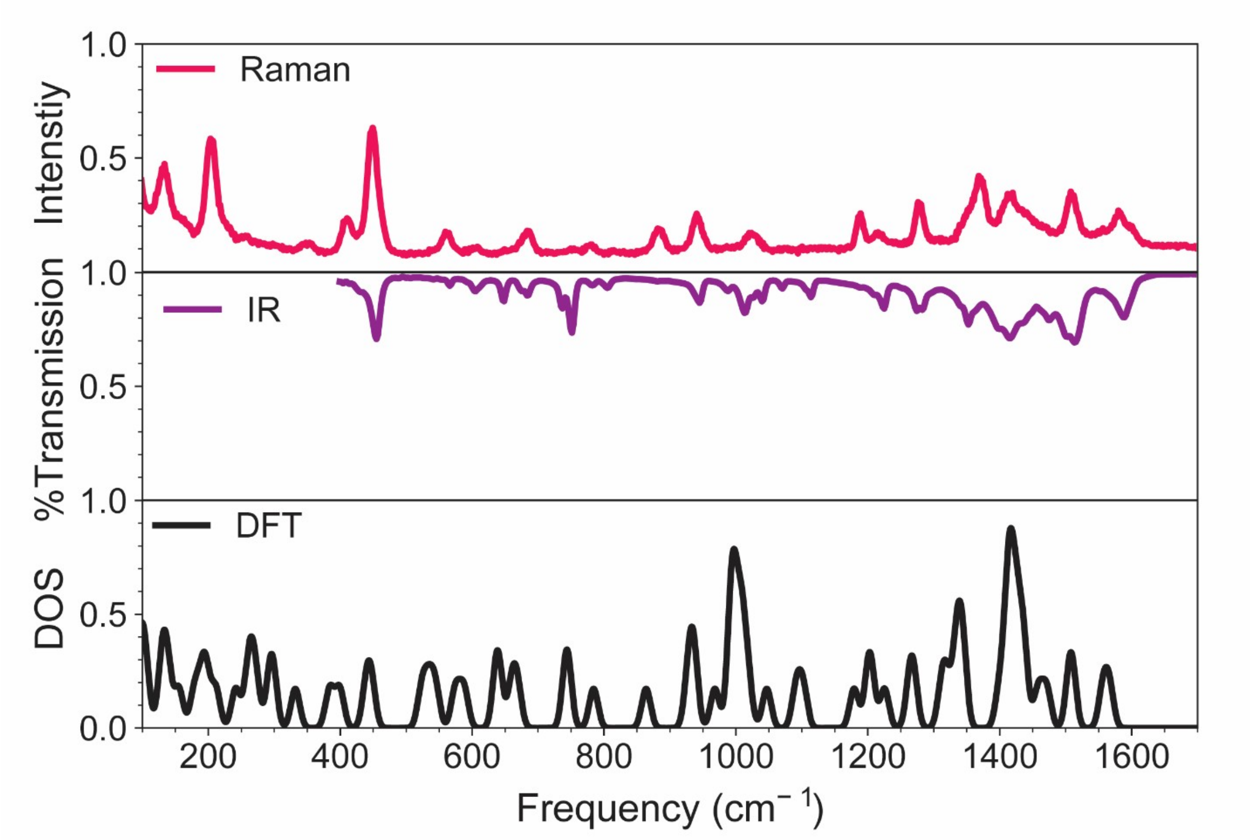

Figure S33 | Comparison of the experimental Raman spectroscopy normalized intensities, experimental FTIR spectroscopy percent transmission, and computed normalized phonon density of states for **2**. FTIR and Raman spectra were taken on powder samples at ambient temperature.

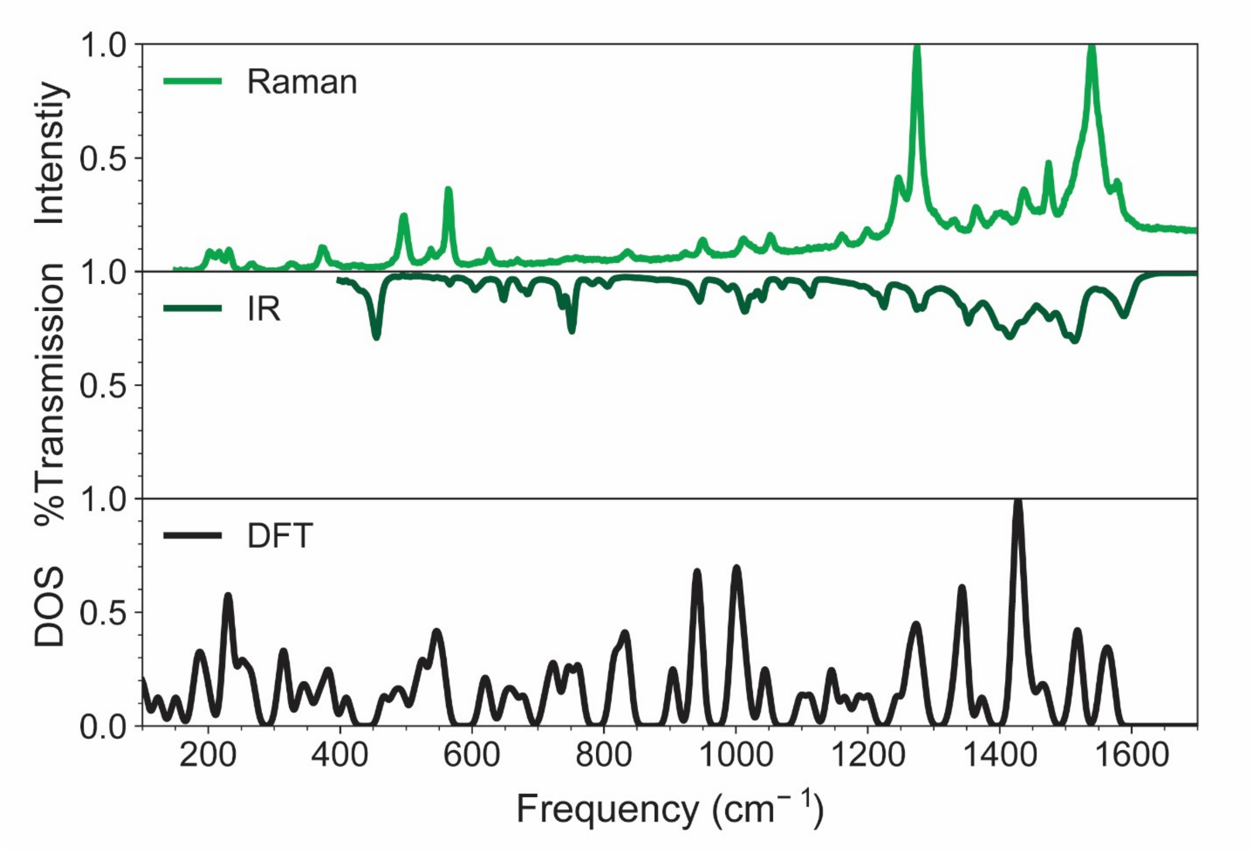

Figure S34 | Comparison of the experimental Raman spectroscopy normalized intensities, experimental FTIR spectroscopy percent transmission, and computed normalized phonon density of states for **3**. FTIR and Raman spectra were taken on powder samples at ambient temperature.

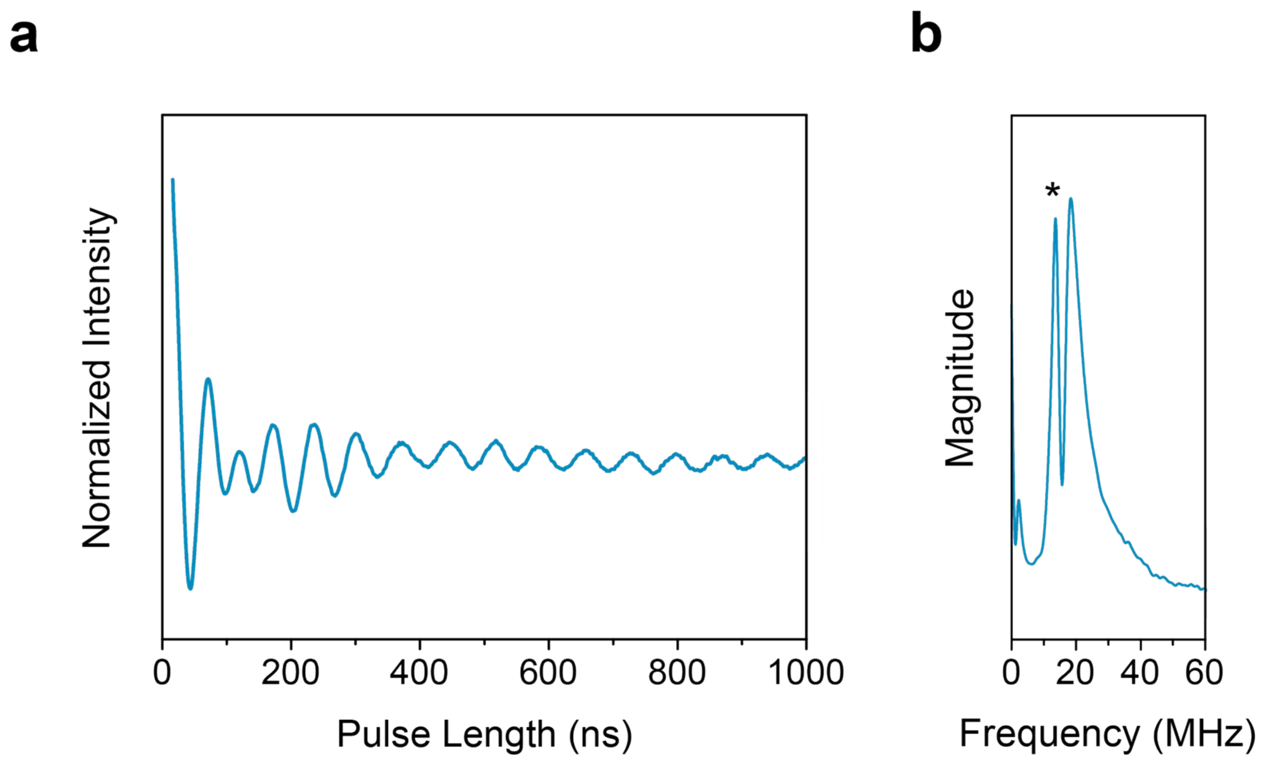

Figure S35 | (**a**) Rabi oscillations observed for **1'** at 5 K. (**b**) The Fourier transform of the nutation experiment. A second peak at 14.5 MHz (marked with an asterisk) is observed, corresponding to the Larmor frequency of ^{1}H. This secondary signal is a result of the Hartman-Hahn effect of the processing ^{1}H nucleus.[16]

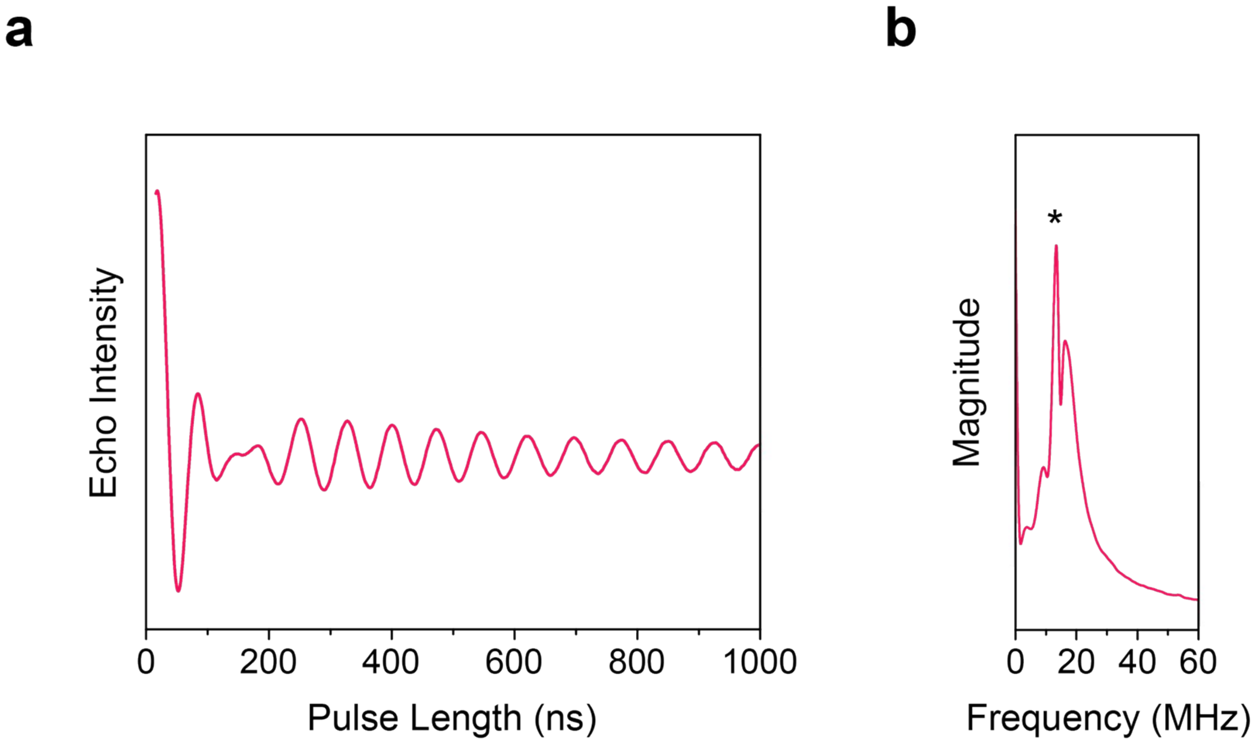

Figure S36 | (**a**) Rabi oscillations observed for **2'** at 5 K. (**b**) The Fourier transform of the nutation experiment. A second peak at 14.5 MHz (marked with an asterisk) is observed, corresponding to the Larmor frequency of ^{1}H. This secondary signal is a result of the Hartman-Hahn effect of the processing ^{1}H nucleus.[16] Additional low frequency, weak intensity peaks are artifacts of data processing.

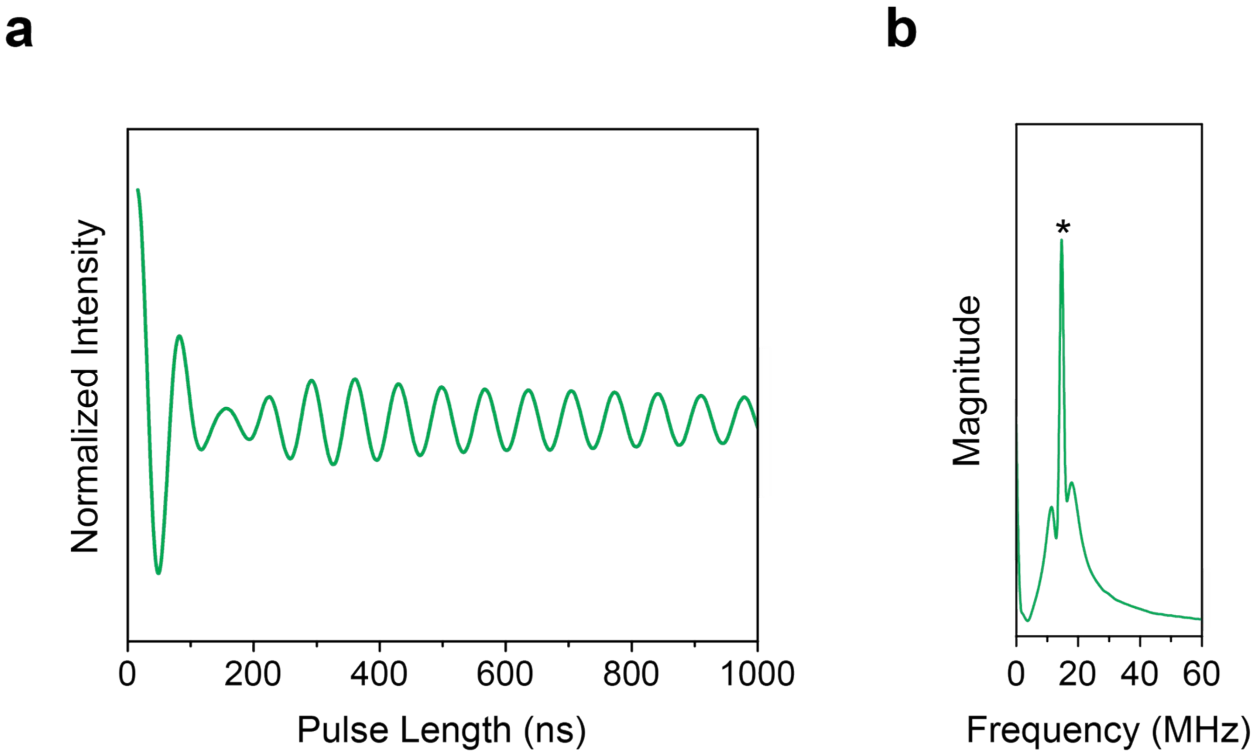

Figure S37 | (**a**) Rabi oscillations observed for **3'** at 5 K. (**b**) The Fourier transform of the nutation experiment. A second peak at 14.5 MHz (marked with an asterisk) is observed, corresponding to the Larmor frequency of ^{1}H. This secondary signal is a result of the Hartman-Hahn Effect of the processing ^{1}H nucleus.[16] Additional low frequency, weak intensity peaks are artifacts of data processing.

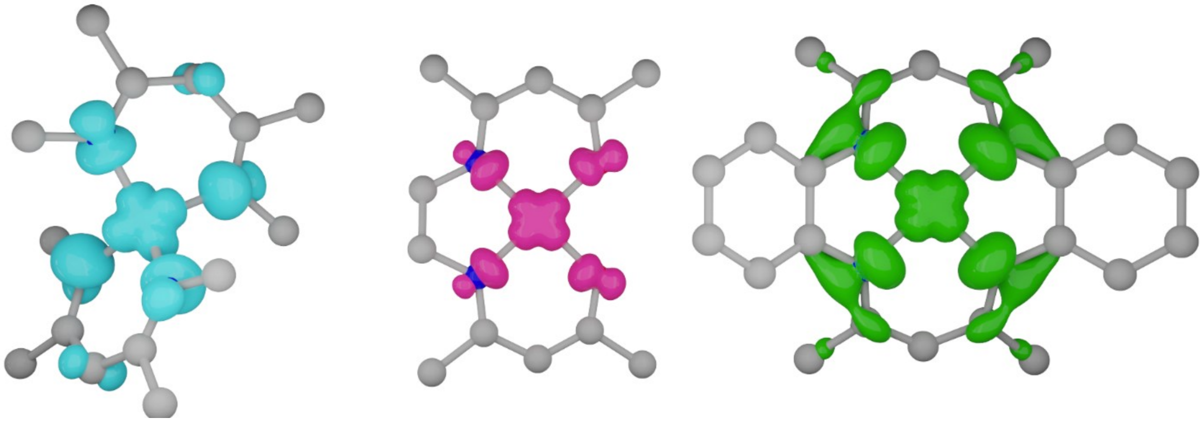

Figure S38 | Plots of spin densities from VASP calculations at the 70% occupancy level for **1**−**3**, with the spin densities located on the copper atom (ρ_{Cu}) and the average located on each donor atom ($\rho_{E,Avg}$). There is an increased spin density on donor atoms and on the copper center in **2** relatives to **3** due to the strong resonance effects present in **3** allowing for more delocalization of the spin onto the carbon backbone of the ligand. This places more spin density on the direct donor atoms in **2**. A weaker, though still present, resonance effect in **1** is also at play, explaining the comparatively similar ρ_{Cu} between **1** and **2**. Strong interactions with the ligand, as well this resonant effect, explain the comparatively delocalized spins in this report relative to others in the literature.[23]

References

1 M. M. Stalzer, T. L. Lohr and T. J. Marks, *Inorg. Chem.*, 2018, **57**, 3017–3024.
2 P. Mountford, *Chem. Soc. Rev.*, 1998, **27**, 105–115.
3 R. J. Greet and D. Turnbull, *Mol. J. Chem. Phys.*, 1967, **46**, 2372.
4 C. E. Jackson, C.-Y. Lin, S. H. Johnson, J. Van Tol and J. M. Zadrozny, , DOI:10.1039/c9sc02899d.
5 A. J. Fielding, S. Fox, G. L. Millhauser, M. Chattopadhyay, P. M. H. Kroneck, G. Fritz, G. R. Eaton and S. S. Eaton, *J Magn Reson*, 2006, **179**, 92–104.
6 L. R. Dalton, A. L. Kwiram and J. A. Cowen, *Chem. Phys. Lett.*, 1972, **14**, 77–81.
7 B. Biospin, .
8 Mathworks, .
9 S. Stoll and A. Schweiger, *J. Magn. Reson.*, 2006, **178**, 42–55.
10 OriginLab, .
11 H. Chen, A. G. Maryasov, O. Y. Rogozhnikova, D. V. Trukhin, V. M. Tormyshev and M. K. Bowman, *Phys. Chem. Chem. Phys.*, 2016, **18**, 24954–24965.
12 S. Pfenninger, W. E. Antholine, M. E. Barr, J. S. Hyde, P. M. Kroneck and W. G. Zumft, *Biophys. J.*, 1995, **69**, 2761–2769.
13 C. J. Yu, M. D. Krzyaniak, M. S. Fataftah, M. R. Wasielewski and D. E. Freedman, *Chem. Sci.*, 2019, **10**, 1702–1708.
14 M. J. Graham, M. D. Krzyaniak, M. R. Wasielewski and D. E. Freedman, *Inorg. Chem.*, 2017, **56**, 8106–8113.
15 M. Atzori, E. Morra, L. Tesi, A. Albino, M. Chiesa, L. Sorace and R. Sessoli, *J. Am. Chem. Soc.*, 2016, **138**, 11234–11244.
16 S. R. Hartmann and E. L. Hahn, *Phys. Rev.*, 1962, **128**, 2042–2053.
17 B. A. Inc., 2007.
18 Bruker AXS Inc, 2007.
19 G. M. Sheldrick, *Acta Crystallogr. Sect. A Found. Crystallogr.*, 2008, 64, 112–122.
20 O. V. Dolomanov, L. J. Bourhis, R. J. Gildea, J. A. K. Howard and H. Puschmann, *J. Appl. Crystallogr.*, 2009, **42**, 339–341.
21 G. Ricciardi, A. Bavoso, A. Rosa, F. Lelj and Y. Cizov, *J. Chem. Soc. Dalt. Trans.*, 1995, 2385–2389.
22 A. J. Fielding, S. Fox, G. L. Millhauser, M. Chattopadhyay, P. M. H. Kroneck, G. Fritz, G. R. Eaton and S. S. Eaton, *J. Magn. Reson.*, 2006, **179**, 92–104.
23 M. S. Fataftah, M. D. Krzyaniak, B. Vlaisavljevich, M. R. Wasielewski, J. M. Zadrozny and D. E. Freedman, *Chem. Sci.*, 2019, **10**, 6707–6714.
24 S. K. Hoffmann and S. Lijewski, *J. Magn. Reson.*, 2013, **227**, 51–56.
25 L. Escalera-Moreno, N. Suaud, A. Gaita-Ariñ and E. Coronado, *J. Phys. Chem. Lett*, 2017, **8**, 1695–1700.
26 G. R. Eaton and S. S. Eaton, *Biological Magnetic Resonance*, Kluwer Academic Publishers, 2000, vol. 19.
27 E. Garlatti, L. Tesi, A. Lunghi, M. Atzori, D. J. Voneshen, P. Santini, S. Sanvito, T. Guidi, R. Sessoli and S. Carretta, *Nat. Commun.*, , DOI:10.1038/s41467-020-15475-7.
28 P. C. Andrews, J. L. Atwood, L. J. Barbour, P. D. Croucher, P. J. Nichols, N. O. Smith, B. W. Skelton, A. H. White and C. L. Raston, *J. Chem. Soc. - Dalt. Trans.*, 1999, **17**, 2927–2932.
29 L. Yang, D. R. Powell and R. P. Houser, *Dalt. Trans.*, 2007, 955–964.
30 D. Rosiak, A. Okuniewski and J. Chojnacki, *Polyhedron*, 2018, **146**, 35–41.
31 G. Kresse and J. Hafner, *Phys. Rev. B*, 1993, **47**, 558–561.
32 G. Kresse and J. Hafner, *Phys. Rev. B*, 1994, **49**, 14251–14269.
33 G. Kresse and J. Furthmüller, *Comput. Mater. Sci.*, 1996, **6**, 15–50.

34 T. Frederiksen, M. Paulsson, M. Brandbyge and A. P. Jauho, *Phys. Rev. B - Condens. Matter Mater. Phys.*, 2007, **75**, 205413.
35 F. Neese, *Wiley Interdiscip. Rev. Comput. Mol. Sci.*, 2012, **2**, 73–78.
36 A. D. Becke, *J. Chem. Phys.*, 1993, **98**, 1372–1377.
37 C. Lee, W. Yang and R. G. Parr, *Phys. Rev. B*, 1988, **37**, 785–789.
38 S. H. Vosko, L. Wilk and M. Nusair, *Can. J. Phys.*, 1980, **58**, 1200–1211.
39 P. J. Stephens, F. J. Devlin, C. F. Chabalowski and M. J. Frisch, *J. Phys. Chem.*, 1994, **98**, 11623–11627.
40 F. Weigend, F. Furche and R. Ahlrichs, *J. Chem. Phys.*, 2003, **119**, 12753–12762.
41 F. Weigend, *Phys. Chem. Chem. Phys.*, 2006, **8**, 1057–1065.
42 N. P. Kazmierczak, R. Mirzoyan and R. G. Hadt, *J. Am. Chem. Soc.*, 2021, **143**, 17305–17315.
43 T. V. Basova, V. G. Kiselev, B.-E. Schuster, H. Peisert and T. ChassÃ©, *J. Raman Spectrosc.*, 2009, **40**, 2080–2087.
